# Discovery and Development of an Aerobic Radical Hydroxyarylation Reaction Using Aryl Halides, Olefins, and O_2_

**DOI:** 10.1021/acs.orglett.5c00968

**Published:** 2025-04-17

**Authors:** Mark C. Maust, Defne Tuncaral, Simon B. Blakey

**Affiliations:** Department of Chemistry, Emory University, Atlanta, Georgia 30322, United States

## Abstract

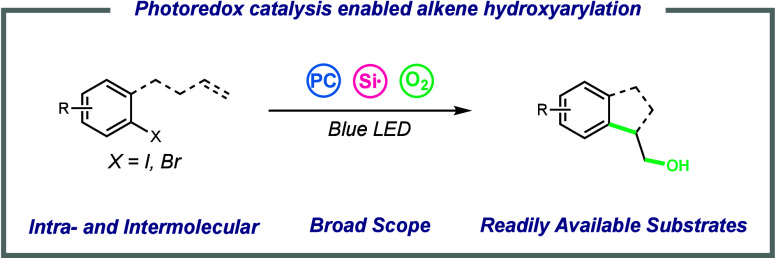

Herein, we report the development of a radical hydroxyarylation reaction through the coupling of an aryl radical, an olefin, and O_2_. Photoredox activation of a silyl radical halogen atom abstractor enables mild aryl radical generation and reactivity in 5-*exo*, 6-*exo*, and dearomative cyclizations to synthesize a variety of hydroxylated semisaturated fused ring systems. Expansion of the substrate scope to include aryl bromide starting materials reveals the crucial role of iodide in the catalytic cycle.

Semisaturated fused ring systems, such as indanes, indolines, dihydrobenzofurans, chromanes, tetrahydroisoquinolines, and tetralins, are widely occurring motifs in pharmaceutical and agrochemical agents (Figure 1A).^1^ Compared to their fully saturated analogues, these semisaturated systems have higher F(sp^3^) character, often imparting more desirable medicinal chemistry characteristics.^2^ Given their importance in biologically active molecules, various synthetic approaches for these scaffolds have been developed, including Friedel–Crafts alkylation,^3^ Diels–Alder cycloaddition,^4^ and intramolecular alkyl radical addition to an arene.^5^ Our group has developed an approach to construct these scaffolds through aryl radical cyclization onto a pendant olefin, inverting the intramolecular alkyl radical addition to an arene approach. This strategy leverages an expansive, easily accessible pool of aryl radical precursors combined with the flexibility to modify substituents of the pendant olefin. This work features a reagent-controlled switchable cyclization approach that enables selective access to either the 5-*exo* or 6-*endo* aryl radical cyclization product, enabling diversification of the same substrate into either the five- or six-membered semisaturated fused ring system, respectively (Figure 1B).^6^ Under the 6-*endo* selective conditions, radical generation occurs via proton-assisted single-electron reduction of an *N*-heteroaryl halide followed by homolytic cleavage of the carbon–halogen bond. While this method worked exceptionally well for *N*-heteroaryl species, attempts to extend the reaction scope to benzenoid substrates using these conditions failed with no hydrogen bond acceptor on the arene to facilitate single-electron reduction.

**Figure 1:**
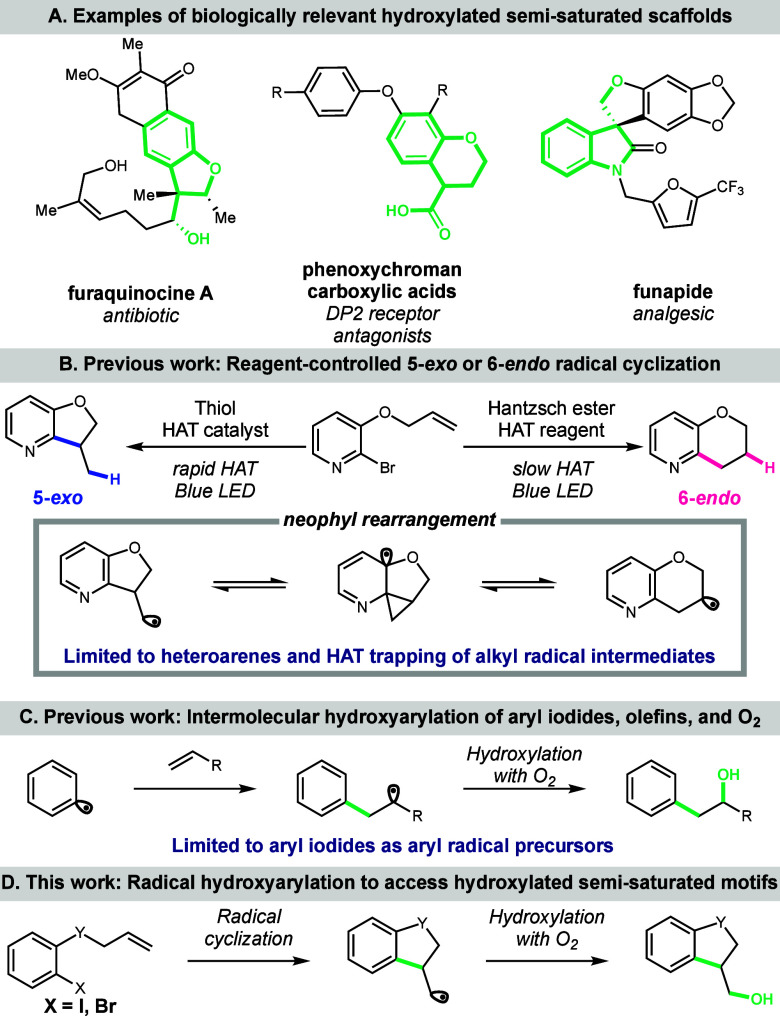
Background for designing an intra- and intermolecular hydroxyarylation reaction of aryl halides, olefins, and O_2_.

Given the incompatibility of nonheteroaryl halides with our single-electron reduction conditions, we envisioned halogen atom transfer (XAT) as an alternative mechanism for aryl radical generation.^7^ Notably, silyl radical intermediates are effective halogen atom abstractors of aryl halides under photoredox conditions.^8^ In considering this approach, we recognized that silyl radical XAT reagents are oxidatively activated by an excited state photocatalyst. In contrast to traditional reductive photoredox reactions, which are often incompatible with O_2_,^9^ these conditions would allow incorporation of O_2_ as a trapping reagent, enabling an olefin hydroxyarylation reaction under mild conditions.

We initially described this discovery in the context of an intermolecular three-component coupling between aryl iodides, olefins, and O_2_ (Figure 1C).^10^ However, we recognized that an intramolecular variant of this reaction would directly yield hydroxylated semisaturated ring systems (Figure 1D), which are present in a variety of pharmaceutically relevant compounds.^1^ Traditional preparation of these hydroxylated semisaturated ring systems relies on an aryl lithiate addition to a tethered epoxide.^11^ Alternatively, cascade cyclization/borylation techniques such as Pd-catalyzed domino Heck/arylborylation of aryl halides, radical cyclization/borylation of aryl diazonium salts, and TEMPO trapping of radical intermediates can be used.^12^ These examples are robust in aryl partners but require an additional synthetic step to either oxidize the boronate or reduce the TEMPO adduct to the desired alcohol. Other methods for the direct transformation of aryl halide precursors to hydroxylated benzocyclic scaffolds include Sn-mediated aryl halide abstraction^13^ and a recent report by Cornella et al., who describe a Ni-catalyzed O-atom insertion reaction utilizing N_2_O.^14^ In this report, we detail the development of our silyl radical-mediated XAT approach to construct hydroxylated semisaturated ring systems, complementing existing methods for accessing these motifs. Furthermore, in our original report,^10^ only aryl iodides could be used as aryl radical precursors. In this Letter, we describe the development of reaction conditions to further expand this scope to include more widely available aryl bromide starting materials.

We began our investigations into the hydroxyarylation reaction using modified silyl radical-mediated XAT conditions reported by ElMarrouni and Balsells.^15^ In a reaction vessel open to air, aryl iodide **1**, photocatalyst [Ir(dF(CF_3_)ppy)_2_(dtbbpy)]PF_6_, XAT reagent (TMS)_3_SiH, and Na_2_CO_3_ were irradiated by blue LEDs to give a mixture of the desired hydroxyaryl product **2** (32%) and undesired HAT termination product **3** (75%, Table 1, entry 1). The selectivity of the product outcome was switched from **3** (26%) to **2** (70%) by sparging with O_2_ gas prior to starting the reaction (Table 1, entry 2). We hypothesized that utilizing a silyl radical precursor with a less abstractable H atom would lead to a lower yield of HAT termination product **3** and a higher selectivity for hydroxyaryl product **2**. With tris(trimethylsilyl)silanol ((TMS)_3_SiOH) as the XAT reagent and a more oxidizing photocatalyst (Cl-4CzIPN), complete selectivity for the hydroxyaryl product was achieved (72% **2**, 0% **3**), though starting material remained at the end of the reaction (31% **1**, Table 1, entry 3). Increasing the Cl-4CzIPN loading from 1 to 5 mol % aided in complete starting material consumption to furnish hydroxyaryl product **2** in 97% yield (Table 1, entry 4). Control experiments showed that light, a photocatalyst, (TMS)_3_SiOH, and O_2_ were all necessary components for the desired reactivity (Table 1, entries 5–8, respectively).

**Table 1:**
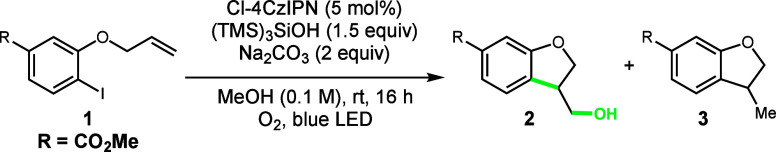

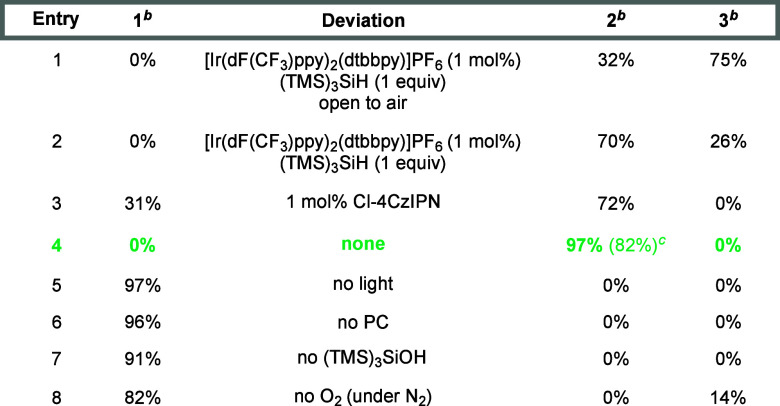
Optimization of the Intramolecular Aerobic Hydroxyarylation Reaction^a^

a Conditions: methyl-3-allyloxy-4-iodobenzoate (0.1 mmol), a photocatalyst, a silyl reagent, Na_2_CO_3_ (2 equiv), MeOH (1 mL), a blue LED, rt, 16 h.

b Yields determined by ^1^H NMR with an internal standard.

c Reaction performed on a 1 mmol scale with the isolated yield in parentheses.

With the optimized conditions in hand, we started investigating the reaction scope with respect to the olefin radical acceptor. Internal substitution of the olefin was well tolerated, generating the hydroxyaryl product containing an all-carbon quaternary center (Table 2, **4**, 56% yield). Mono- and disubstitution of the olefin terminus were also well tolerated to generate secondary alcohol **5** (66% yield) and tertiary alcohol **6** (86% yield), respectively. Radical addition to Michael acceptor type olefins often results in radical hydroarylation products due to the susceptibility of α-carbonyl radicals to reduction and subsequent protonation.^16^ Under our conditions, we observe almost exclusive formation of α-hydroxy carbonyl compound **7** and only trace amounts of the hydroarylation product (<5% observable by NMR). Other radical acceptors, such as the 2-position of indole, were also tolerated, resulting in dearomatization and hydroxylation of the benzylic position in **8** (67% yield).

**Table 2:**
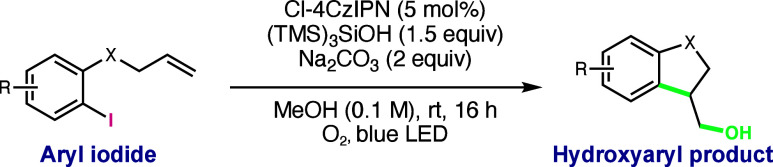

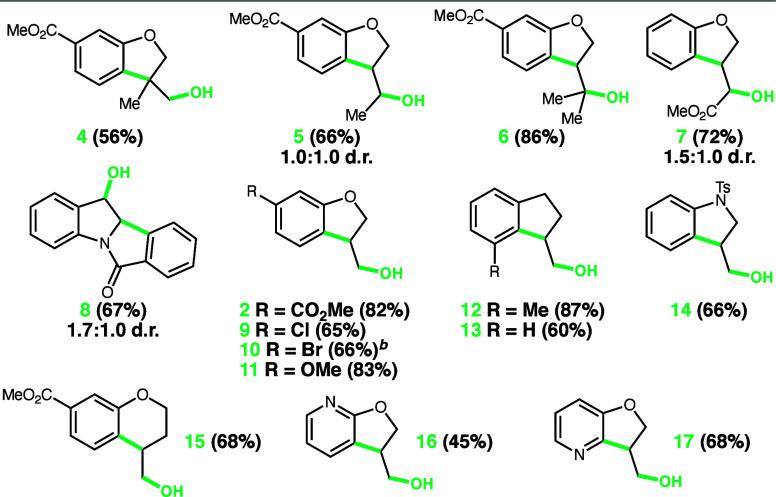
Scope of the Intramolecular Hydroxyarylation Reaction^a^

a Conditions: a substrate (0.5 mmol), Cl-4CzIPN (5 mol %), Na_2_CO_3_ (2 equiv), (TMS)_3_SiOH (1.5 equiv), MeOH (5 mL), O_2_, a blue LED, rt, 16 h. Isolated yields.

b With 1.0 equiv of (TMS)_3_SiOH.

With the scope of olefin substitution complete, we next began investigating arene substituents and their impact on product formation. Electron-deficient, electron-rich, and halogenated arenes all gave the corresponding hydroxyarylation products in good yields (**2** and **9–11**, 65–83%). Notably, a lower loading of the (TMS)_3_SiOH reagent was necessary for **10** to prevent abstraction of the bromide substituent, additionally resulting in starting material composing the rest of the reaction mass balance. Utilizing a radical halogen atom abstraction approach allows tolerance of *ortho*-substituted aryl halides (**12**, 87% yield), which is often a limitation encountered in reactions that require an oxidative addition step.^14^ The atom linking the arene and tethered olefin can be substituted for C to generate hydroxylated indane **13** (60% yield) and N to generate hydroxylated indoline **14** (66% yield). The carbon chain linking the olefin and arene can also be extended by one carbon to deliver 6-*exo* product **15** in 68% yield, providing access to hydroxylated chromane derivatives. Additionally, heteroaryl radicals derived from 3-iodo and 2-iodo pyridine cyclized smoothly to give products **16** and **17** in 45% and 68% yields, respectively. In some cases, lower yields are associated with co-elution of an unidentified byproduct during purification leading to not all of the product observed by NMR being isolated in pure form (such as in substrate **16**).

Aryl bromides are generally more commercially available and synthetically accessible compared to aryl iodides, making their incorporation into the reaction scope highly desirable. Unfortunately, in our previously reported intermolecular hydroxyarylation reaction,^10^ only trace amounts of hydroxyaryl product **20** are observed when utilizing aryl bromide **18** (Scheme 1, top). Similarly, the intramolecular variant reported in this work exhibits the same loss of reactivity when switching from aryl iodide to aryl bromide (Scheme 1, bottom). Given that silyl radical-mediated halogen atom abstraction has been demonstrated as an effective method for aryl radical generation from both aryl iodides and aryl bromides, the stark reactivity difference of the two halides observed in our system led us to question the role of iodide in our reaction mechanism.

**Scheme 1:**
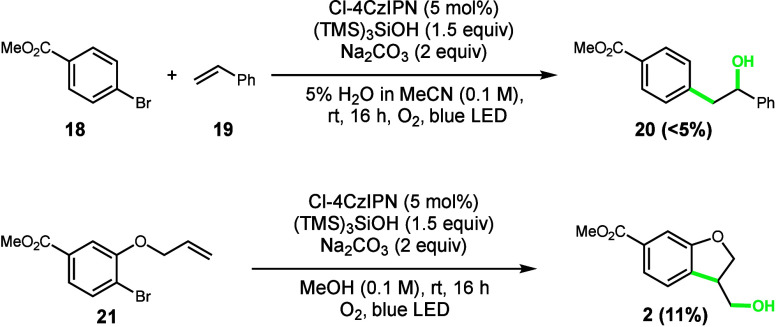
Investigation of Aryl Bromide Substrates Top conditions: a substrate (0.1 mmol), styrene (3 equiv), Cl-4CzIPN (5 mol %), Na_2_CO_3_ (2 equiv), (TMS)_3_SiOH (1.5 equiv), MeOH (1 mL), O_2_, a blue LED, rt, 16 h. Bottom conditions: a substrate (0.1 mmol), styrene (3 equiv), Cl-4CzIPN (5 mol %), Na_2_CO_3_ (2 equiv), (TMS)_3_SiOH (1.5 equiv), MeOH (1 mL), open to air, a blue LED, rt, 16 h. Yields determined by ^1^H NMR using an internal standard.

Depicted in Figure 2 is our proposed reaction mechanism, based on our own experimental findings in combination with literature precedent.^8,17^ The reaction is initiated by oxidation of (TMS)_3_SiOH (*E*_p_ = +1.54 V vs SCE)^8a,8b^ and subsequent radical Brook rearrangement to generate intermediate **I**. Halogen atom abstraction of the aryl halide generates aryl radical **II**, which engages with an olefin in either inter- or intramolecular addition to forge a C–C bond and alkyl radical intermediate **III**. Trapping of **III** with triplet O_2_ forms the C–O bond and peroxyl radical species **IV**. Reduction of **IV** by the photocatalyst radical anion closes the catalytic cycle and generates peroxide **V**.^17^ A second reduction is required to reduce peroxide **V** to the alcohol observed at the end of our reaction. We hypothesize that iodide formed from hydrolysis of the halosilane intermediate formed from the XAT step is the electron donor required for the reduction of peroxide **V** to the hydroxyaryl product.^18^

**Figure 2:**
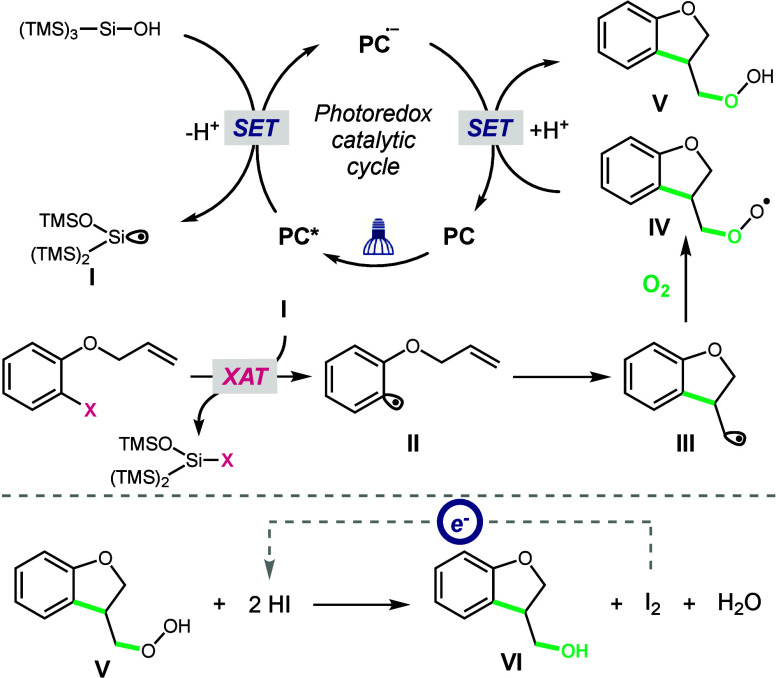
Mechanistic proposal for the hydroxyarylation reaction.

To probe our hypothesis of iodide being a crucial reductant in this reaction, we subjected **18** and styrene to the hydroxyarylation conditions in the presence of 1 equiv of tetrabutylammonium iodide (TBAI) as an additive and observed the formation of intermolecular hydroxyarylation product **20** in 36% yield with the rest of the reaction mass balance being unconsumed starting material (Table 3, entry 1). With the iodide additive improving the yield of **20**, we increased the additive loading to 2 equiv and saw decreased starting material consumption and product yield (15%, Table 1, entry 2). We presume the decreased yield was from iodide (*E*_p_ = +0.26 V vs SCE)^19^ acting as a competitive quencher of the excited state photocatalyst. Consistent with this hypothesis, reducing the TBAI loading to 0.25 equiv improved the yield of **20** to 50%. Switching silyl radical precursors to 1-adamantyl aminosilane ((TMS)_3_SiNHAdm) with a lower oxidation potential (*E*_p_ = +0.86 V vs SCE)^8c^ further increased the yield to 60%. To account for the substoichiometric loading of TBAI, we increased loading of the aminosilane reductant to 3 equiv and switched to a more soluble Ir photocatalyst ([Ir(dF(CF_3_)ppy)_2_(5,5′-dCF_3_bpy)]PF_6_). Under these conditions, full consumption of the aryl bromide starting material and a 71% yield of **20** were obtained. Although the improved yields upon addition of iodide to reaction mixtures using aryl bromide substrates is consistent with the hypothesis of peroxide formation, we did not directly observe the peroxide products.

**Table 3:**
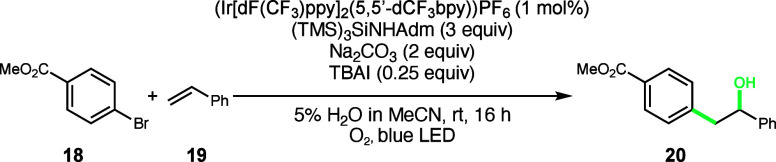

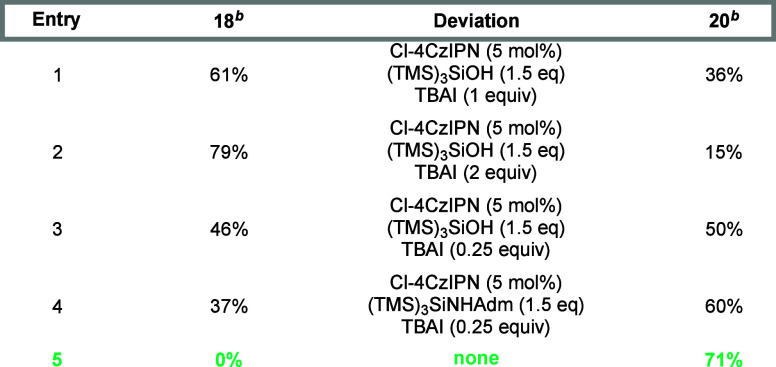
Optimization of the Hydroxyarylation Reaction Using Aryl Bromide Substrates^a^

a Conditions: methyl 4-bromobenzoate (0.1 mmol), styrene (3 equiv), a photocatalyst, TBAI (0.25 equiv) Na_2_CO_3_ (2 equiv), a silyl reagent, 5% H_2_O in MeCN (1 mL), a blue LED, rt, 16 h.

b Yields determined by ^1^H NMR with an internal standard.

With conditions that can now access hydroxyaryl products from aryl bromides, we chose a representative sample of aryl bromide substrates to compare the reactivity of the two aryl halide classes. In the intramolecular reaction, the aryl bromides of electron-rich, electron-deficient, and heteroaryl substates all produced hydroxyaryl products in nearly identical yields to the analogous aryl iodide substrates (Table 4, **2**, **11**, and **17**, respectively, 67–82%). The intermolecular conditions displayed a noticeable decrease in reaction efficiency, though synthetically useful yields of the hydroxyaryl products were still obtained from the reactions of 4-bromobenzotrifluoride (**22**, 42% yield) and tosylated 5-bromoindole (**23**, 48% yield) with styrene. Alternative olefin radical acceptors, such as ethyl acrylate, can also be used to generate α-hydroxy carbonyl product **24** in 29% yield. Additionally, combining the intra- and intermolecular reactions results in a cascade reaction in which initial 5-*exo* cyclization followed by hydroxyalkylation with styrene as the radical acceptor generates **25** in 39% yield.

**Table 4:**
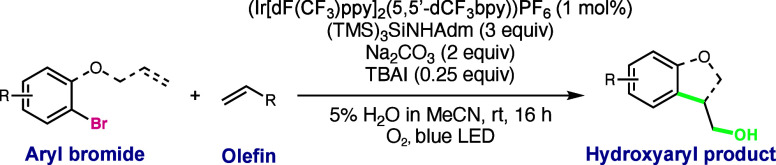

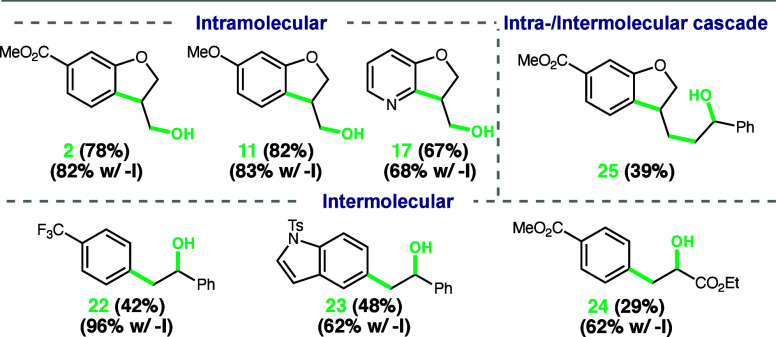
Scope of the Hydroxyarylation Reaction with Aryl Bromides^a^

a Conditions: methyl 4-bromobenzoate (0.1 mmol), an alkene (if intermolecular, 3 equiv), (Ir[dF(CF_3_)ppy]_2_(5,5′-dCF_3_bpy))PF_6_ (1 mol %), TBAI (0.25 equiv), Na_2_CO_3_ (0.2 mmol), (TMS)_3_SiNHAdm (3.0 equiv), 5% H_2_O in MeCN (1 mL), a blue LED, rt, 16 h. Isolated yields.

In summary, we have developed a mild, photoredox-driven approach to access hydroxylated semisaturated fused ring compounds. Our approach utilizes silyl radical-mediated halogen atom abstraction to access a broad range of aryl radical intermediates capable of undergoing C–C bond formation with an olefin and subsequent C–O bond formation with O_2_. Through developing a mechanistic understanding of this reaction, we were able to expand the reaction scope beyond aryl iodides to include more synthetically accessible and commercially available aryl bromides, broadening the potential impact of this method in the synthesis of complex molecules.

## Data Availability

The data underlying this study are available in the published article and its Supporting Information.

## Supporting Information Available

Experimental procedures, characterization data, and spectra (PDF)
