# Supplementary material for: Discovery and Development of an Aerobic Radical Hydroxyarylation Reaction Using Aryl Halides, Olefins, and O2
Source: Org Lett. 2025 Apr 17;27(17):4491–5. doi: 10.1021/acs.orglett.5c00968 (PMC12053939; doi:10.1021/acs.orglett.5c00968)
Supplement: Supplementary file 1 — ol5c00968_si_001.pdf [file ol5c00968_si_001.pdf]

Supporting Information

Discovery and Development of an Aerobic Radical Hydroxyarylation Reaction Using Aryl Halides, Olefins, and O_2

Mark C. Maust,[‡] Defne Tuncaral,[‡] and Simon B. Blakey.[*]

Corresponding author: sblakey@emory.edu

Department of Chemistry, Emory University, Atlanta, Georgia 30322, United States

I. *General information*
  a. **General Reagent Information (S2)**
  b. **General Analytical Information (S2)**

II. *General Procedures*
  a. **Optimization Procedures (S2)**
  b. **General Procedure A (S3)**
  c. **General Procedure B (S3)**
  d. **General Photoredox Setup (S4)**

III. *Extended Optimization*
  a. **Extended optimization of aryl iodide hydroxyarylation (S5)**
  b. **Extended optimization of aryl bromide hydroxyarylation (S6)**
  c. **Investigation of PPh_3 as a reductant for aryl bromide hydroxyarylation (S6)**

IV. *Preparation of Starting Materials and Reagents*
  a. **Preparation of Cl-4CzIPN (S7)**
  b. **Preparation of $(TMS)_3SiOH$ (S7)**
  c. **Preparation of $(TMS)_3SiNHAdm$ (S7)**
  d. **Preparation of substrates (S8-S12)**

V. *Preparation of Products from Table 2 (S12-S16)*

VI. *Preparation of Products from Table 4 (S17-S18)*

VII. *References (S18)*

VIII. *NMR Spectra(S20-S41)*

I. General Information

General Reagent Information:

Reagents were purchased from Sigma-Aldrich, Alfa Aesar, Acros Organics, Combi-Blocks, Oakwood Chemicals, Ambeed, and TCI America and used as received, unless stated otherwise. All reactions were carried out under nitrogen atmosphere with anhydrous solvents in oven- or flame-dried glassware using standard Schlenk technique, unless otherwise stated. Reactions performed at temperatures above room temperature were done so utilizing an appropriately sized heating mantle. Alkenes that were packaged with radical inhibitors were passed through a pipette plug of aluminum oxide, activated, Brockman I immediately prior to reaction set-up to remove inhibitor prior to adding to the reaction. Reactions requiring irradiation were done so with blue LEDs (Hydrofarm® PPB1002 PowerPAR LED Bulb-Blue 15W/E27 (available from Amazon). Flash chromatography was carried out using Siliaflash® P60 silica gel obtained from Silicycle. Thin-layer chromatography (TLC) was performed on 250 μm SiliCycle silica gel F-254 plates. Visualization of the developed chromatogram was performed by fluorescence quenching or staining using $KMnO_4$. Anhydrous dichloromethane (DCM), diethyl ether (Et_2O), tetrahydrofuran (THF), and toluene were obtained by passage through activated alumina using a Glass Contours solvent purification system.

General Analytical Information:

Unless otherwise noted, all yields refer to chromatographically and spectroscopically (^1H NMR) homogenous materials. New compounds were characterized by NMR and HRMS. ^1H and ^{13}C NMR spectra were obtained from the Emory University NMR facility and recorded on a Bruker Avance III HD 600 equipped with cryo-probe (600 MHz), Bruker 400 (400 MHz), INOVA 600 (600 MHz), INOVA 500 (500 MHz), INOVA 400 (400 MHz), or VNMR 400 (400 MHz), and are internally referenced to residual proton solvent signals. Data for ^1H NMR are reported as follows: chemical shift (ppm), multiplicity (s = singlet, d = doublet, t = triplet, q = quartet, m = multiplet, dd = doublet of doublets, dt = doublet of triplets, ddd= doublet of doublet of doublets, dtd= doublet of triplet of doublets, b = broad, etc.), coupling constant (Hz), integration, and assignment, when applicable. Data for decoupled ^{13}C NMR are reported in terms of chemical shift and multiplicity when applicable. High Resolution mass spectra were obtained from the Emory University Mass Spectral facility using a Thermo Scientific Extractive Plus with an orbitrap mass analyzer. The emission spectrum for the Hydrofarm® PPB1002 PowerPAR LED Bulb-Blue 15W/E27 light source was obtained using an Ocean Optics USB4000 spectrophotometer.

II. General Procedures

Optimization General Procedure for Table 1

An 8 mL screw-top reaction tube equipped with a stir bar was charged with Photocatalyst (1 mol%), Na_2CO_3 (2 equiv), aryl iodide (1.0 equiv). Solvent was added to the reaction tube and the tube was capped with a PTFE/silicon. The reaction was sparged with a balloon of O_2 for 10 minutes. The silyl reagent was added via syringe after sparging. The resulting mixture was stirred at room temperature at 1000 RPM for 16 h under irradiation by an overhead blue LED lamp located ~6 inches above the reaction. The crude reaction mixture was diluted with EtOAc (5 mL), passed through a pad of silica, and the filtrate was concentrated *in vacuo*. Dibromomethane (0.1 mmol, 7 μL) as internal standard was added and the crude sample was analyzed by ^1H NMR (d = 5s) to obtain reaction yield.

Optimization General Procedure for Table 3

An 8 mL screw-top reaction tube equipped with a stir bar was charged with Photocatalyst (1 mol%), Na_2CO_3 (2 equiv), aryl iodide (1.0 equiv), TBAI (0.25 equiv), and silyl reagent (if solid). Solvent was added to the reaction tube and the tube was capped with a PTFE/silicon. The reaction was sparged with a balloon of O_2 for 10 minutes. The silyl reagent was added via syringe (if liquid) after sparging. The resulting mixture was stirred at room temperature at 1000 RPM for 16 h under irradiation by an overhead blue LED lamp located ~6 inches above the reaction. The crude reaction

mixture was diluted with EtOAc (5 mL), passed through a pad of silica, and the filtrate was concentrated *in vacuo*. Dibromomethane (0.1 mmol, 7 μL) as internal standard was added and the crude sample was analyzed by ^{1}H NMR (d = 5s) to obtain reaction yield.

General Procedure A

A 20 mL screw-top reaction tube equipped with a stir bar was charged with Cl-4CzIPN (5 mol%), Na_2CO_3 (2 equiv), aryl iodide (1.0 equiv). Benchtop MeOH (0.1 M) was added to the reaction and the tube was capped with a screw on PTFE/silicon cap. The reaction was sparged with a balloon of O_2 for 10 minutes then $(TMS)_3SiOH$ (1.5 equiv) was added via syringe. The resulting mixture was stirred at 1000 RPM for 16 h under irradiation by blue LEDs at room temperature. The crude reaction mixture was transferred to a round bottom flask and the reaction solvent was removed via rotatory evaporation. The resulting residue was diluted with 20 mL of water, 20 mL of EtOAc, and extracted 3x with EtOAc (3x 20 mL). The combined organic layers were washed with brine (1x 20 mL), dried over Na_2SO_4, and concentrated via rotatory evaporation. The crude sample was purified by silica chromatography using the indicated solvent mixture as the eluent to afford the title compound.

General Procedure B

An 8-mL screw-top reaction tube equipped with a stir bar was charged with $(Ir[dF(CF_3)ppy]_2(5,5'\text{-}dCF_3bpy))PF_6$ (1 mol%), $(TMS)_3SiNHAdm$ (3 equiv), Na_2CO_3 (2 equiv), aryl iodide (1.0 equiv), and TBAI (0.25 equiv). 5% H_2O in MeCN (0.1 M) was added to the reaction tube and the tube was capped with a screw on PTFE/silicon cap. The reaction was sparged with a balloon of O_2 for 10 minutes. If the reaction was an intermolecular coupling, alkene was added via syringe following sparging. The resulting mixture was stirred at 1000 RPM for 16 h under irradiation by blue LEDs at room temperature. The crude reaction mixture was transferred to a round bottom flask and the reaction solvent was removed via rotatory evaporation. The resulting residue was diluted with 20 mL of water, 20 mL of EtOAc, and extracted 3x with EtOAc (3x 20 mL). The combined organic layers were washed with brine (1x 20 mL), dried over Na_2SO_4, and concentrated via rotatory evaporation. The crude sample was purified by silica chromatography using the indicated solvent mixture as the eluent to afford the title compound.

General Photoredox Set-up:

The reactions were conducted in a solvent cabinet fitted with a large fan, cutout holes to maintain reactions at room temperature, and reflective tape (photos 1-4). The reactions were placed in an appropriately sized 3D printed carousel, allowing multiple reactions to be run simultaneously. A 15W blue LED array lamp was placed 6 inches above the reactions.

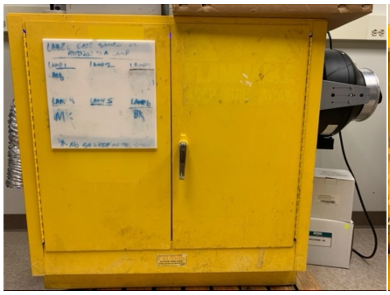

Photo 1

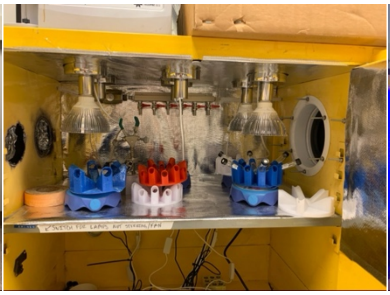

Photo 2

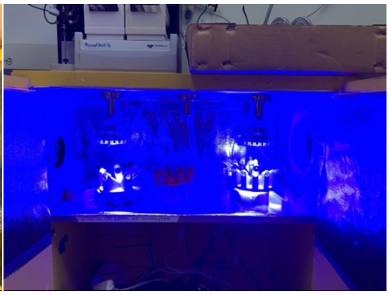

Photo 3

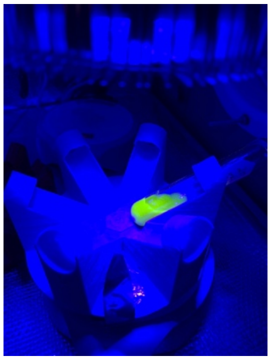

Photo 4

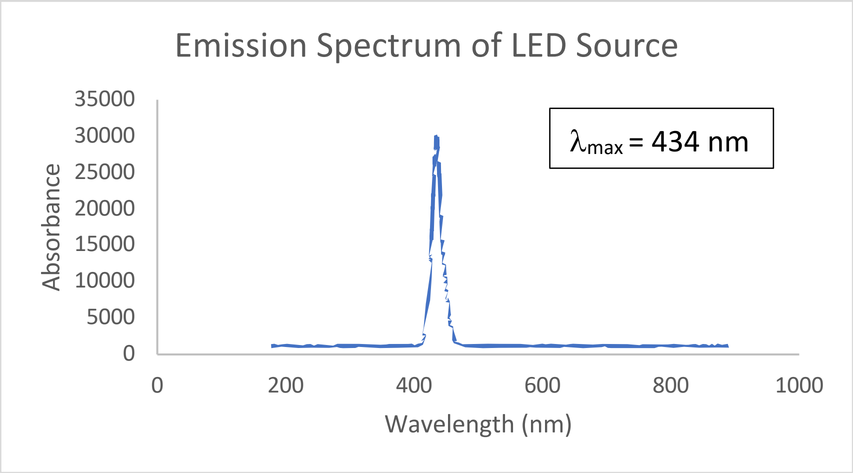

III. Extended Optimization

Extended Optimization for Aryl Iodide Hydroxyarylation

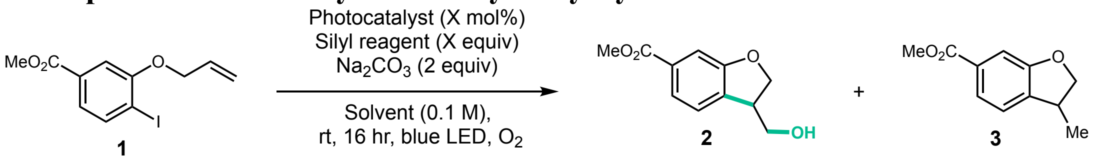

Entry	1	Photocatalyst	Silyl Reagent	Solvent	2 Yield	3 Yield
1	0%	[Ir(dF(CF$_3$)ppy)$_2$(dtbbpy)]PF$_6$ (1 mol%)	(TMS)$_3$SiH (1 equiv)	MeOH	70%	26%
2	30%	Ph-Mes-AcrBF$_4$ (1 mol%)	(TMS)$_3$SiH (1 equiv)	MeOH	70%	0%
3	0%	4CzIPN (1 mol%)	(TMS)$_3$SiH (1 equiv)	MeOH	82%	21%
4	36%	[Ir(dF(CF$_3$)ppy)$_2$(dtbbpy)]PF$_6$ (1 mol%)	(TMS)$_3$SiH (1 equiv)	DMF	61%	7%
5	0%	[Ir(dF(CF$_3$)ppy)$_2$(dtbbpy)]PF$_6$ (1 mol%)	(TMS)$_3$SiH (1 equiv)	DMSO	65%	15%
6	0%	[Ir(dF(CF$_3$)ppy)$_2$(dtbbpy)]PF$_6$ (1 mol%)	(TMS)$_3$SiH (1 equiv)	Toluene	17%	41%
7	0%	[Ir(dF(CF$_3$)ppy)$_2$(dtbbpy)]PF$_6$ (1 mol%)	(TMS)$_3$SiH (1 equiv)	DCM	0%	15%
8	40%	Cl-4CzIPN (1 mol%)	(TMS)$_3$SiOH (1.1 equiv)	MeOH	64%	0%
9	63%	Cl-4CzIPN (1 mol%)	(TMS)$_3$SiOH (1.1 equiv)	MeCN	22%	0%
10	72%	Cl-4CzIPN (1 mol%)	(TMS)$_3$SiOH (1.1 equiv)	Acetone	19%	0%
11	31%	Cl-4CzIPN (1 mol%)	(TMS)$_3$SiOH (1.5 equiv)	MeOH	72%	0%
12	25%	Cl-4CzIPN (1 mol%)	(TMS)$_3$SiOH (3.0 equiv)	MeOH	81%	0%
13	10%	Cl-4CzIPN (1 mol%)	(TMS)$_3$SiOH (5.0 equiv)	MeOH	82%	0%
14	0%	Cl-4CzIPN (5 mol%)	(TMS)$_3$SiOH (1.5 equiv)	MeOH	97%	0%
15	0%	[Ir(dF(CF$_3$)ppy)$_2$(dtbbpy)]PF$_6$ (1 mol%)	(TMS)$_3$SiNHAdm (1.5 equiv)	MeOH	83%	0%
16	0%	4CzIPN (1 mol%)	(TMS)$_3$SiNHAdm (1.5 equiv)	MeOH	75%	0%
17	0%	Cl-4CzIPN (1 mol%)	(TMS)$_3$SiNHAdm (1.5 equiv)	MeOH	52%	11%

Extended Optimization for Aryl Bromide Hydroxyarylation

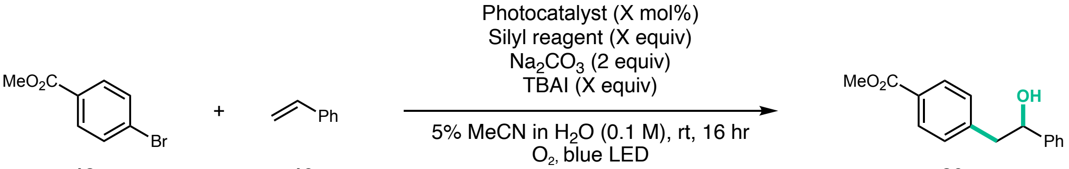

18 **19** **20**

Entry	18	Photocatalyst	Silyl Reagent	TBAI loading	20 Yield
1	61%	Cl-4CzIPN (5 mol%)	$(TMS)_3SiOH$ (1.5 equiv)	1 equiv	36%
2	79%	Cl-4CzIPN (5 mol%)	$(TMS)_3SiOH$ (1.5 equiv)	2 equiv	15%
3	86%	Cl-4CzIPN (5 mol%)	$(TMS)_3SiOH$ (1.5 equiv)	1 equiv	12%
4	48%	Cl-4CzIPN (5 mol%)	$(TMS)_3SiOH$ (2 equiv)	1 equiv	50%
5	27%	Cl-4CzIPN (5 mol%)	$(TMS)_3SiOH$ (3 equiv)	1 equiv	61%
6	17%	Cl-4CzIPN (5 mol%)	$(TMS)_3SiOH$ (2 equiv)	0.75 equiv	50%
7	20%	Cl-4CzIPN (5 mol%)	$(TMS)_3SiOH$ (2 equiv)	0.5 equiv	51%
8	16%	Cl-4CzIPN (5 mol%)	$(TMS)_3SiOH$ (2 equiv)	0.25 equiv	50%
9	37%	Cl-4CzIPN (5 mol%)	$(TMS)_3SiNHAdm$ (1.5 equiv)	0.25 equiv	60%
10	37%	Cl-4CzIPN (5 mol%)	$(TMS)_3SiNHAdm$ (1.5 equiv)	0.25 equiv	60%
11	0%	Cl-4CzIPN (5 mol%)	$(TMS)_3SiNHAdm$ (3 equiv)	0.25 equiv	69%
12	43%	$[Ir(dF(CF_3)ppy)_2(dtbbpy)]PF_6$ (1 mol%)	$(TMS)_3SiNHAdm$ (3 equiv)	0.25 equiv	59%
13	0%	$(Ir[dF(CF_3)ppy]_2(5,5'\text{-}dCF_3bpy))PF_6$ (1 mol%)	$(TMS)_3SiNHAdm$ (3 equiv)	0.25 equiv	71%
14	27%	$(Ir[dF(CF_3)ppy]_2(5,5'\text{-}dCF_3bpy))PF_6$ (1 mol%)	$(TMS)_3SiNHAdm$ (1.5 equiv)	0.25 equiv	62%

Investigation of PPh_3 as a reductant for Aryl Bromide Hydroxyarylation

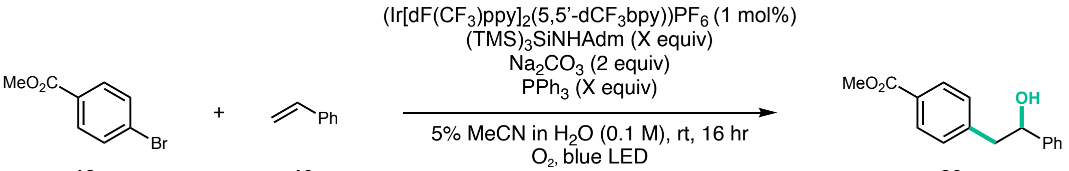

18 **19** **20**

Entry	18	PPh_3 loading	$(TMS)_3SiNHAdm$ loading	20 Yield
1	36%	0.25 equiv	2.5 equiv	34%
2	32%	0.5 equiv	2.5 equiv	20%
3	33%	1.0 equiv	2.5 equiv	12%
4	42%	1.0 equiv	1.0 equiv	16%
5	35%	1.0 equiv	1.5 equiv	14%
6	36%	1.0 equiv	2.0 equiv	11%

IV. Preparation of Starting Materials and Reagents

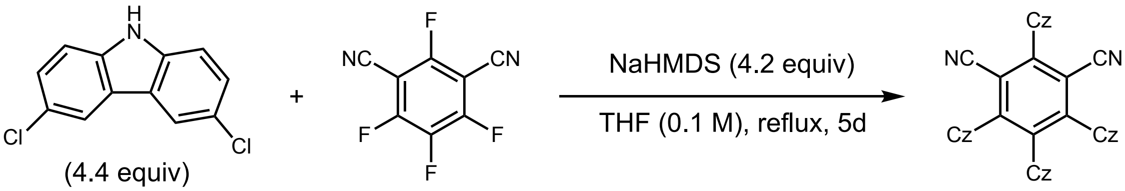

2,4,5,6-Tetrakis(3.6-dichloro-9H-carbazol-9-yl)isopthalonitrile (Cl-4CzIPN):
The following procedure was modified from a previous reported procedure by Molander et al.[1] An oven-dried 100 mL round-bottom flask was equipped with a stir bar and charged with 3,6-dichloro-9H-carbazole (4.155 g, 17.6 mmol, 4.4 equiv). The flask was sealed with a septum and the atmosphere was exchanged by applying vacuum and backfilling with nitrogen (this process was conducted a total of three times). Under nitrogen atmosphere, THF (40 mL) was added and the flask was cooled to 0 °C. Once cooled, a solution of NaHMDS in THF (16.8 mL, 16.8 mmol, 4.2 equiv, 1 M in THF) was added slowly. After complete addition, the mixture was stirred for 10 minutes at 0 °C followed by 30 minutes at room temperature. After this time, 2,4,5,6-tetrafluoroisopthalonitrile was added in one portion. The flask was then heated to reflux for 5 days.

After 5 days, the reaction was cooled to room temperature and filtered through a large coarse fritted funnel. The solid was washed with Et_2O (~500 mL) and the filtrate was discarded. The solid was then washed with $CHCl_3$ (~500 mL) to elute the desired product. The filtrate was transferred to a round-bottom flask and the solvent was removed *in vacuo*. The resulting solid was washed with a 75:25 mixture of pentanes/acetone (2 x 100 mL), followed by pentane (100 mL). The resulting bright yellow solid was dried under vacuum to give 2,4,5,6-Tetrakis(3.6-dichloro-9H-carbazol-9-yl)isopthalonitrile (2.334 g, 55%). The physical properties and spectral data were consistent with those reported in the literature.[1]

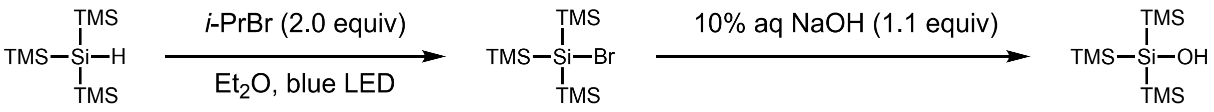

Tris(trimethylsilyl)silanol ($(TMS)_3SiOH$): The following procedure was modified from a previous reported procedure by MacMillan et al.[2] Under air, a 20 mL screw top reaction tube was equipped with a magnetic stir bar and charged with tri(trimethylsilyl)silane (3.86 mL, 12.5 mmol, 1.0 equiv), 2-bromopropane (2.35 mL, 25.0 mmol, 2.0 equiv), and Et_2O (4 mL). The reaction tube was capped with a PTFE/silicon septum and irradiated with blue LEDs for 12 hours. After irradiation, the septum was carefully pierced with a needle to allow the slow evolution of gas. Once bubbling subsided, the solution was transferred to a round-bottom flask containing 10% aq NaOH solution (11 mL, 1.1 eq) and stirred for 24 hours under air at room temperature. After this time, the solution was transferred to a separatory funnel, Et_2O (10 mL) was added, and the organic layer was separated. The organic layer was dried with Na_2SO_4, followed by concentration *in vacuo*. The crude silanol sample was then placed on high vacuum for 8 hours to remove solvent and residual silane impurities to yield pure silanol as a clear oil. The physical properties and spectral data were consistent with those reported in literature.[2]

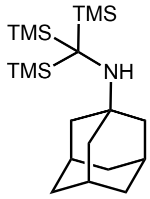

N-(adamantan-1-yl)-1,1,1,3,3,3-hexamethyl-2-(trimethylsilyl)trisilan-2-amine ($(TMS)_3SiNHAdm$): Prepared according to literature precedent reported by MacMillan et al.[3]

Preparation of substrates:

General procedure for the synthesis of **1**, **S1-S4**, **S6-S8**, **S13**, **S15-S18**: To an appropriately sized round-bottom flask equipped with a stir bar was added the halophenol (1 equiv) and K_2CO_3 (2 equiv). The flask was equipped with a nitrogen balloon and DMF was added (0.5 M) and stirred for 15 minutes before adding the allyl bromide (1.1 equiv) via syringe. The reaction was stirred at room temperature and monitored by TLC until full consumption of the halophenol was observed. Upon reaction completion, the reaction was diluted with EtOAc (50 mL) H_2O (50 mL). The reaction mixture was transferred to a separatory funnel and the layers were separated. The organic layer was washed with 1 M LiCl (3 x 50 mL), brine (1 x 50 mL), and dried over Na_2SO_4. The compound was then purified via silica gel chromatography.

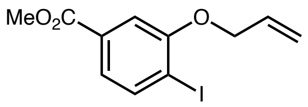

Methyl 3-(allyloxy)-4-iodobenzoate[4] (1): Prepared according to general substrate synthesis procedure using methyl 3-hydroxy-4-iodobenzoate (5 mmol, 1.390 g, 1.0 equiv), K_2CO_3 (10 mmol, 1.382 g, 2.0 equiv), DMF (10 mL), and allyl bromide (5.5 mmol, 0.480 mL, 1.1 equiv). The reaction was purified by silica chromatography (20% EtOAc/hexanes as the eluent) to afford the title compound as a white solid (1.442 g, 91%). The physical properties and spectral data were consistent with the reported values.

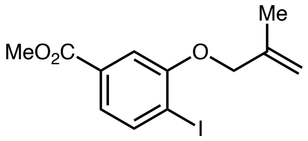

Methyl 4-iodo-3-((2-methylallyl)oxy)benzoate[5] (S1): Prepared according to general substrate synthesis procedure using methyl 3-hydroxy-4-iodobenzoate (5 mmol, 1.390 g, 1.0 equiv), K_2CO_3 (10 mmol, 1.382 g, 2.0 equiv), DMF (10 mL), and 3-bromo-2-methylprop-1-ene (5.5 mmol, 0.555 mL, 1.1 equiv). The reaction was purified by silica chromatography (10% EtOAc/hexanes as the eluent) to afford the title compound as a white solid (1.383 g, 83%). The physical properties and spectral data were consistent with the reported values.

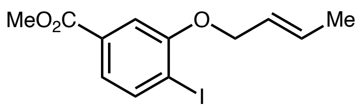

Methyl-3-(but-2-en-1-yloxy)-4-iodobenzoate (S2): Prepared according to general substrate synthesis procedure using methyl 3-hydroxy-4-iodobenzoate (5 mmol, 1.390 g, 1.0 equiv), K_2CO_3 (10 mmol, 1.382 g, 2.0 equiv), DMF (10 mL), and 1-bromobut-2-ene (5.5 mmol, 0.563 mL, 1.1 equiv). The reaction was purified by silica chromatography (5% EtOAc/hexanes as the eluent) to afford the title compound as a white solid (1.442 g, 89%). The mixture was characterized as a mixture of diastereomers with the diastereomeric ratio determined by ^{1}H NMR (d.r. 5.9:1.0, *denotes major diastereomer while #denotes minor diastereomer).

^{1}H NMR (500 MHz, CDCl$_3$) δ 7.85 (d, *J* = 8.1, 1H#), 7.84 (d, *J* = 8.1, 1H*), 7.45 (d, *J* = 1.8 Hz, 1H#), 7.43 (d, *J* = 1.8 Hz, 1H), 7.36 (dd, *J* = 8.1, 1.8 Hz, 1H), 5.98 – 5.89 (m, 1H*), 5.82 – 5.75 (m, 1H#), 5.77 – 5.70 (m, 1H), 4.58 (dt, *J* = 6.1, 1.1 Hz, 2H#), 4.58 (dt, *J* = 5.8 Hz, 1.3 Hz, 2H*), 3.91 (s, 3H#), 3.91 (s, 3H*), 1.80 – 1.76 (m, 3H).

^{13}C NMR (151 MHz, CDCl$_3$) δ 166.8, 166.4, 157.6, 155.2, 139.7, 138.6, 132.5, 131.6, 130.8, 129.5, 125.2, 124.9, 123.4, 123.2, 115.9, 112.8, 93.6, 91.9, 70.1, 65.4, 52.5, 52.5, 18.1, 13.7.

HRMS (APCI pos.) m/z: [M+H] calcd. for $C_{12}H_{14}IO_3$, 332.9982; found 332.9982.

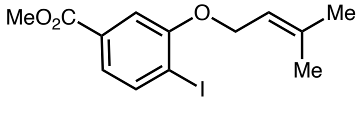

Methyl 4-iodo-3-((3-methylbut-2-en-1-yl)oxy)benzoate (S3): Prepared according to general substrate synthesis procedure using methyl 3-hydroxy-4-iodobenzoate (5 mmol, 1.390 g, 1.0 equiv), K_2CO_3 (10 mmol, 1.382 g, 2.0 equiv), DMF (10 mL), and 1-bromo-3-methylbut-2-ene (5.5 mmol, 0.640 mL, 1.1 equiv). The reaction was purified by silica chromatography (1% EtOAc/hexanes as the eluent) to afford the title compound as a white solid (1.383 g, 83%).
^{1}H NMR (400 MHz, $CDCl_3$) δ 7.84 (d, *J* = 8.1 Hz, 1H), 7.44 (d, *J* = 1.8 Hz, 1H), 7.34 (dd, *J* = 8.1, 1.8 Hz, 1H), 5.54 – 5.46 (m, 1H), 4.64 (d, *J* = 6.6 Hz, 2H), 3.91 (s, 3H), 1.80 (s, 3H), 1.78 (s, 3H).
^{13}C NMR (151 MHz, $CDCl_3$) δ 166.8, 157.7, 139.6, 138.8, 131.6, 123.3, 119.1, 112.9, 93.7, 66.6, 52.5, 26.0, 18.6.
HRMS (APCI pos.) m/z: [M+H] calcd. for $C_{13}H_{16}IO_3$, 347.0139; found 347.0137.

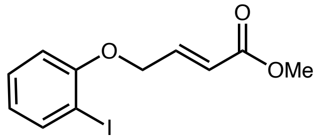

Methyl (*E*)-4-(2-iodophenoxy)but-2-enoate[6] (S4): Prepared according to general substrate synthesis procedure using 2-iodophenol (5 mmol, 1.100 g, 1.0 equiv), K_2CO_3 (10 mmol, 1.382 g, 2.0 equiv), DMF (10 mL) and methyl 4-bromocrotonate (5.5 mmol, 0.647 mL, 1.1 equiv). The reaction was purified by silica gel chromatography (5-15% EtOAc/hexanes as the eluent) to afford the title compound as a white solid. The physical data and spectral properties were consistent with the reported values.

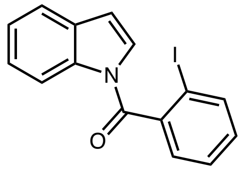

(1*H*-indol-1-yl)(2-iodophenyl)methanone[7] (S5): Prepared according to literature procedure. CDI (1.78 g, 11 mmol) was added to a solution of 2-iodobenzoic acid (2.48 g, 10 mmol in THF (20 mL). After 16 hours, the reaction mixture was washed with water (1x 50 mL), brine (1x 50mL), dried over Na_2SO_4 and concentrated *in vacuo*. To an oven-dried flask was added the acyl imidazole (1.97 g, 6.6 mmol, 1.1 equiv) and the atmosphere was evacuated and backfilled 3x. MeCN (18 mL), indole (703 mg, 6.0 mmol, 1 equiv), and DBU (0.181 mL, 1.2 mmol, 20 mol%) were added and the reaction was stirred for 16 hours. The reaction was quenched with saturated NH_4Cl (aq), and extracted 3x with EtOAc. The combined organic layers were washed with brine and concentrated *in vacuo*. The reaction was purified by silica gel chromatography (10-30% EtOAc/hexanes as the eluent) to afford the title compound as a white amorphous solid. The physical data and spectral properties were consistent with the reported values.

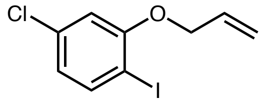

2-(allyloxy)-4-chloro-1-iodobenzene (S6): Prepared according to general substrate synthesis procedure using 5-chloro-2-iodophenol (3 mmol, 0.763 g, 1.0 equiv), K_2CO_3 (6 mmol, 0.829 g, 2.0 equiv), DMF (6 mL), and allyl bromide (3.3 mmol, 0.286 mL, 1.1 equiv). The reaction was purified by elution through a silica plug using EtOAc to afford the title compound as a brown oil (0.795 g, 90%).
^{1}H NMR (600 MHz, $CDCl_3$) δ 7.67 (d, *J* = 8.3 Hz, 1H), 6.78 (d, *J* = 2.2 Hz, 1H), 6.73 (dd, *J* = 8.3, 2.2 Hz, 1H), 6.08 – 6.00 (m, 1H), 5.53 (dq, *J* = 17.3, 1.6 Hz, 1H), 5.34 (dq, *J* = 10.7, 1.6 Hz, 1H), 4.59 (dt, *J* = 4.9, 1.6 Hz, 2H).
^{13}C NMR (101 MHz, $CDCl_3$) δ 157.9, 140.0, 135.2, 132.1, 122.9, 118.2, 113.2, 84.0, 70.0.
HRMS (APCI pos.) m/z: [M+H] calcd. for C_9H_9OClI, 294.9381; found 294.9381.

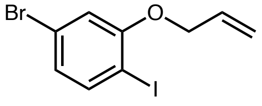

2-(allyloxy)-4-bromo-1-iodobenzene (S7): Prepared according to general substrate synthesis procedure using 5-chloro-2-iodophenol (3 mmol, 0.897 g, 1.0 equiv), K_2CO_3 (6 mmol, 0.829 g, 2.0 equiv), DMF (6 mL), and allyl bromide (3.3 mmol, 0.286 mL, 1.1 equiv). The reaction was purified by elution through a silica plug using EtOAc to afford the title compound as a brown oil (0.950 g, 93%).

^{1}H NMR (600 MHz, CDCl$_3$) δ 7.61 (d, *J* = 8.3 Hz, 1H), 6.92 (d, *J* = 2.0 Hz, 1H), 6.86 (dd, *J* = 8.3, 2.1 Hz, 1H), 6.08 – 6.00 (m, 1H), 5.52 (dq, *J* = 17.3, 1.6 Hz, 1H), 5.34 (dq, *J* = 10.7, 1.6 Hz, 1H), 4.58 (dt, *J* = 4.9, 1.7 Hz, 2H).
^{13}C NMR (101 MHz, CDCl$_3$) δ 158.0, 140.3, 132.0, 125.8, 122.9, 118.2, 116.0, 84.9, 70.0.
HRMS (APCI pos.) m/z: [M+H] calcd. for C_9H_9OBrI, 338.8876; found 338.8876.

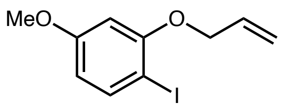

2-(allyloxy)-1-iodo-4-methoxybenzene[8] (S8): Prepared according to general substrate synthesis procedure using 5-chloro-2-iodophenol (3 mmol, 0.897 g, 1.0 equiv), K_2CO_3 (6 mmol, 0.829 g, 2.0 equiv), DMF (6 mL), and allyl bromide (3.3 mmol, 0.286 mL, 1.1 equiv). The reaction was purified by elution through a silica plug using EtOAc to afford the title compound as a colorless oil (0.950 g, 93%). The physical data and spectral properties were consistent with previously reported data.

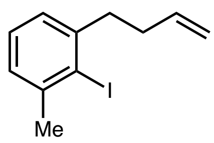

1-(but-3-en-1-yl)-2-iodo-3-methylbenzene (**S9**): To an oven-dried flask was added 1-(bromomethyl)-2-iodobenzene (0.933 g, 3.0 mmol, 1.2 equiv) and dry THF (12 mL). The solution was cooled to 0 °C and allylmagenisum bromide (1.0 M in ether, 2.5 mL, 2.5 mmol, 1.2 equiv) was added dropwise. The reaction was heated to reflux for 2 hours after which TLC indicated complete starting material consumption. The reaction was quenched with saturated NH_4Cl (50 mL) and extracted 3x with EtOAc (3x 50 mL). The combined organic layers were dried with Na_2SO_4 and concentrated *in vacuo*. The reaction was purified by silica gel chromatography (100% pentanes as the eluent) to give the title compound as a colorless oil (0.397 g, 58%).
^{1}H NMR (600 MHz, CDCl$_3$) δ 7.14 (t, *J* = 7.4 Hz, 1H), 7.08 (dd, *J* = 7.4, 1.7 Hz, 1H), 7.01 (dd, *J* = 7.4, 1.8 Hz, 1H), 5.91 (ddt, *J* = 16.9, 10.2, 6.6 Hz, 1H), 5.11–5.07 (m, 1H), 5.04 – 4.99 (m, 1H), 2.91 – 2.83 (m, 2H), 2.48 (s, 3H), 2.39–2.33 (m, 2H).
^{13}C NMR (101 MHz, CDCl$_3$) δ 145.2, 142.5, 137.9, 127.8, 127.5, 126.7, 115.2, 108.1, 41.6, 34.3, 30.2.
HRMS (APCI pos.) m/z: [M+H] calcd. for $C_{11}H_{14}I$ 273.0135; found 273.0135.

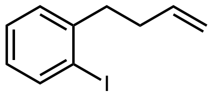

1-(but-3-en-1-yl)-2-iodobenzene[9] (S10): To an oven-dried flask was added 1-(bromomethyl)-2-iodobenzene (1.48 g, 5.0 mmol, 1 equiv) and dry THF (5 mL). The solution was cooled to 0 °C and allylmagenisum bromide (1.0 M in ether, 7.5 mL, 7.5 mmol, 1.5 equiv) was added dropwise. The reaction was heated to reflux for 2 hours after which TLC indicated complete starting material consumption. The reaction was quenched with saturated NH_4Cl (50 mL) and extracted 3x with EtOAc (3x 50 mL). The combined organic layers were dried with Na_2SO_4 and concentrated *in vacuo*. The reaction was purified by silica gel chromatography (1% EtOAc/hexanes as the eluent) to give the title compound as a colorless oil (0.539 g, 42%). The physical data and spectral properties were consistent with previously reported data.

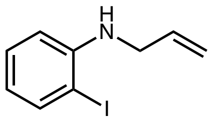

***N*-allyl-2-iodoaniline[10] (S11):** To a 3-neck oven-dried round bottom flask equipped with a magnetic stir bar was added 2-iodoaniline (5 mmol, 1.095 g, 1.0 equiv). The flask was evacuated and backfilled 3x with N_2. Under an N_2 atmosphere, THF (25 mL) was added. This solution was cooled to 0 ºC and a freshly made 0.2 M LDA (5 mmol, 25 mL, 1.0 equiv) was added dropwise to the reaction at 0 ºC. After stirring for 10 minutes at 0 ºC, allyl bromide (6 mmol, 0.518 mL, 1.2 equiv) was added dropwise and the reaction was stirred for 4 hours 0 ºC. After 4 hours, the reaction qas quenched with the slow addition of water (25 mL) at 0 ºC. The reaction was transferred to a separatory funnel and extracted with EtOAc (3x 50 mL). The combined organic layers were washed with brine, dried over

Na_2SO_4, and concentrated *in vacuo*. The reaction was purified by silica gel chromatography (0-2% EtOAc/hexanes as the eluent) to afford the title compound as a brown oil (0.816 g, 63%). The physical data and spectra data were consistent with previously reported values.

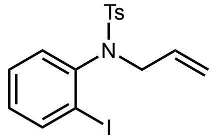

***N*-allyl-*N*-(2-iodophenyl)-4-methylbenzenesulfonamide[11] (S12):** To a 3-neck oven-dried round bottom flask equipped with a magnetic stir bar was added *N*-allyl-2-iodoaniline (**S11**) (2.35 mmol, 0.611 g, 1.0 equiv). The flask was evacuated and backfilled 3x with N_2. Under an N_2 atmosphere, pyridine (6 mL) was added. To this solution was added *p*-Toluenesulfonyl chloride (2.82 mmol, 0.538 g, 1.2 eq) in one portion. The reaction was stirred at room temperature until TLC indicated complete consumption of the aniline starting material. After 3 hours, the crude reaction was concentrated *in vacuo*. The contents of the flask were transferred to a separatory funnel using DCM (25 mL) and water (25 mL). The aqueous layer was extracted with DCM (3x 25 mL) and the combined organic layers were washed with brine, dried over Na_2SO_4, and concentrated *in vacuo*. The reaction was purified by silica gel chromatography (0-6% EtOAc/hexanes) to afford the title compound as a colorless oil (0.384 g, 40%). The physical data and spectral properties were consistent with previously reported values.

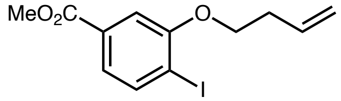

Methyl 3-(but-3-en-1-yloxy)-4-iodobenzoate (S13): Prepared according to general substrate synthesis procedure using methyl 3-hydroxy-4-iodobenzoate (5 mmol, 1.390 g, 1.0 equiv), K_2CO_3 (10 mmol, 1.382 g, 2.0 equiv), DMF (10 mL), and 4-bromobut-1-ene (5.5 mmol, 0.560 mL, 1.1 equiv). The reaction was purified by silica chromatography (0-5% EtOAc/hexanes as eluent) to afford the title compound as a clear and colorless oil (1.383 g, 44%).
^{1}H NMR (400 MHz, $CDCl_3$) δ 7.84 (d, *J* = 8.1 Hz, 1H), 7.41 (s, 1H), 7.35 (d, *J* = 8.1 Hz, 1H), 6.03 – 5.91 (m, 1H), 5.21 (dq, *J* = 17.2, 1.6 Hz, 1H), 5.17 – 5.10 (m, 1H), 4.12 (t, *J* = 6.6 Hz, 2H), 3.90 (s, 3H), 2.61 (q, *J* = 6.6 Hz, 2H).
^{13}C NMR (101 MHz, $CDCl_3$) δ 166.7, 157.6, 139.6, 134.2, 131.6, 123.4, 117.6, 112.3, 93.5, 68.9, 52.5, 33.6.
HRMS (APCI pos.) m/z: [M+H] calcd. for $C_{12}H_{14}O_3I$, 332.9982; found 332.9980.

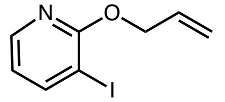

2-(allyloxy)-3-iodopyridine (S14): An oven-dried flask was evacuated and backfilled 3x with N_2. Under a stream of N_2 was added 60% NaH (0.399 g, 10 mmol, 2 equiv) and dry DMF (20 mL). The solution was cooled to 0 °C and allyl alcohol (0.680 mL, 10 mmol, 2 equiv) was added dropwise. The reaction was warmed to room temperature and stirred for 30 minutes. After this time, 2-fluoro-3-iodopyridine was added in one portion. The reaction was stirred at room temperature for 16 hours. The reaction was quenched by dropwise addition of H_2O until bubbling subsided then diluted with 50 mL H_2O and 50 mL EtOAc. The mixture was washed 3x w/ 1M LiCl (3x 50 mL), then brine (1x 50 mL). The organic layer was separated and dried with Na_2SO_4 then concentrated *in vacuo*. The reaction was purified by silica gel chromatography (5% EtOAc/Hexanes as the eluent) to afford the title compound as a brown oil (1.105 g, 85% yield).
^{1}H NMR (400 MHz, $CDCl_3$) δ 8.09 (d, *J* = 4.9 Hz, 1H), 8.03 (d, *J* = 7.5 Hz, 1H), 6.65 (t, *J* = 6.8 Hz, 1H), 6.18 – 5.97 (m, 1H), 5.47 (d, *J* = 17.2 Hz, 1H), 5.27 (d, *J* = 10.6 Hz, 1H), 4.88 (d, *J* = 5.1 Hz, 2H).
^{13}C NMR (101 MHz, $CDCl_3$) δ 161.4, 148.3, 146.6, 133.0, 118.4, 117.3, 80.2, 67.7.
HRMS (APCI pos.) m/z: [M+H] calcd. for C_8H_9NOI, 261.9723; found 261.9721.

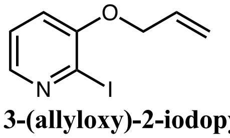

3-(allyloxy)-2-iodopyridine[12] (S15): Prepared according to general substrate synthesis procedure using 2-iodopyridin-3-ol (10 mmol, 2.210 g, 1.0 equiv), K_2CO_3 (10 mmol, 2.764 g, 2.0 equiv), DMF (20 mL), and allyl bromide (11 mmol, 1.038 mL, 1.2 equiv). The reaction was purified by silica chromatography (20% EtOAc/hexanes

as eluent) to afford the title compound as a clear and brown oil (0.697 g, 27%). The physical data and spectral properties were consistent with previously reported values.

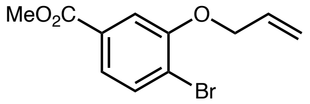

Methyl 3-(allyloxy)-4-bromobenzoate (S16): Prepared according to general substrate synthesis procedure using methyl 3-hydroxy-4-iodobenzoate (1.2 mmol, 0.227 g, 1.0 equiv), K_2CO_3 (2.4 mmol, 0.332 g, 2.0 equiv), DMF (2.4 mL), and allyl bromide (1.44 mmol, 0.124 mL, 1.2 equiv). The reaction was purified by a silica plug using 100% ether as the eluent to afford the title compound as a white solid (0.304 g, 93%).

^{1}H NMR (600 MHz, $CDCl_3$) δ 7.61 (d, *J* = 8.2 Hz, 1H), 7.54 (d, *J* = 1.8 Hz, 1H), 7.51 (dd, *J* = 8.2, 1.8 Hz, 1H), 6.12 – 6.03 (m, 1H), 5.51 (dq, *J* = 17.2, 1.7 Hz, 1H), 5.34 (dq, *J* = 10.6, 1.7 Hz, 1H), 4.67 (dt, *J* = 5.0, 1.7 Hz, 2H), 3.91 (s, 3H).

^{13}C NMR (151 MHz, $CDCl_3$) δ 166.5, 155.1, 133.5, 132.2, 130.6, 123.1, 118.3, 118.2, 114.0, 69.9, 52.5.

HRMS (APCI pos.) m/z: [M+H] calcd. for $C_{11}H_{12}O_3Br$, 270.9964; found 270.9962.

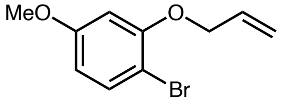

2-(allyloxy)-1-bromo-4-methoxybenzene[13] (S17): Prepared according to general substrate synthesis procedure using 2-bromo-5-methoxyphenol (5 mmol, 1.1015 g, 1.0 equiv), K_2CO_3 (10 mmol, 1.382 g, 2.0 equiv), DMF (10 mL), and allyl bromide (5.5 mmol, 0.480 mL, 1.1 equiv). The reaction was purified by a silica chromatography (10% EtOAc/hex as the eluent) to afford the title compound as a colorless oil (1.114 g, 92%). The physical data and spectral properties are consistent with previously reported values.

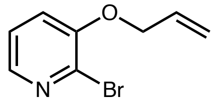

3-(allyloxy)-2-bromopyridine[14] (S18): Prepared according to general substrate synthesis procedure using 2-bromopyridin-3-ol (5 mmol, 1.1015 g, 1.0 equiv), K_2CO_3 (10 mmol, 1.382 g, 2.0 equiv), DMF (10 mL), and allyl bromide (7.5 mmol, 0.650 mL, 1.5 equiv). The reaction was purified by a silica chromatography (0-30% EtOAc/hex as the eluent) to afford the title compound as a yellow oil (0.89 g, 85%). The physical data and spectral properties are consistent with previously reported values.

V. Preparation of Products in Table 2

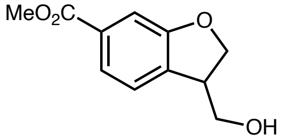

Methyl 3-(hydroxymethyl)-2,3-dihydrobenzofuran-6-carboxylate (2): Prepared according to general procedure A using methyl 4-iodo-3-(2-propen-1-yloxy)benzoate (**1**) (1.0 mmol, 0.318 g, 1.0 equiv), sodium carbonate (2.0 mmol, 0.212 g, 2.0 equiv), Cl-4CzIPN (0.05 mmol, 0.054 g, 5 mol%), tris(trimethylsilyl)silanol (1.5 mmol, 0.46 mL, 1.5 equiv) in MeOH (10 mL). The reaction was purified by silica chromatography (40-50% EtOAc/hex as the eluent) to afford the title compound as a pale-yellow oil (0.171 g, 82% yield).

^{1}H NMR (600 MHz, $CDCl_3$) δ 7.57 (dd, *J* = 7.7, 1.5 Hz, 1H), 7.41 (d, *J* = 1.5 Hz, 1H), 7.26 (d, *J* = 7.7 Hz, 1H), 4.68 (t, *J* = 9.1 Hz, 1H), 4.51 (dd, *J* = 9.1, 5.6 Hz, 1H), 3.88 (s, 3H), 3.83 (dd, *J* = 10.7, 5.8 Hz, 1H), 3.79 (dd, *J* = 10.7, 6.8 Hz, 1H), 3.66 (m, 1H), 1.92 (broad s, 1H).

^{13}C NMR (101 MHz, $CDCl_3$) δ 167.1, 160.8, 133.1, 131.2, 124.5, 122.6, 110.6, 74.6, 64.7, 52.3, 44.6.

HRMS (APCI pos.) m/z: [M+H] calcd. for $C_{11}H_{13}O_4$, 209.0808; found 209.0809.

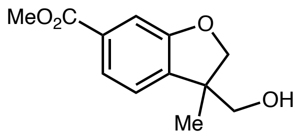

Methyl 3-(hydroxymethyl)-3-methyl-2,3-dihydrobenzofuran-6-carboxylate (4): Prepared according to general procedure A using methyl 4-iodo-3-((2-methylallyl)oxy)benzoate (**S1**) (0.5 mmol, 0.166 g, 1.0 equiv), sodium carbonate (1.0 mmol, 0.106 g, 2.0 equiv), Cl-4CzIPN (0.025 mmol, 0.027 g, 5 mol%), tris(trimethylsilyl)silanol (0.75 mmol, 0.23 mL, 1.5 equiv) in MeOH (5 mL). The reaction was purified by silica chromatography (20-30% EtOAc/hex as the eluent) to afford the title compound as a pale-yellow oil (0.062 g, 56% yield).

^{1}H NMR (400 MHz, CDCl$_3$) δ 7.62 (dd, *J* = 7.7, 1.4 Hz, 1H), 7.44 (d, *J* = 1.4 Hz, 1H), 7.17 (d, *J* = 7.7Hz, 1H), 4.62 (d, *J* = 8.9 Hz, 1H), 4.24 (d, *J* = 8.9 Hz, 1H), 3.89 (s, 3H), 3.70 (d, *J* = 10.7 Hz, 1H), 3.61 (d, *J* = 10.7 Hz, 1H), 1.57 (broad s, 1H), 1.39 (s, 3H).

^{13}C NMR (101 MHz, CDCl$_3$) δ 167.0, 160.5, 137.6, 131.3, 123.0, 122.8, 110.9, 80.6, 68.9, 52.3, 47.9, 21.9.

HRMS (APCI pos.) m/z: [M+H] calcd. for $C_{12}H_{15}O_4$, 223.0965; found 223.0957.

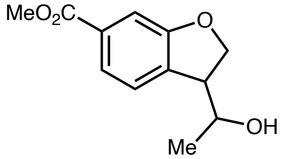

Methyl 3-(1-hydroxyethyl)-2,3-dihydrobenzofuran-6-carboxylate (5): Prepared according to general procedure A using methyl-3-(but-2-en-1-yloxy)-4-iodobenzoate (**S2**) (0.5 mmol, 0.145 g, 1.0 equiv), sodium carbonate (1.0 mmol, 0.106 g, 2.0 equiv), Cl-4CzIPN (0.025 mmol, 0.027 g, 5 mol%), tris(trimethylsilyl)silanol (0.75 mmol, 0.23 mL, 1.5 equiv) in MeOH (5 mL). The reaction was purified by silica chromatography (25-35% EtOAc/hex as the eluent) to afford the title compound as a pale-yellow oil (0.073 g, 66% yield). The compound was characterized as a mixture of diastereomers with the diastereomeric ratio determined by ^{1}H NMR (1.0:1.0 d.r., *denotes diastereomeric peaks).

^{1}H NMR (400 MHz, CDCl$_3$) δ 7.56 (d, *J* = 7.8 Hz, 1H), 7.39 (s, 1H), 7.33 (d, *J* = 7.8 Hz, 1H*), 7.26 (d, *J* = 7.7 Hz, 1H*), 4.64 (dd, *J* = 9.3, 5.8 Hz, 1H*), 4.59 (t, *J* = 9.3 Hz, 1H), 4.49 (dd, *J* = 9.3, 5.1 Hz, 1H*), 4.06 (p, *J* = 5.5 Hz, 1H*), 3.96 (p, *J* = 6.3 Hz, 1H*), 3.87 (s, 3H), 3.55 – 3.45 (m, 1H), 2.31 (broad s, 1H), 1.22 (d, *J* = 6.3 Hz, 3H*), 1.17 (d, *J* = 6.3 Hz, 3H*).

^{13}C NMR (101 MHz, CDCl$_3$) δ 167.1, 167.0, 161.1, 160.8, 133.2, 132.6, 131.3, 131.2, 125.4, 124.8, 122.5, 110.6, 110.6, 73.6, 73.1, 69.9, 68.89, 52.3, 49.4, 49.2, 20.6, 20.2.

HRMS (APCI pos.) m/z: [M+H] calcd. for $C_{12}H_{15}O_4$, 223.0965; found 223.0964.

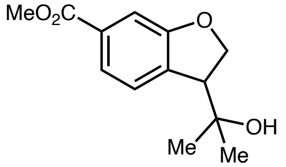

Methyl 3-(2-hydroxypropan-2-yl)-2,3-dihydrobenzofuran-6-carboxylate (6): Prepared according to general procedure A using methyl 4-iodo-3-((3-methylbut-2-en-1-yl)oxy)benzoate (**S3**) (0.5 mmol, 0.173 g, 1.0 equiv), sodium carbonate (1.0 mmol, 0.106 g, 2.0 equiv), Cl-4CzIPN (0.025 mmol, 0.027 g, 5 mol%), tris(trimethylsilyl)silanol (0.75 mmol, 0.23 mL, 1.5 equiv) in MeOH (5 mL). The reaction was purified by silica chromatography (20% EtOAc/hex as the eluent) to afford the title compound as a pale-yellow oil (0.118 g, 86% yield).

^{1}H NMR (400 MHz, CDCl$_3$) δ 7.58 (dd, *J* = 7.8, 1.4 Hz, 1H), 7.41 (d, *J* = 1.4 Hz, 1H), 7.37 (d, *J* = 7.8 Hz, 1H), 4.61 (dd, *J* = 9.6, 5.3 Hz 1H), 4.57 (dd, *J* = 9.6, 9.5 Hz 1H), 3.89 (s, 3H), 3.47 (dd, *J* = 8.5, 5.3 Hz, 1H), 1.62 (broad s, 1H), 1.22 (s, 3H), 1.21 (s, 3H).

^{13}C NMR (101 MHz, CDCl$_3$) δ 167.0, 161.0, 132.9, 131.1, 125.8, 122.1, 110.4, 73.9, 72.6, 53.2, 52.2, 26.8, 26.6.

HRMS (APCI pos.) m/z: [M+H] calcd. for $C_{13}H_{17}O_4$, 237.1121; found 237.1121.

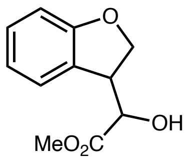

Methyl 2-(2,3-dihydrobenzofuran-3-yl)-2-hydroxyacetate (7): Prepared according to general procedure A using methyl (*E*)-4-(2-iodophenoxy)but-2-enoate (**S4**) (0.5 mmol, 0.159 g, 1.0 equiv), sodium carbonate (1.0 mmol, 0.106 g, 2.0 equiv), Cl-4CzIPN (0.025 mmol, 0.027 g, 5 mol%), tris(trimethylsilyl)silanol (0.75 mmol, 0.23 mL, 1.5 equiv) in MeOH (5 mL). The reaction was purified by silica chromatography (10-20% EtOAc/hex as the eluent) to afford the title compound as a pale-yellow oil (0.075 g, 72% yield). The compound was characterized as a mixture of diastereomers where the diastereomeric ratio was determined by ^{1}H NMR (1.5:1.0 d.r., *denotes major diastereomer, # denotes minor diastereomer).
^{1}H NMR (400 MHz, $CDCl_3$) δ 7.22 (d, *J* = 7.4 Hz, 1H), 7.17 (t, *J* = 7.5 Hz, 1H*), 7.06 (d, *J* = 7.5 Hz, 1H#) 6.88 (dt, *J* = 7.5, 1.0 Hz, 1H*), 6.85 (dt, *J* = 7.5, 1.0 Hz, 1H#), 6.81 (d, *J* = 8.0, 1H), 4.67 – 4.50 (m, 2H), 4.46 (d, *J* = 4.0 Hz, 1H*), 4.34 (d, *J* = 4.5 Hz, 1H#), 3.92 – 3.85 (m, 1H), 3.84 (s, 3H#), 3.78 (s, 3H*), 2.89 (broad s, 1H*), 2,73 (broad s, 1H#).
^{13}C NMR (101 MHz, $CDCl_3$) δ 173.8, 173.6, 161.1, 160.7, 129.5, 129.4, 125.7, 125.3, 124.8, 124.6, 120.7, 120.6, 109.9, 73.4, 72.2, 72.2, 71.8, 52.9, 52.9, 46.3, 46.0, 29.8.
HRMS (APCI pos.) m/z: [M+H] calcd. for $C_{11}H_{13}O_4$, 209.0808; found 209.0808.

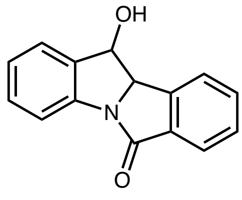

11-hydroxy-10b,11-dihydro-6*H*-isoindolo[2,1-*a*]indol-6-one[15] (8): Prepared according to general procedure A with the exception that the reaction was run open to air rather than sparged with O_2 using (1*H*-indol-1-yl)(2-iodophenyl)methanone (**S5**) (0.5 mmol, 0.174 g, 1.0 equiv), sodium carbonate (1.0 mmol, 0.106 g, 2.0 equiv), Cl-4CzIPN (0.025 mmol, 0.027 g, 5 mol%), tris(trimethylsilyl)silanol (0.75 mmol, 0.23 mL, 1.5 equiv) in 5% H_2O in MeCN (5 mL). The reaction was obtained from the crude NMR (1.7:1.0 d.r.) prior to purifying by silica chromatography (20-50% EtOAc/hex as the eluent) to afford the title compound as a pale-yellow solid (0.079 g, 67% yield). The physical data and spectral properties are consistent with previously reported data.

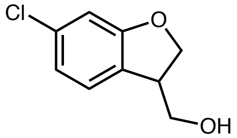

(6-chloro-2,3-dihydrobenzofuran-3-yl)methanol (9): Prepared according to general procedure A using 2-(allyloxy)-4-chloro-1-iodobenzene (**S6**) (0.5 mmol, 0.147 g, 1.0 equiv), sodium carbonate (1.0 mmol, 0.106 g, 2.0 equiv), Cl-4CzIPN (0.025 mmol, 0.027 g, 5 mol%), tris(trimethylsilyl)silanol (0.75 mmol, 0.23 mL, 1.5 equiv) in MeOH (5 mL). The reaction was purified by silica chromatography (0-30% EtOAc/hex as the eluent) to afford the title compound as a colorless oil (0.060 g, 65% yield).
^{1}H NMR (600 MHz, $CDCl_3$) δ 7.12 (d, *J* = 7.9 Hz, 1H), 6.84 (dd, *J* = 7.9, 1.8 Hz 1H), 6.8 (d, *J* = 1.8 Hz, 1H), 4.71 – 4.61 (t, *J* = 9.1 Hz, 1H), 4.50 (dd, *J* = 9.1, 5.4 Hz, 1H), 3.82 – 3.74 (m, 2H), 3.65 – 3.56 (m, 1H), 1.58 (broad s, 1H).
^{13}C NMR (101 MHz, $CDCl_3$) δ 161.5, 134.4, 126.1, 125.3, 120.7, 110.7, 75.0, 64.9, 44.2.
HRMS (APCI pos.) m/z: [M+H] calcd. for $C_9H_{10}O_2Cl$, 185.0364; found 185.0363.

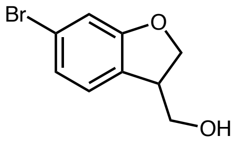

(6-bromo-2,3-dihydrobenzofuran-3-yl)methanol (10): Prepared according to general procedure A using 2-(allyloxy)-4-bromo-1-iodobenzene (**S7**) (0.5 mmol, 0.169 g, 1.0 equiv), sodium carbonate (1.0 mmol, 0.106 g, 2.0 equiv), Cl-4CzIPN (0.025 mmol, 0.027 g, 5 mol%), tris(trimethylsilyl)silanol (0.5 mmol, 0.15 mL, 1.0 equiv) in MeOH (5 mL). The reaction was purified by silica chromatography (0-30% EtOAc/hex as the eluent) to afford the title compound as a colorless oil (0.076 g, 66% yield).
^{1}H NMR (600 MHz, $CDCl_3$) δ 7.07 (d, *J* = 7.8 Hz, 1H), 6.99 (dd, *J* = 7.8, 1.7 Hz, 1H), 6.96 (d, *J* = 1.7 Hz, 1H), 4.65 (t, *J* = 9.1 Hz, 1H), 4.49 (dd, *J* = 9.1, 5.4 Hz, 1H), 3.82 – 3.72 (m, 2H), 3.61 – 3.56 (m, 1H).
^{13}C NMR (101 MHz, $CDCl_3$) δ 161.7, 126.7, 125.8, 123.6, 122.1, 113.5, 74.9, 64.8, 44.2.
HRMS (APCI pos.) m/z: [M+H] calcd. for $C_9H_{10}O_2Br$, 228.9859; found 228.9859.

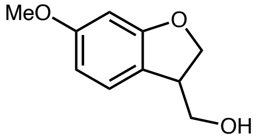

(6-methoxy-2,3-dihydrobenzofuran-3-yl)methanol (11): Prepared according to general procedure A using 2-(allyloxy)-1-iodo-4-methoxybenzene (**S8**) (0.5 mmol, 0.145 g, 1.0 equiv), sodium carbonate (1.0 mmol, 0.106 g, 2.0 equiv), Cl-4CzIPN (0.025 mmol, 0.027 g, 5 mol%), tris(trimethylsilyl)silanol (0.75 mmol, 0.23 mL, 1.5 equiv) in MeOH (5 mL). The reaction was purified by silica chromatography (0-30% EtOAc/hex as the eluent) to afford the title compound as a colorless oil (0.075 g, 83% yield).
^{1}H NMR (600 MHz, $CDCl_3$) δ 7.09 (d, *J* = 8.1 Hz, 1H), 6.42 (dd, *J* = 8.1, 2.3 Hz, 1H), 6.40 (d, *J* = 2.3 Hz, 1H), 4.65 (t, *J* = 9.0 Hz, 1H), 4.49 (dd, *J* = 9.0, 5.3 Hz, 1H), 3.79 – 3.73 (m, 2H), 3.77 (s, 3H), 3.60 – 3.54 (m, 1H), 1.61 (broad s, 1H)
^{13}C NMR (101 MHz, $CDCl_3$) δ 162.0, 161.1, 124.8, 119.1, 106.3, 96.5, 75.1, 65.2, 55.6, 44.1.
HRMS (APCI pos.) m/z: [M+H] calcd. for $C_{10}H_{13}O_3$, 181.0859; found 181.0859.

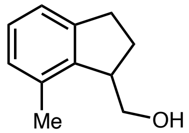

(7-methyl-2,3-dihydro-1*H*-inden-1-yl)methanol (12): Prepared according to general procedure A using 1-(but-3-en-1-yl)-2-iodo-3-methylbenzene (**S9**) (0.5 mmol, 0.136 g, 1.0 equiv), sodium carbonate (1.0 mmol, 0.106 g, 2.0 equiv), Cl-4CzIPN (0.025 mmol, 0.027 g, 5 mol%), tris(trimethylsilyl)silanol (0.75 mmol, 0.23 mL, 1.5 equiv) in MeOH (5 mL). The reaction was purified by silica chromatography (5-20% EtOAc/hex as the eluent) to afford the title compound as a pale-yellow oil (0.0707 g, 87% yield).
^{1}H NMR (400 MHz, $CDCl_3$) δ 7.14–7.04 (m, 2H), 6.97 (d, *J* = 6.8 Hz, 1H), 3.78 (dd, *J* = 10.7, 4.4 Hz, 1H), 3.61 (dd, *J* = 10.7, 7.9 Hz, 1H), 3.45–3.37 (m, 1H), 3.05 (dt, *J* = 16.1, 9.2 Hz, 1H), 2.92 – 2.78 (m, 1H), 2.34 (s, 3H), 2.28 – 2.12 (m, 2H), 1.45 (broad s, 1H).
^{13}C NMR (101 MHz, $CDCl_3$) δ 144.9, 141.9, 134.4, 127.9, 127.4, 122.3, 64.6, 47.0, 31.4, 28.5, 19.1.
HRMS (APCI pos.) m/z: [M+H] calcd. for $C_{11}H_{15}O$, 163.1117; found 163.1117.

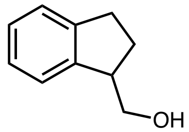

(2,3-dihydro-1*H*-inden-1-yl)methanol[16] (13): Prepared according to general procedure A using methyl 1-(but-3-en-1-yl)-2-iodobenzene (**S10**) (0.5 mmol, 0.129 g, 1.0 equiv), sodium carbonate (1.0 mmol, 0.106 g, 2.0 equiv), Cl-4CzIPN (0.025 mmol, 0.027 g, 5 mol%), tris(trimethylsilyl)silanol (0.75 mmol, 0.23 mL, 1.5 equiv) in MeOH (5 mL). The reaction was purified by silica chromatography (20% EtOAc/hex as the eluent) to afford the title compound as a pale-yellow oil (0.0447 g, 60% yield). The physical data and spectral properties were consistent with previously reported data.

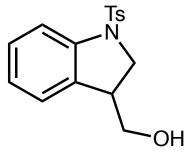

(1-tosylindolin-3-yl)methanol (14): Prepared according to general procedure A using methyl *N*-allyl-*N*-(2-iodophenyl)-4-methylbenzenesulfonamide (**S12**) (0.5 mmol, 0.207 g, 1.0 equiv), sodium carbonate (1.0 mmol, 0.106 g, 2.0 equiv), Cl-4CzIPN (0.025 mmol, 0.027 g, 5 mol%), tris(trimethylsilyl)silanol (0.75 mmol, 0.23 mL, 1.5 equiv) in MeOH (5 mL). The reaction was purified by silica chromatography (30-40% EtOAc/hex as the eluent) to afford the title compound as a pale-yellow oil (0.152 g, 66% yield).
^{1}H NMR (400 MHz, $CDCl_3$) δ 7.69 (d, *J* = 8.3 Hz, 2H), 7.66 (d, *J* = 8.1 Hz, 1H), 7.26 – 7.20 (m, *J* = 8.0, 5.9 Hz, 3H), 7.12 (d, *J* = 7.4 Hz, 1H), 6.99 (t, *J* = 7.4 Hz, 1H), 3.97 (dd, *J* = 10.9, 9.1 Hz, 1H), 3.88 (dd, *J* = 10.9, 5.1 Hz, 1H), 3.59 (dd, *J* = 10.6, 5.5 Hz, 1H), 3.44 (dd, *J* = 10.6, 7.2 Hz, 1H), 3.37 – 3.29 (m, 1H), 2.37 (s, 3H), 1.50 (broad s, 1H).
^{13}C NMR (101 MHz, $CDCl_3$) δ 144.3, 142.5, 134.0, 131.8, 129.8, 128.8, 127.5, 125.0, 123.8, 115.1, 64.9, 52.9, 42.69, 21.7.
HRMS (APCI pos.) m/z: [M+H] calcd. for $C_{16}H_{18}O_3NS$, 304.1002; found 304.1001.

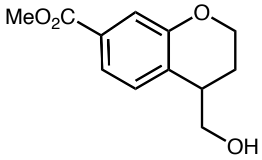

Methyl 4-(hydroxymethyl)chromane-7-carboxylate (15): Prepared according to general procedure A using methyl 3-(but-3-en-1-yloxy)-4-iodobenzoate (**S13**) (0.5 mmol, 0.166 g, 1.0 equiv), sodium carbonate (1.0 mmol, 0.106 g, 2.0 equiv), Cl-4CzIPN (0.025 mmol, 0.027 g, 5 mol%), tris(trimethylsilyl)silanol (0.75 mmol, 0.23 mL, 1.5 equiv) in MeOH (5 mL). The reaction was purified by silica chromatography (40-50% EtOAc/hex as the eluent) to afford the title compound as a pale-yellow oil (0.076 g, 68% yield).
^{1}H NMR (400 MHz, $CDCl_3$) δ 7.52 (dd, *J* = 8.0, 1.7 Hz, 1H), 7.48 (d, *J* = 1.7 Hz, 1H), 7.24 (d, *J* = 8.0 Hz, 1H), 4.25–4.18 (m, 2H), 3.91 (dd, *J* = 11.0, 5.2 Hz, 1H), 3.88 (s, 3H), 3.81 (dd, *J* = 11.0, 7.9 Hz, 1H), 3.09–3.00 (m, 1H), 2.16 – 2.02 (m, 2H), 1.65 (broad s, 1H).
^{13}C NMR (101 MHz, $CDCl_3$) δ 167.0, 155.4, 130.0, 129.4, 127.6, 121.3, 118.5, 66.4, 63.7, 52.2, 36.5, 24.3.
HRMS (APCI pos.) m/z: [M+H] calcd. for $C_{12}H_{15}O_4$, 223.0965; found 223.0965.

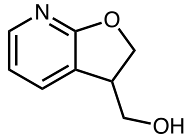

(2,3-dihydrofuro[2,3-*b*]pyridin-3-yl)methanol (16): Prepared according to general procedure A using 2-(allyloxy)-3-iodopyridine (**S14**) (0.5 mmol, 0.131 g, 1.0 equiv), sodium carbonate (1.0 mmol, 0.106 g, 2.0 equiv), Cl-4CzIPN (0.025 mmol, 0.027 g, 5 mol%), tris(trimethylsilyl)silanol (0.75 mmol, 0.23 mL, 1.5 equiv) in MeOH (5 mL). The reaction was purified by silica chromatography (0-2% MeOH/DCM as the eluent) to afford the title compound as a pale-yellow oil (0.034 g, 45% yield).
^{1}H NMR (400 MHz, $CDCl_3$) δ 8.01 (d, *J* = 5.1 Hz, 1H), 7.55 (ddd, *J* = 7.2, 1.8, 1.1 Hz, 1H), 6.80 (dd, *J* = 7.2, 5.1 Hz,1H), 4.68 (t, *J* = 9.3 Hz, 1H), 4.48 (dd, *J* = 9.3, 5.5 Hz, 1H), 3.83 (d, *J* = 6.6 Hz, 2H), 3.75 – 3.63 (m, 1H), 2.04 (s, 1H).
^{13}C NMR (101 MHz, $CDCl_3$) δ 168.7, 147.0, 134.2, 121.1, 116.7, 72.3, 64.4, 43.0.
HRMS (APCI pos.) m/z: [M+H] calcd. for $C_8H_{10}O_2N$, 152.0706; found 152.0710.

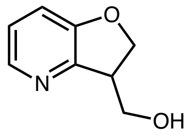

(2,3-dihydrofuro[3,2-*b*]pyridin-3-yl)methanol (17): Prepared according to general procedure A using 3-(allyloxy)-2-iodopyridine (**S15**) (0.5 mmol, 0.131 g, 1.0 equiv), sodium carbonate (1.0 mmol, 0.106 g, 2.0 equiv), Cl-4CzIPN (0.025 mmol, 0.027 g, 5 mol%), tris(trimethylsilyl)silanol (0.75 mmol, 0.23 mL, 1.5 equiv) in MeOH (5 mL). The reaction was purified by silica chromatography (3-6% MeOH/DCM as the eluent) to afford the title compound as an off-white solid (0.051 g, 68% yield).
^{1}H NMR (600 MHz, $CDCl_3$) δ 7.95 (t, *J* = 3.1 Hz, 1H), 7.03 – 7.01 (m, 2H), 4.74 (t, *J* = 9.4 Hz, 1H), 4.49 (dd, *J* = 9.4, 6.9 Hz, 1H), 3.95 – 3.90 (m, 2H), 3.70 – 3.64 (m, 1H).
^{13}C NMR (101 MHz, $CDCl_3$) δ 154.3, 151.6, 141.4, 123.2, 116.3, 73.7, 63.8, 44.4.
HRMS (APCI pos.) m/z: [M+H] calcd. for $C_8H_{10}O_2N$, 152.0706; found 152.0702.

VI. Preparation of Products in Table 4

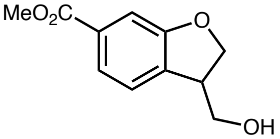

Methyl 3-(hydroxymethyl)-2,3-dihydrobenzofuran-6-carboxylate (2): Prepared according to general procedure B using methyl 3-(allyloxy)-4-bromobenzoate (**S17**) (0.1 mmol, 27.1 mg, 1.0 equiv), sodium carbonate (0.2 mmol, 21.2 mg, 2.0 equiv), (Ir[dF(CF_3)ppy]$_2$(5,5'-dCF_3bpy))PF_6 (0.001 mmol, 1.1 mg, 1 mol%), $(TMS)_3$SiNHAdm (0.3 mmol, 119 mg, 3.0 equiv), and TBAI (0.25 mmol, 9.2 mg, 25 mol%) in 5% H_2O in MeCN (5 mL). The reaction was purified by preparative TLC (50% EtOAc/Hex) to afford the title compound as a pale-yellow oil (16.2 mg, 78% yield). The physical data and spectral properties match previously reported characterization data for **2**.

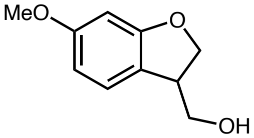

(6-methoxy-2,3-dihydrobenzofuran-3-yl)methanol (11): Prepared according to general procedure B using methyl 3-(allyloxy)-4-bromobenzoate (**S8**) (0.1 mmol, 24.3 mg, 1.0 equiv), sodium carbonate (0.2 mmol, 21.2 mg, 2.0 equiv), (Ir[dF(CF_3)ppy]$_2$(5,5'-dCF_3bpy))PF_6 (0.001 mmol, 1.1 mg, 1 mol%), $(TMS)_3$SiNHAdm (0.3 mmol, 119 mg, 3.0 equiv), and TBAI (0.25 mmol, 9.2 mg, 25 mol%) in 5% H_2O in MeCN (5 mL). The reaction was purified by preparative TLC (30% EtOAc/Hex) to afford the title compound as a pale-yellow oil (14.7 mg, 82% yield). The physical data and spectral properties match previously reported characterization data for **11**.

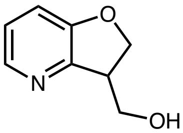

(2,3-dihydrofuro[3,2-*b*]pyridin-3-yl)methanol (17): Prepared according to general procedure B using methyl 3-(allyloxy)-4-bromobenzoate (**S18**) (0.1 mmol, 21.2 mg, 1.0 equiv), sodium carbonate (0.2 mmol, 21.2 mg, 2.0 equiv), (Ir[dF(CF_3)ppy]$_2$(5,5'-dCF_3bpy))PF_6 (0.001 mmol, 1.1 mg, 1 mol%), $(TMS)_3$SiNHAdm (0.3 mmol, 119 mg, 3.0 equiv), and TBAI (0.25 mmol, 9.2 mg, 25 mol%) in 5% H_2O in MeCN (5 mL). The reaction was purified by preparative TLC (80% EtOAc/Hex) to afford the title compound as an off-white solid (10.1 mg, 67% yield). The physical data and spectral properties match previously reported characterization data for **17**.

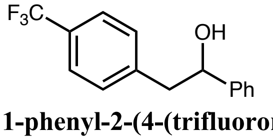

1-phenyl-2-(4-(trifluoromethyl)phenyl)ethan-1-ol[17] (22): Prepared according to general procedure B using 1-bromo-4-(trifluoromethyl)benzene (0.1 mmol, 22.5 mg, 1.0 equiv), sodium carbonate (0.2 mmol, 21.2 mg, 2.0 equiv), (Ir[dF(CF_3)ppy]$_2$(5,5'-dCF_3bpy))PF_6 (0.001 mmol, 1.1 mg, 1 mol%), $(TMS)_3$SiNHAdm (0.3 mmol, 119 mg, 3.0

equiv), TBAI (0.25 mmol, 9.2 mg, 25 mol%), and styrene (0.3 mmol, 0.034 mL, 3 equiv) in 5% H_2O in MeCN (5 mL). The reaction was purified by preparative TLC (10% EtOAc/Hex) to afford the title compound as a yellow solid (11.3 mg, 42% yield). The physical data and spectral properties match previously reported values.
^{1}H NMR (500 MHz, $CDCl_3$) δ 7.54 (d, *J* = 7.9 Hz, 2H), 7.40 – 7.24 (m, 7H), 4.93 (t, *J* = 6.6 Hz), 3.14 – 3.02 (m, 2H), 1.91 (broad s, 1H).

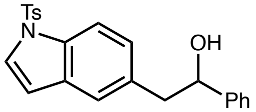

1-phenyl-2-(1-tosyl-1*H*-indol-5-yl)ethan-1-ol[18] (23): Prepared according to general procedure B using 5-bromo-1-tosyl-1*H*-indole (0.1 mmol, 35.0 mg, 1.0 equiv), sodium carbonate (0.2 mmol, 21.2 mg, 2.0 equiv), (Ir[dF(CF_3)ppy]$_2$(5,5'-dCF_3bpy))PF_6 (0.001 mmol, 1.1 mg, 1 mol%), $(TMS)_3$SiNHAdm (0.3 mmol, 119 mg, 3.0 equiv), TBAI (0.25 mmol, 9.2 mg, 25 mol%), and styrene (0.3 mmol, 0.034 mL, 3 equiv) in 5% H_2O in MeCN (5 mL). The reaction was purified by preparative TLC (20% EtOAc/Hex) to afford the title compound as a yellow solid (18.7 mg, 48% yield). The physical data and spectral properties match previously reported values.
^{1}H NMR (600 MHz, $CDCl_3$) δ 7.90 (d, *J* = 8.6 Hz, 1H), 7.76 (d, *J* = 8.6 Hz, 2H), 7.55 (d, *J* = 3.7 Hz, 1H), 7.38 – 7.31 (m, 5H), 7.33 – 7.20 (m, 1H), 7.23 – 7.17 (m, 2H), 7.15 (dd, *J* = 8.6, 1.7 Hz, 1H), 6.60 (dd, *J* = 3.7, 0.8 Hz, 1H), 4.89 (dd, *J* = 9.0, 4.3 Hz, 1H), 3.09 (dd, *J* = 13.8, 4.3 Hz, 1H), 3.01 (dd, *J* = 13.8, 9.0 Hz, 1H), 2.34 (s, 3H), 1.89 (broad s, 1H).

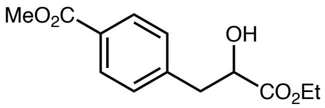

Methyl 4-(3-ethoxy-2-hydroxy-3-oxopropyl)benzoate[18] (24): Prepared according to general procedure B using methyl 4-bromobenzoate (0.1 mmol, 21.5 mg, 1.0 equiv), sodium carbonate (0.2 mmol, 21.2 mg, 2.0 equiv), (Ir[dF(CF_3)ppy]$_2$(5,5'-dCF_3bpy))PF_6 (0.001 mmol, 1.1 mg, 1 mol%), $(TMS)_3$SiNHAdm (0.3 mmol, 119 mg, 3.0 equiv), TBAI (0.25 mmol, 9.2 mg, 25 mol%), and ethyl acrylate (0.3 mmol, 0.033 mL, 3 equiv) in 5% H_2O in MeCN (5 mL). The reaction was purified by preparative TLC (20% EtOAc/Hex) to afford the title compound as a yellow oil (7.2 mg, 29% yield). The physical data and spectral properties match previously reported values.
^{1}H NMR (400 MHz, $CDCl_3$) δ 7.97 (d, *J* = 8.3 Hz, 2H), 7.31 (d, *J* = 8.3 Hz, 2H), 4.46 (dd, *J* = 11.5, 5.6, 1H), 4.22 (q, *J* = 7.1 Hz, 2H), 3.90 (s, 3H), 3.18 (dd, *J* = 13.9, 4.4 Hz, 1H), 3.02 (dd, *J* = 13.9, 6.8 Hz, 1H), 2.82 (d, *J* = 5.7 Hz, 1H), 1.28 (t, *J* = 7.1, 3H).

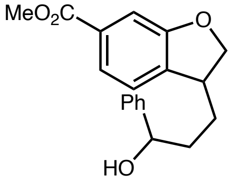

Methyl 3-(3-hydroxy-3-phenylpropyl)-2,3-dihydrobenzofuran-6-carboxylate (25): Prepared according to general procedure B Methyl 3-(allyloxy)-4-bromobenzoate (**S17**) (0.1 mmol, 27.1 mg, 1.0 equiv), sodium carbonate (0.2 mmol, 21.2 mg, 2.0 equiv), (Ir[dF(CF_3)ppy]$_2$(5,5'-dCF_3bpy))PF_6 (0.001 mmol, 1.1 mg, 1 mol%), $(TMS)_3$SiNHAdm (0.3 mmol, 119 mg, 3.0 equiv), TBAI (0.25 mmol, 9.2 mg, 25 mol%), and styrene (0.3 mmol, 0.034 mL, 3 equiv) in 5% H_2O in MeCN (5 mL). The reaction was purified by preparative TLC (20% EtOAc/Hex) and isolated as a 1.0 : 1.0 diastereomeric mixture (* denotes a diastereomeric peak) to afford the title compound as a yellow oil (12.0 mg, 39% yield).
^{1}H NMR (600 MHz, $CDCl_3$) δ 7.57 (dd, *J* = 3.0, 1.5 Hz, 1H*), 7.56 (dd, *J* = 3.0, 1.5 Hz, 1H*), 7.40 – 7.28 (m, 6H), 7.18 (d, *J* = 3.9 Hz, 1H*), 7.17 (d, *J* = 3.9 Hz, 1H*), 4.72–4.67 (m, 1H), 4.66 (t, *J* = 8.9 Hz, 1H*), 4.66 (t, *J* = 8.9 Hz 1H*) 4.25 (dd *J* = 10.9, 8.8 Hz, 1H*), 4.24 (dd, *J* = 10.9, 8.8 Hz, 1H*) 3.88 (s, 3H), 3.52–3.44 (m), 1.99 – 1.88 (m, 1H), 1.86 – 1.67 (m, 4H).
^{13}C NMR (151 MHz, $CDCl_3$) δ 167.1, 160.3, 160.2, 144.4, 136.4, 130.7, 128.8, 128.8, 128.0, 128.0, 126.0, 125.9, 124.2, 124.2, 122.6, 122.6, 110.5, 74.7, 74.6, 52.3, 41.8, 41.8, 36.3, 30.8, 30.7.
HRMS (APCI pos.) m/z: [M+] calcd. for $C_{19}H_{20}O_4$, 312.1356; found 312.1354.

VII. References

(1) Wiles, R. J.; Phelan, J. P.; Molander, G. A. Metal-free defluorinative arylation of trifluoromethyl alkenes via photoredox catalysis. *Chem. Commun.* **2019**, *55*, 7599-7602.
(2) Le, C.; Chen, T. Q.; Liang, T.; Zhang, P.; MacMillan, D. W. C. A radical approach to the copper oxidative addition problem: Trifluoromethylation of bromoarenes. *Science.* **2018**, *360*, 1010-1014.
(3) Sakai, H. A.; Liu, W.; Le, C. C.; MacMillan, D. W. C. Cross-Electrophile Coupling of Unactivated Alkyl Chlorides. *J. Am. Chem. Soc.* **2020**, *142*, 11691-11697.
(4) Maust, M. C.; Hendy, C. M.; Jui, N. T.; Blakey, S. B. Switchable Regioselective 6-endo or 5-exo Radical Cyclization via Photoredox Catalysis. *J. Am. Chem. Soc.* **2022**, *144*, 3776-3781.
(5) Wang, M.-M.; Lu, S.-M.; Li, C. Carbo-Carboxylation of Alkenes via Intramolecular Heck Carbonylation Utilizing CO2 and Hydrosilane. *ACS Catal.* **2022**, *12*, 10801-10807.
(6) Uchiyama, M.; Kameda, M.; Mishima, O.; Yokoyama, N.; Koike, M.; Kondo, Y.; Sakamoto, T. New Formulas for Organozincate Chemistry. *J. Am. Chem. Soc.* **1998**, *120*, 4934-4946.
(7) McDaniel, K. A.; Jui, N. T. Dearomatization through Photoredox Hydroarylation: Discovery of a Radical-Polar Crossover Strategy. *Org. Lett.* **2021**, *23*, 5576-5580.
(8) Perry, G. J. P.; Quibell, J. M.; Panigrahi, A.; Larrosa, I. Transition-Metal-Free Decarboxylative Iodination: New Routes for Decarboxylative Oxidative Cross-Couplings. *J. Am. Chem. Soc.* **2017**, *139*, 11527-11536.
(9) Bloux, H.; Dahiya, A.; Hébert, A.; Fabis, F.; Schoenebeck, F.; Cailly, T. Base-Mediated Radio-Iodination of Arenes by Using Organosilane and Organogermane as Radiolabelling Precursors. *Chem. Euro. J.* **2023**, *29*, e202203366.
(10) Yoo, E. J.; Chang, S. A New Route to Indolines by the Cu-Catalyzed Cyclization Reaction of 2-Ethynylanilines with Sulfonyl Azides. *Org. Lett.* **2008**, *10*, 1163-1166.
(11) Zidan, M.; McCallum, T.; Swann, R.; Barriault, L. Formal Bromine Atom Transfer Radical Addition of Nonactivated Bromoalkanes Using Photoredox Gold Catalysis. *Org. Lett.* **2020**, *22*, 8401-8406.
(12) Dahlén, A.; Petersson, A.; Hilmersson, G. Diastereoselective intramolecular SmI2–H2O–amine mediated couplings. *Org. Biomol. Chem.* **2003**, *1*, 2423-2426.
(13) Andersson, B.; Wikstrom, H.; Hallberg, A. *Acta. Chem. Scand.* **1990**. *44*, 1024-1028.
(14) Fantasia, S.; Windisch, J.; Scalone, M. Ligandless Copper-Catalyzed Coupling of Heteroaryl Bromides with Gaseous Ammonia. *Adv. Synth. Catal.* **2013**, *355*, 627-631.
(15) Hooper, M.; Imam, S. H. 11H-isoindolo[2,1 -a]indol-11-ones: novel rearrangement products from the attempted preparation of 2-(2-diethylaminomethylphenyl)isatogens. *J. Chem. Soc., Perkin Trans. 1.* **1985**, 1583-1587.
(16) Martins, R. S.; Ahmad, A.; Silva, L. F.; Andrade, L. H. Exploiting sequential lipase-catalyzed reactions to achieve enantiomerically pure chiral primary alcohols. *RSC Adv.* **2015**, *5*, 56599-56605.
(17) Wang, H.; Wang, Z.; Zhao, G.; Ramadoss, V.; Tian, L.; Wang, Y. Electrochemical Deoxygenative Barbier-Type Reaction. *Org. Lett.* **2022**, *24*, 3668-3673.
(18) Maust, M. C.; Blakey, S. B. Photoredox-Driven Three-Component Coupling of Aryl Halides, Olefins, and O2. *ACS Catal.* **2024**, *14*, 2582-2587.

VIII. NMR Spectra

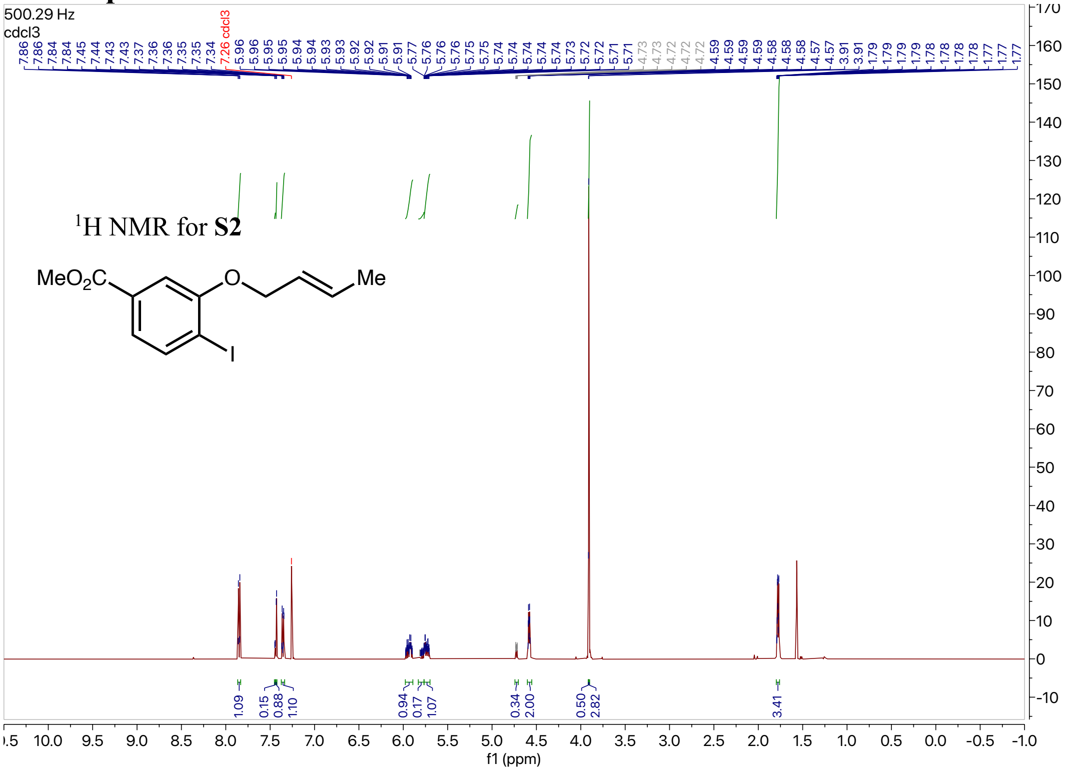

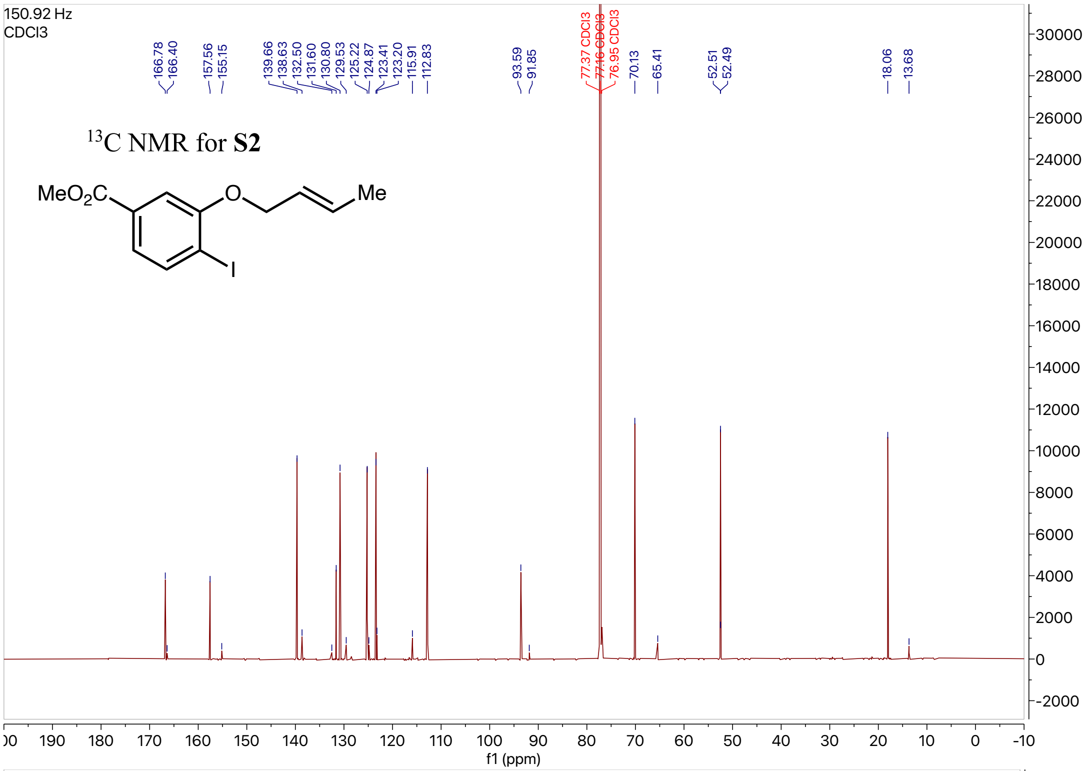
150.92 Hz
CDCl3
166.78
166.40
157.56
155.15
139.66
138.63
132.50
131.60
130.80
129.53
125.22
124.87
123.41
123.20
115.91
112.83
93.59
91.85
77.37 CDCl3
77.16 CDCl3
76.95 CDCl3
70.13
65.41
52.51
52.49
18.06
13.68
^{13}C NMR for **S2**
MeO2C
O
Me
I
f1 (ppm)

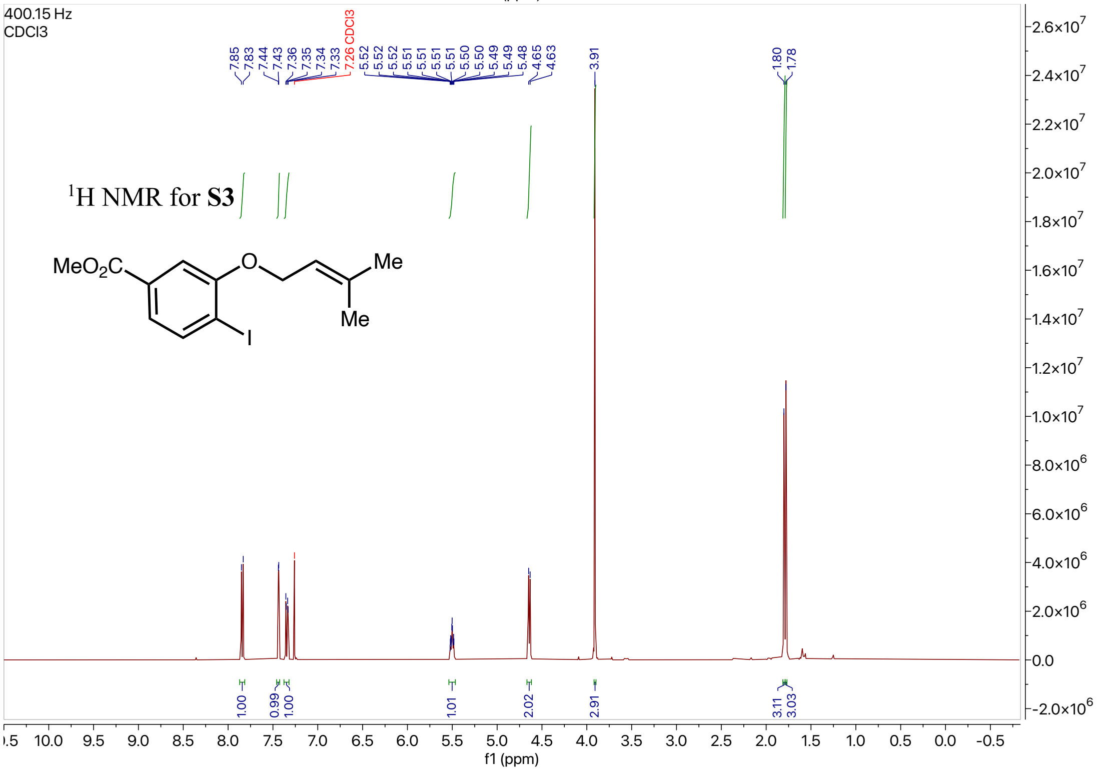
400.15 Hz
CDCl3
7.85
7.83
7.44
7.43
7.36
7.35
7.34
7.33
7.26 CDCl3
5.52
5.52
5.51
5.51
5.51
5.50
5.50
5.49
5.49
5.48
4.65
4.63
3.91
1.80
1.78
^{1}H NMR for **S3**
MeO2C
O
Me
Me
I
1.00
0.99
1.00
1.01
2.02
2.91
3.11
3.03
f1 (ppm)

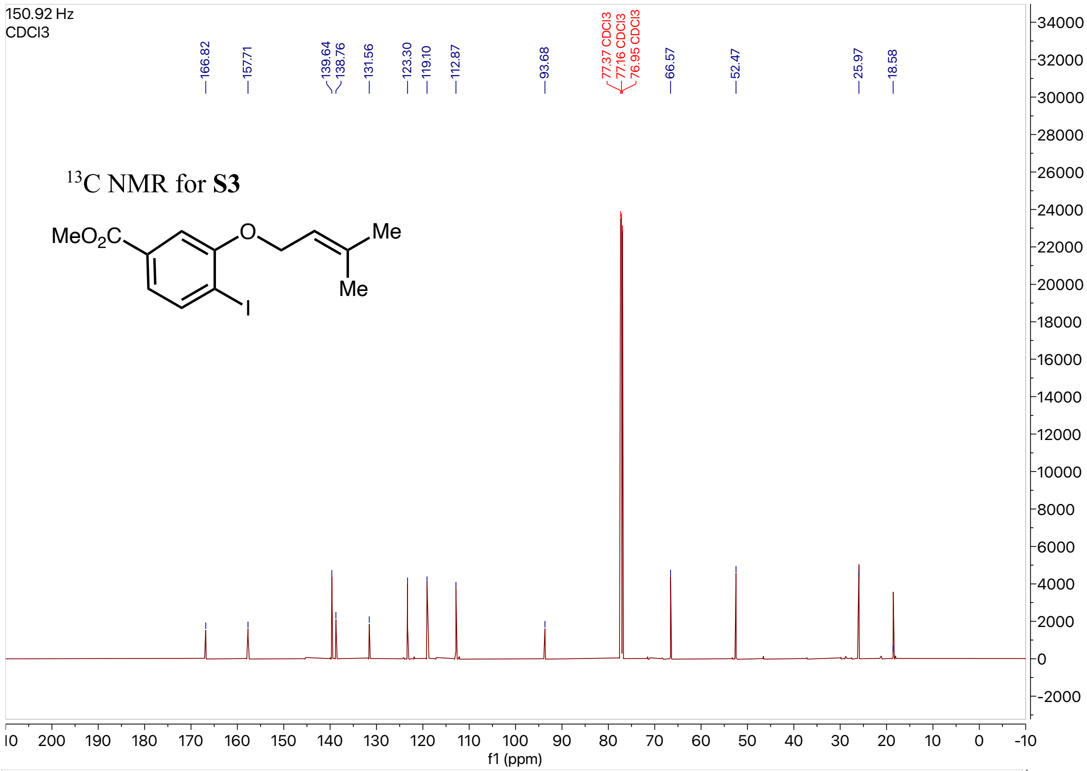
150.92 Hz
CDCl3
166.82
157.71
139.64
138.76
131.56
123.30
119.10
112.87
93.68
77.37 CDCl3
77.16 CDCl3
76.95 CDCl3
66.57
52.47
25.97
18.58
13C NMR for S3
MeO2C
O
Me
Me
I
f1 (ppm)

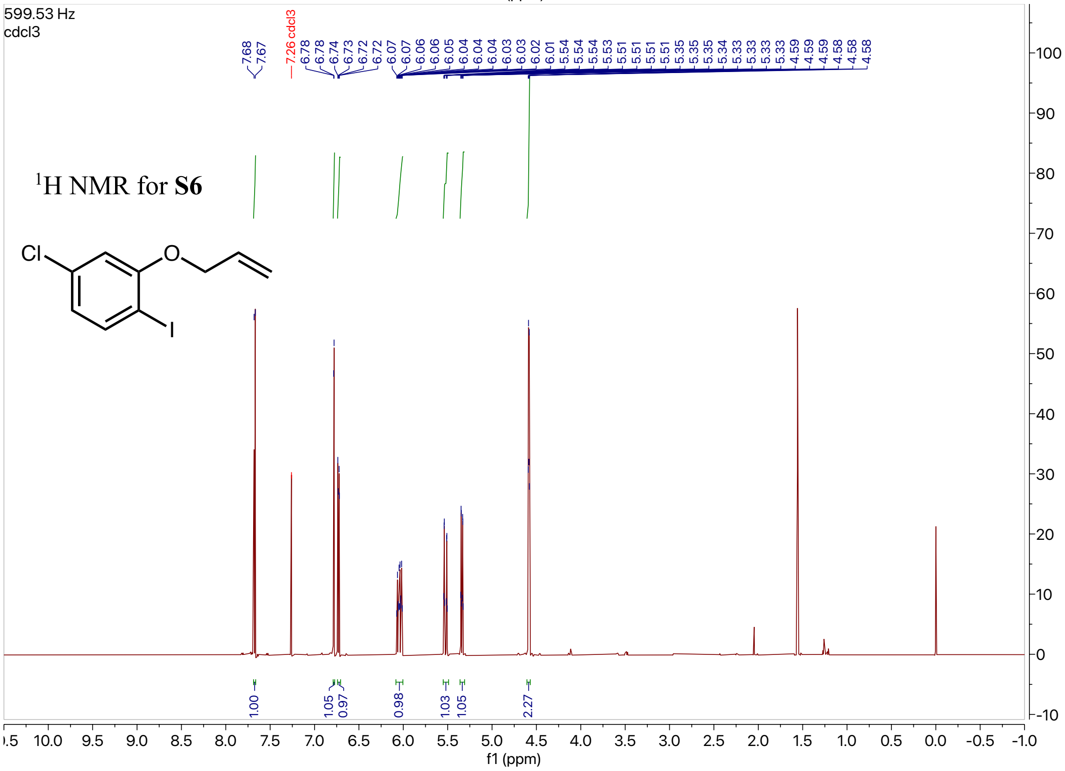
599.53 Hz
cdcl3
7.68
7.67
7.26 cdcl3
6.78
6.78
6.74
6.73
6.72
6.72
6.07
6.07
6.06
6.06
6.05
6.04
6.04
6.04
6.03
6.03
6.02
6.01
5.54
5.54
5.54
5.53
5.51
5.51
5.51
5.51
5.35
5.35
5.35
5.34
5.33
5.33
5.33
5.33
4.59
4.59
4.59
4.58
4.58
4.58
1H NMR for S6
Cl
O
I
1.00
1.05
0.97
0.98
1.03
1.05
2.27
f1 (ppm)

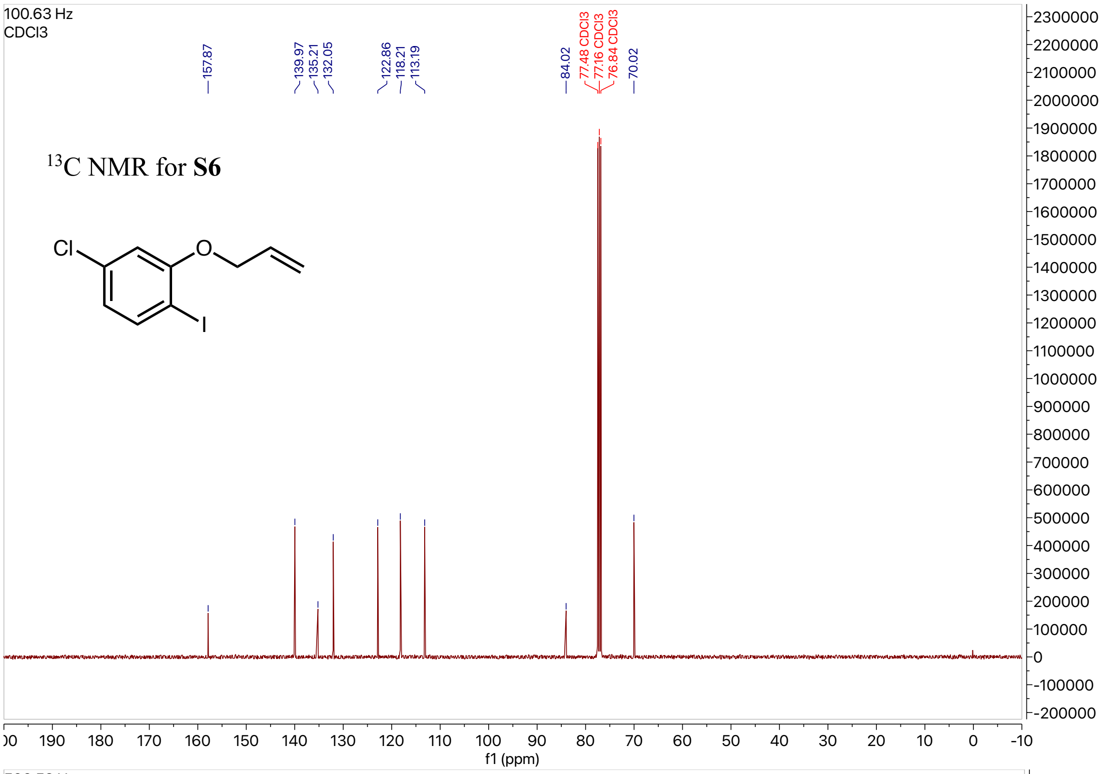
100.63 Hz
CDCl3
157.87
139.97
135.21
132.05
122.86
118.21
113.19
84.02
77.48 CDCl3
77.16 CDCl3
76.84 CDCl3
70.02
^{13}C NMR for **S6**
Cl
O
I
f1 (ppm)

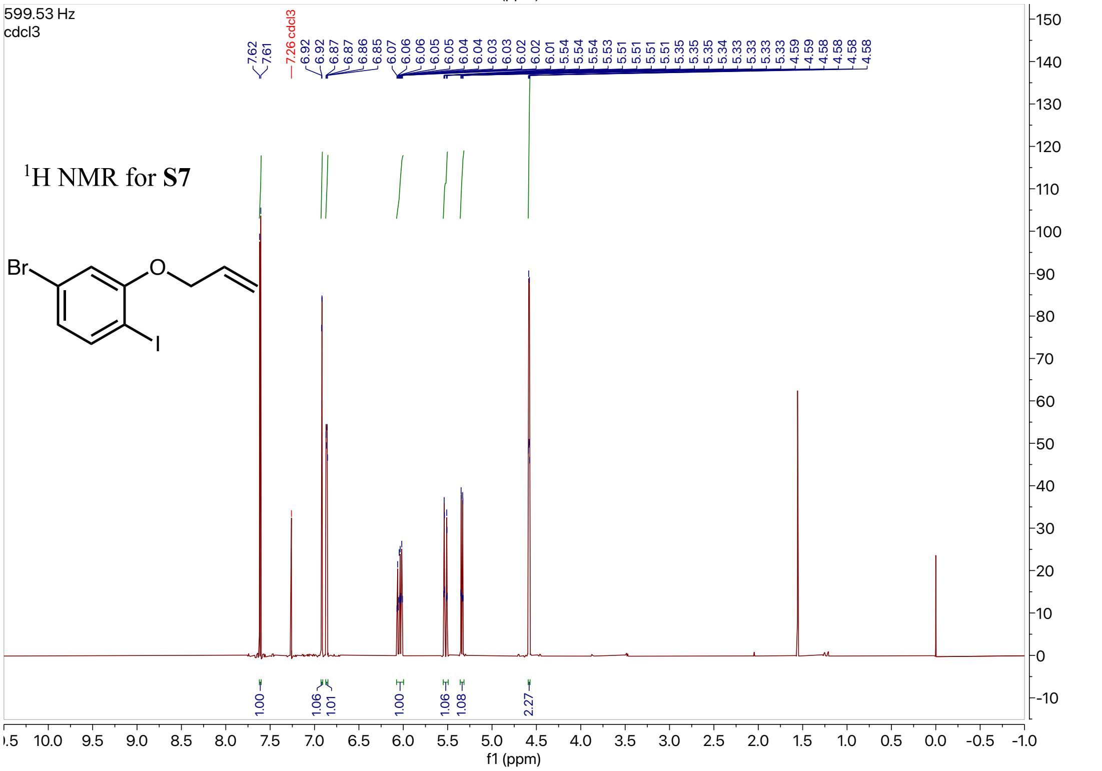
599.53 Hz
cdcl3
7.62
7.61
7.26 cdcl3
6.92
6.92
6.87
6.87
6.86
6.85
6.07
6.06
6.06
6.05
6.05
6.04
6.04
6.03
6.03
6.02
6.02
6.01
5.54
5.54
5.54
5.53
5.51
5.51
5.51
5.51
5.35
5.35
5.35
5.34
5.33
5.33
5.33
5.33
4.59
4.59
4.58
4.58
4.58
4.58
^{1}H NMR for **S7**
Br
O
I
1.00
1.06
1.01
1.00
1.06
1.08
2.27
f1 (ppm)

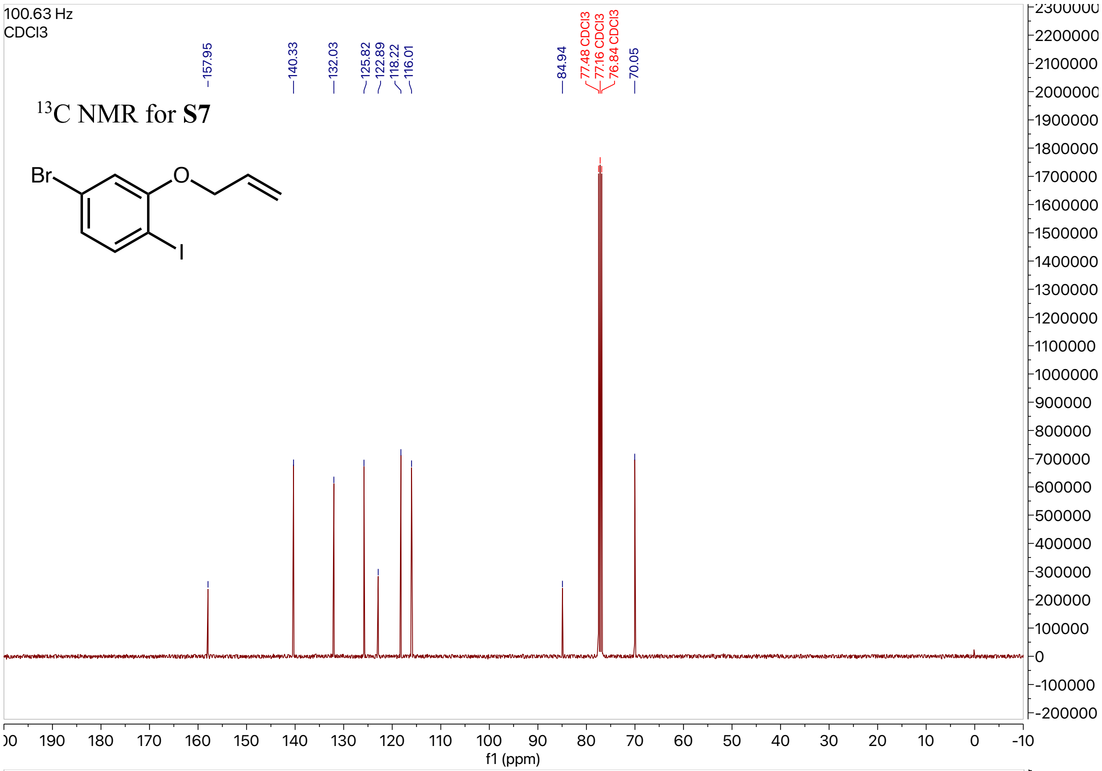
100.63 Hz
CDCl3
^{13}C NMR for **S7**
157.95
140.33
132.03
125.82
122.89
118.22
116.01
84.94
77.48 CDCl3
77.16 CDCl3
76.84 CDCl3
70.05
Br
O
I
f1 (ppm)

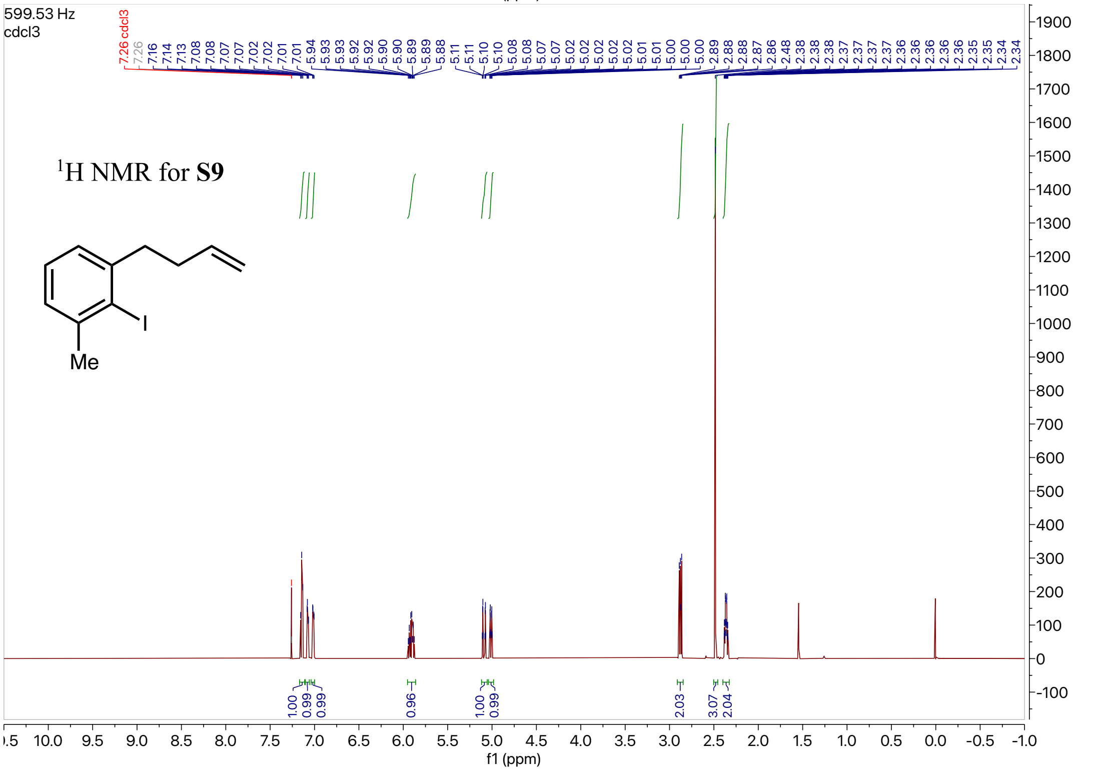
599.53 Hz
cdcl3
^{1}H NMR for **S9**
I
Me
f1 (ppm)
1.00
0.99
0.99
0.96
1.00
0.99
2.03
3.07
2.04

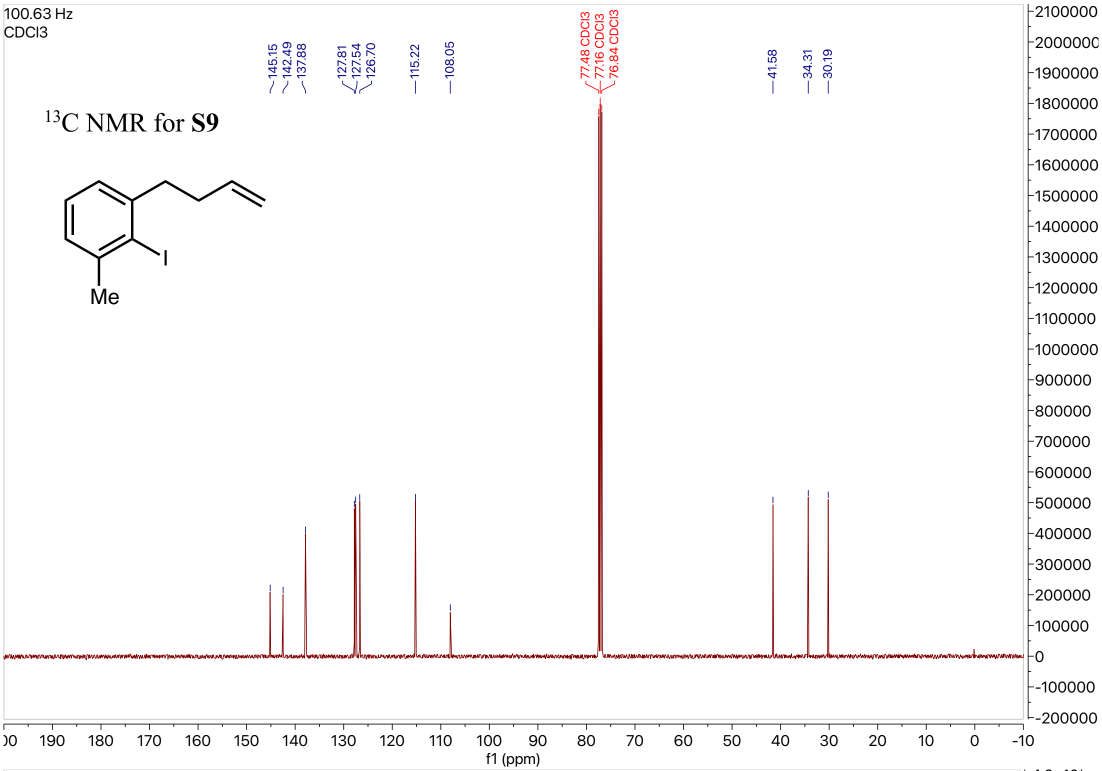
100.63 Hz
CDCl3
13C NMR for S9
Me
I
145.15
142.49
137.88
127.81
127.54
126.70
115.22
108.05
77.48 CDCl3
77.16 CDCl3
76.84 CDCl3
41.58
34.31
30.19
f1 (ppm)

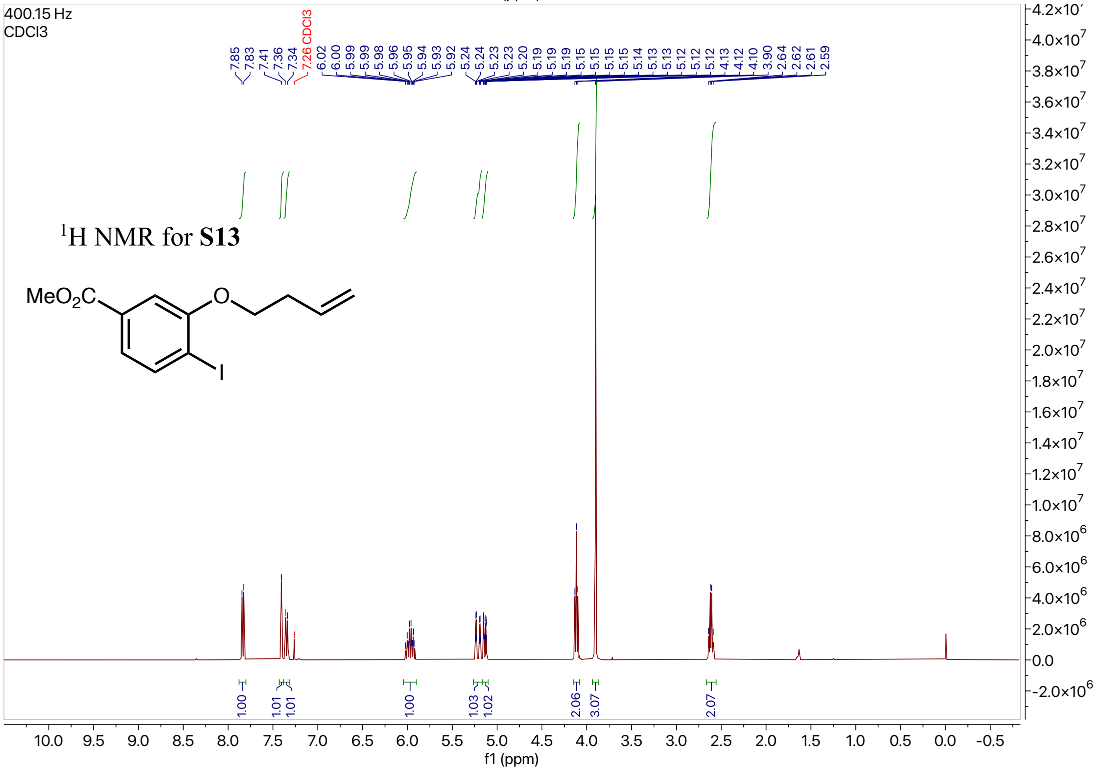
400.15 Hz
CDCl3
1H NMR for S13
MeO2C
O
I
7.85
7.83
7.41
7.36
7.34
7.26 CDCl3
6.02
6.00
5.99
5.99
5.98
5.96
5.95
5.94
5.93
5.92
5.24
5.24
5.23
5.23
5.20
5.19
5.19
5.19
5.15
5.15
5.15
5.14
5.13
5.13
5.12
5.12
5.12
4.13
4.12
4.10
3.90
2.64
2.62
2.61
2.59
1.00
1.01
1.01
1.00
1.03
1.02
2.06
3.07
2.07
f1 (ppm)

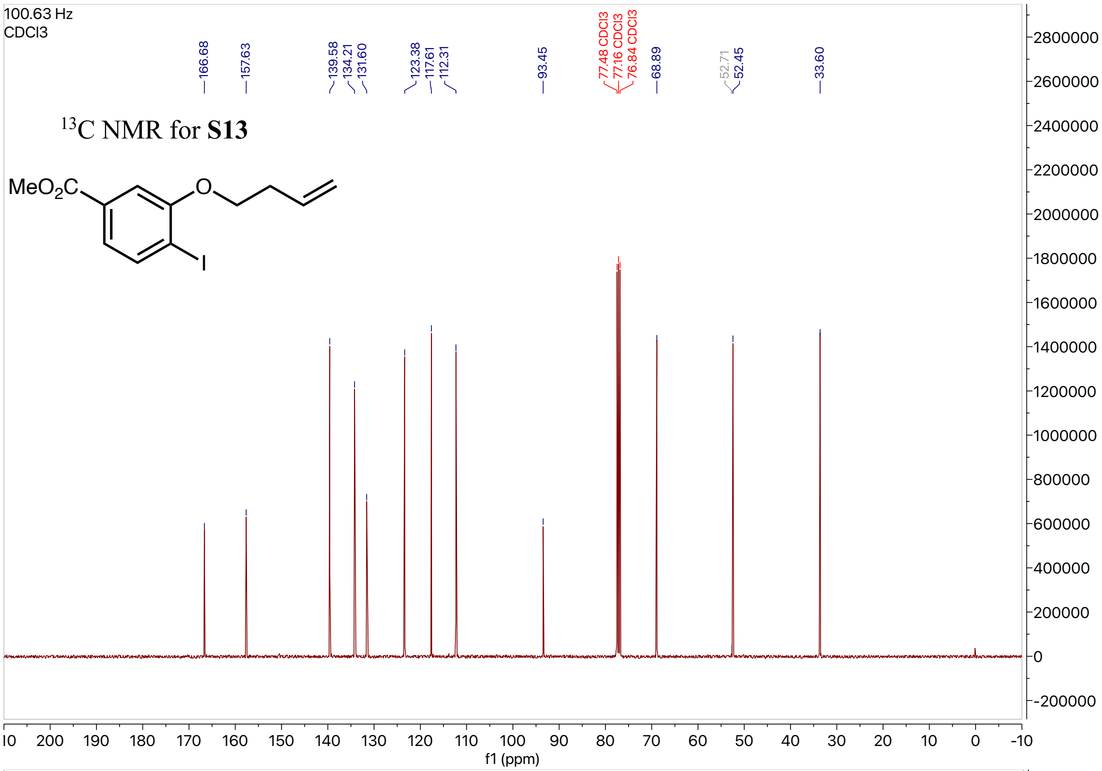
100.63 Hz
CDCl3
166.68
157.63
139.58
134.21
131.60
123.38
117.61
112.31
93.45
77.48 CDCl3
77.16 CDCl3
76.84 CDCl3
68.89
52.71
52.45
33.60
13C NMR for S13
MeO2C
O
I
f1 (ppm)

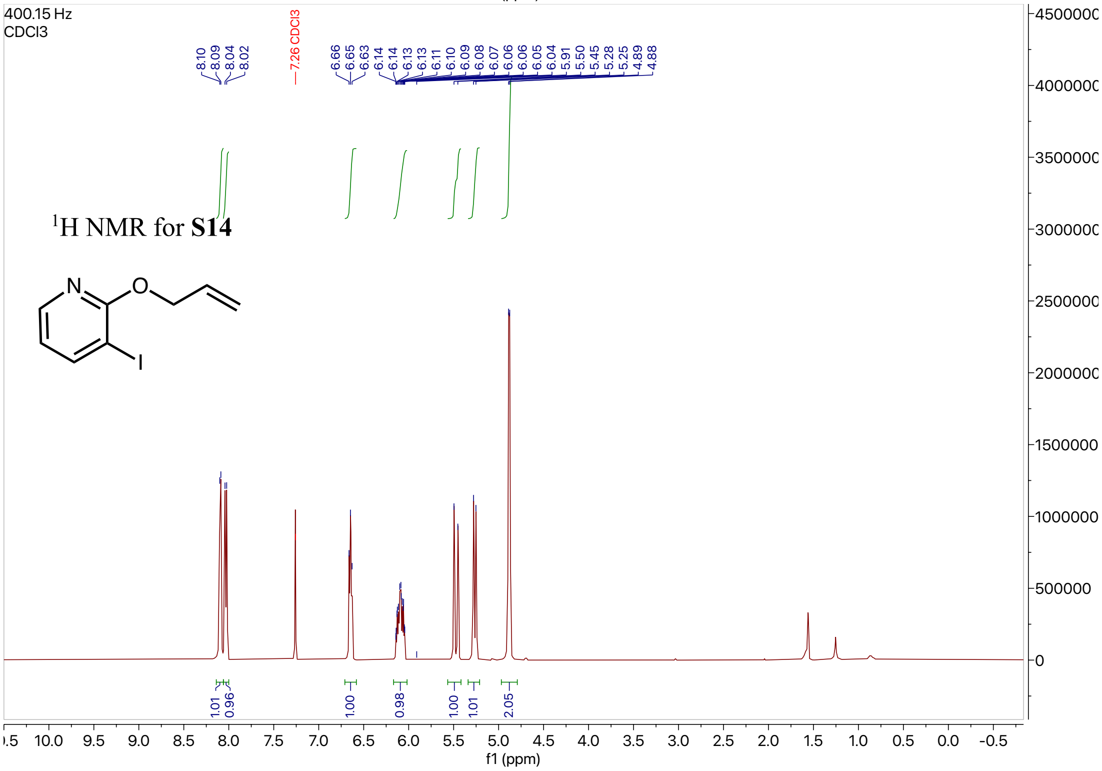
400.15 Hz
CDCl3
8.10
8.09
8.04
8.02
7.26 CDCl3
6.66
6.65
6.63
6.14
6.13
6.13
6.11
6.10
6.09
6.08
6.07
6.06
6.06
6.05
6.04
5.91
5.50
5.45
5.28
5.25
4.89
4.88
1H NMR for S14
N
O
I
1.01
0.96
1.00
0.98
1.00
1.01
2.05
f1 (ppm)

13C NMR for **S14**

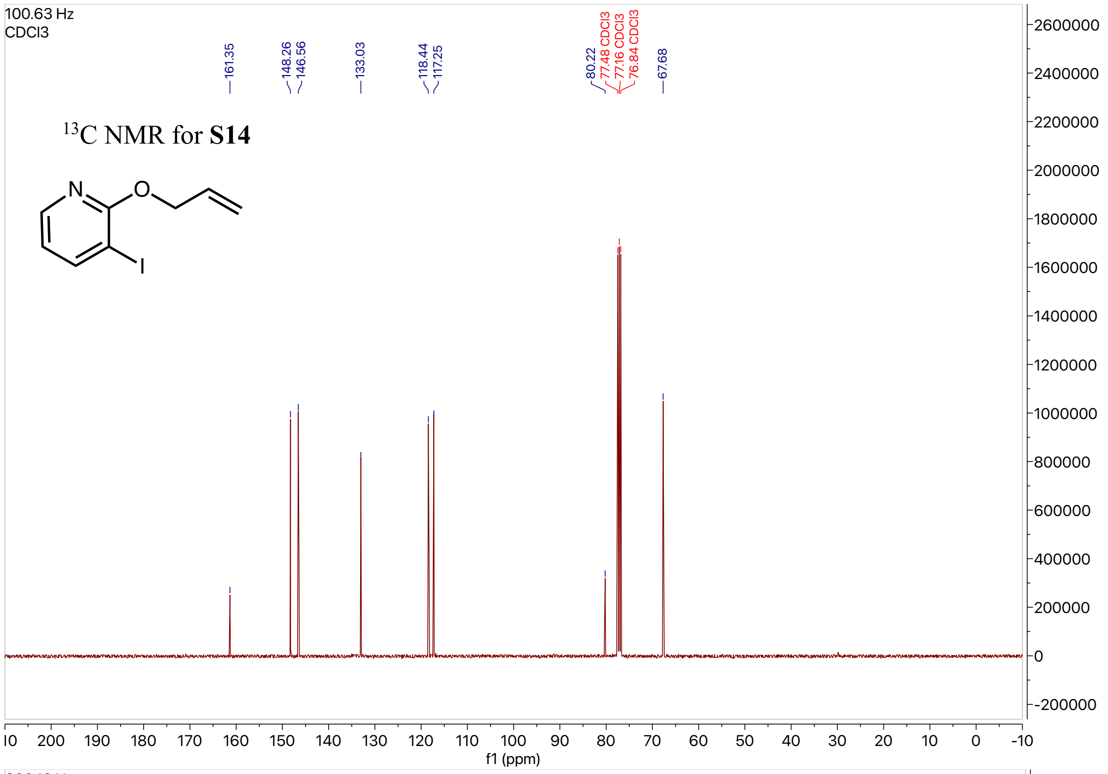

1H NMR for **S16**

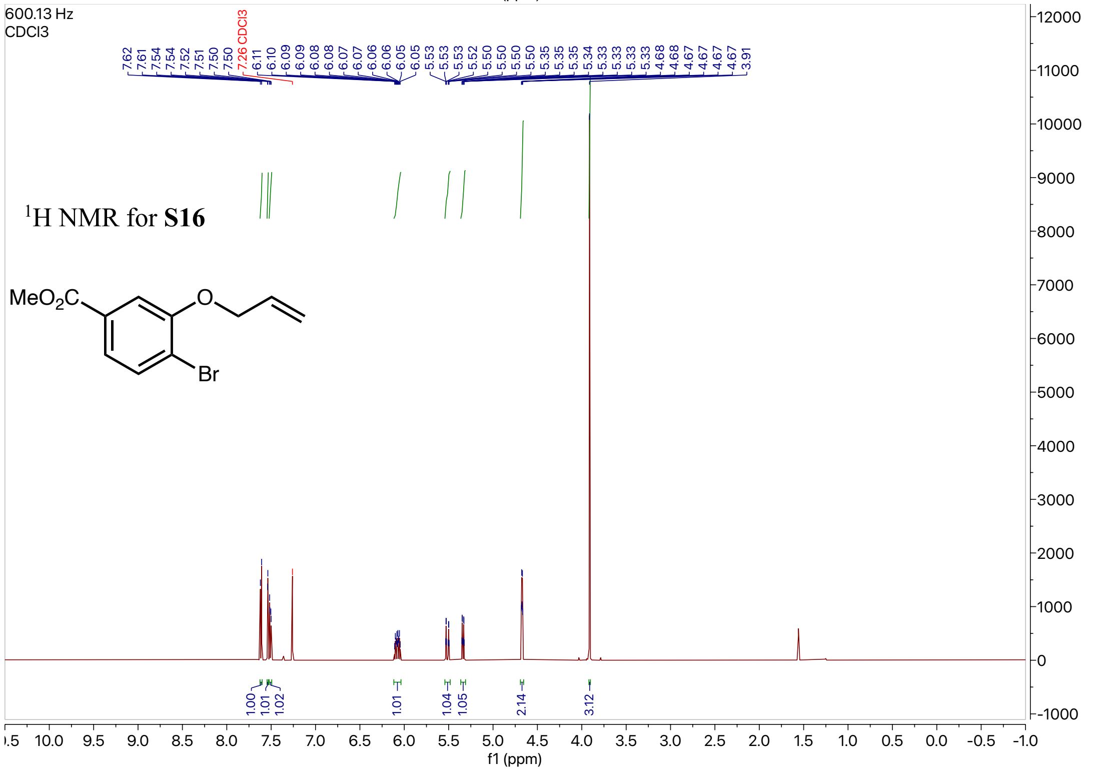

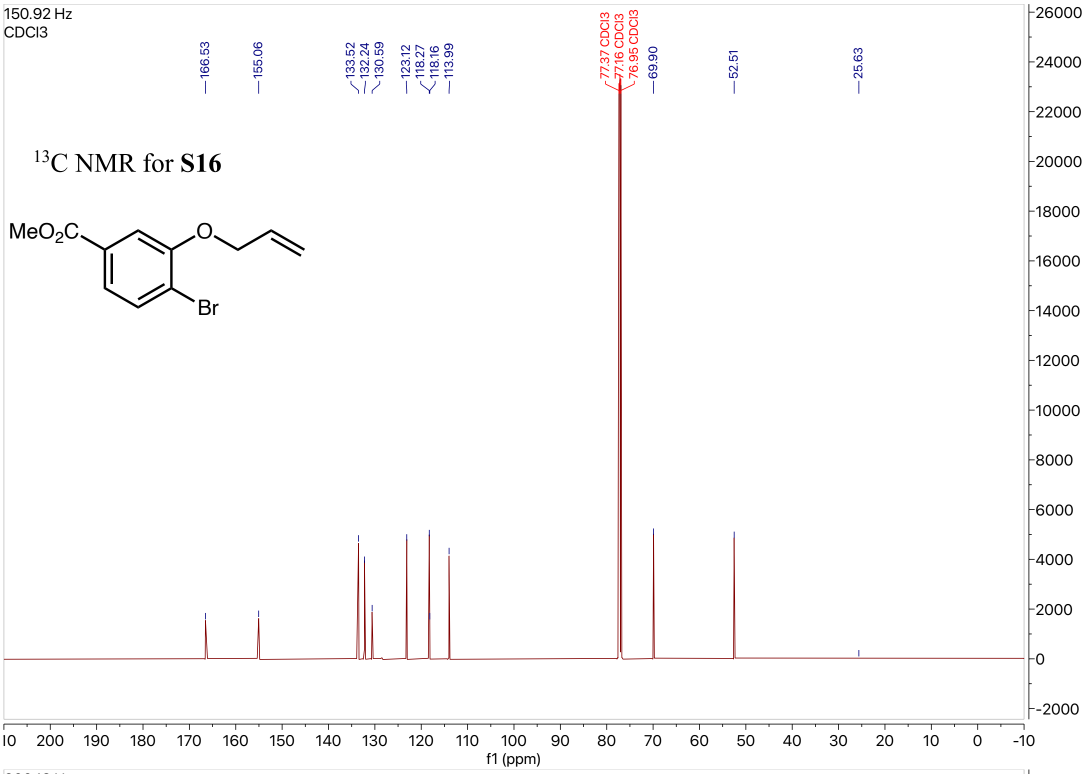
150.92 Hz
CDCl3
166.53
155.06
133.52
132.24
130.59
123.12
118.27
118.16
113.99
77.37 CDCl3
77.16 CDCl3
76.95 CDCl3
69.90
52.51
25.63
13C NMR for S16
MeO2C
O
Br
f1 (ppm)

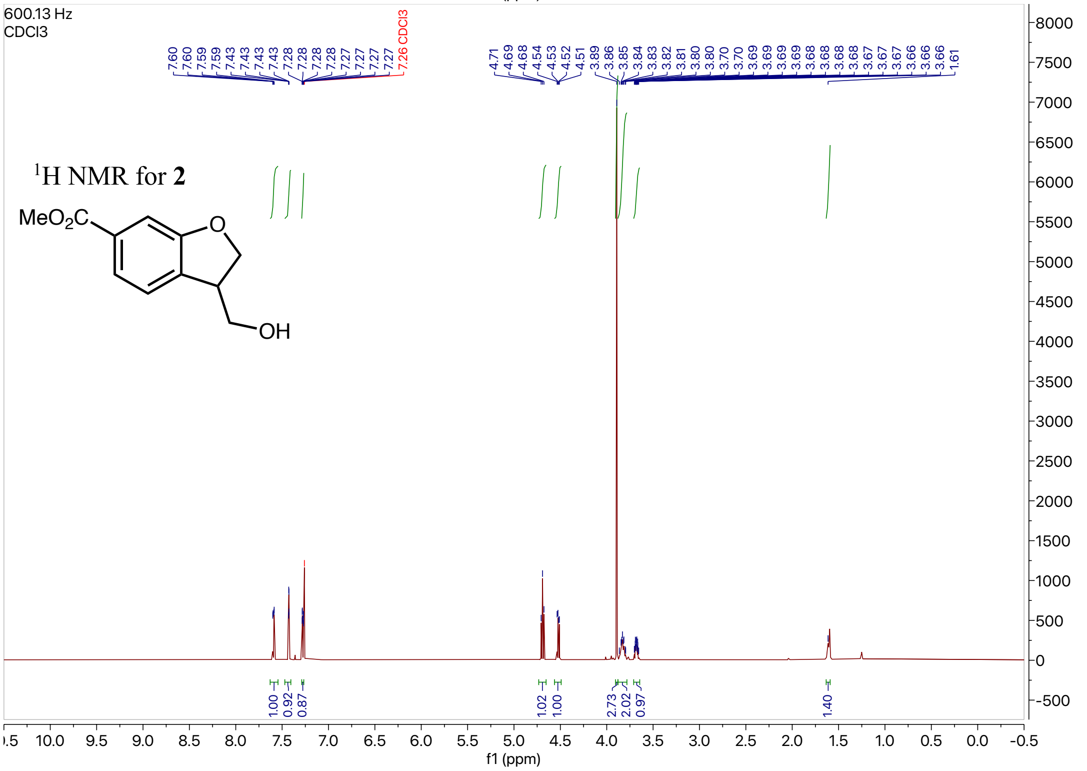
600.13 Hz
CDCl3
7.26 CDCl3
1H NMR for 2
MeO2C
O
OH
1.00
0.92
0.87
1.02
1.00
2.73
2.02
0.97
1.40
f1 (ppm)

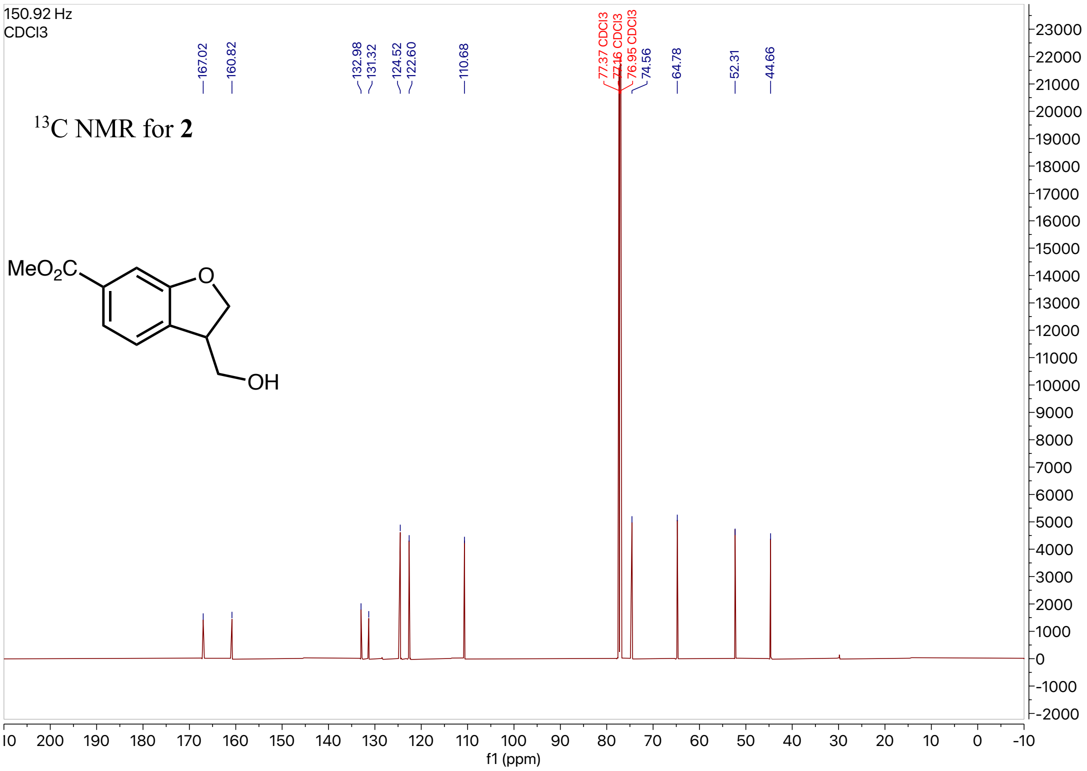
150.92 Hz
CDCl3
167.02
160.82
132.98
131.32
124.52
122.60
110.68
77.37 CDCl3
77.16 CDCl3
76.95 CDCl3
74.56
64.78
52.31
44.66
^{13}C NMR for 2
MeO2C
O
OH
f1 (ppm)

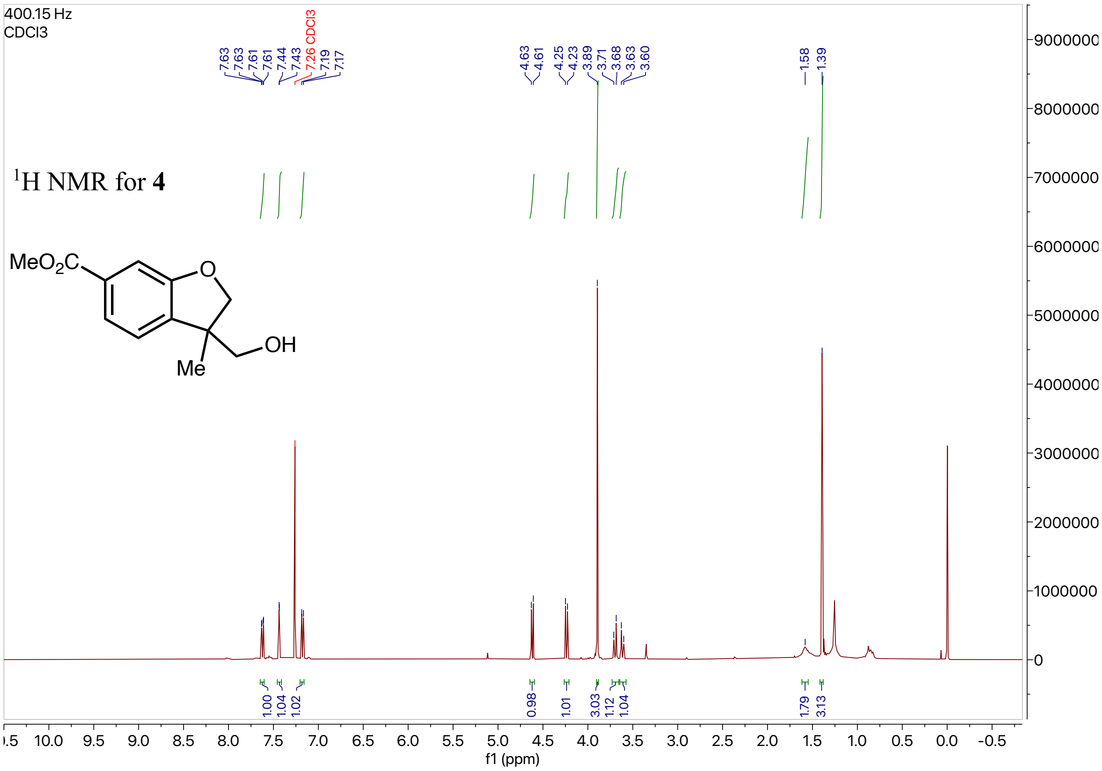
400.15 Hz
CDCl3
7.63
7.63
7.61
7.61
7.44
7.43
7.26 CDCl3
7.19
7.17
4.63
4.61
4.25
4.23
3.89
3.71
3.68
3.63
3.60
1.58
1.39
^{1}H NMR for 4
MeO2C
O
OH
Me
1.00
1.04
1.02
0.98
1.01
3.03
1.12
1.04
1.79
3.13
f1 (ppm)

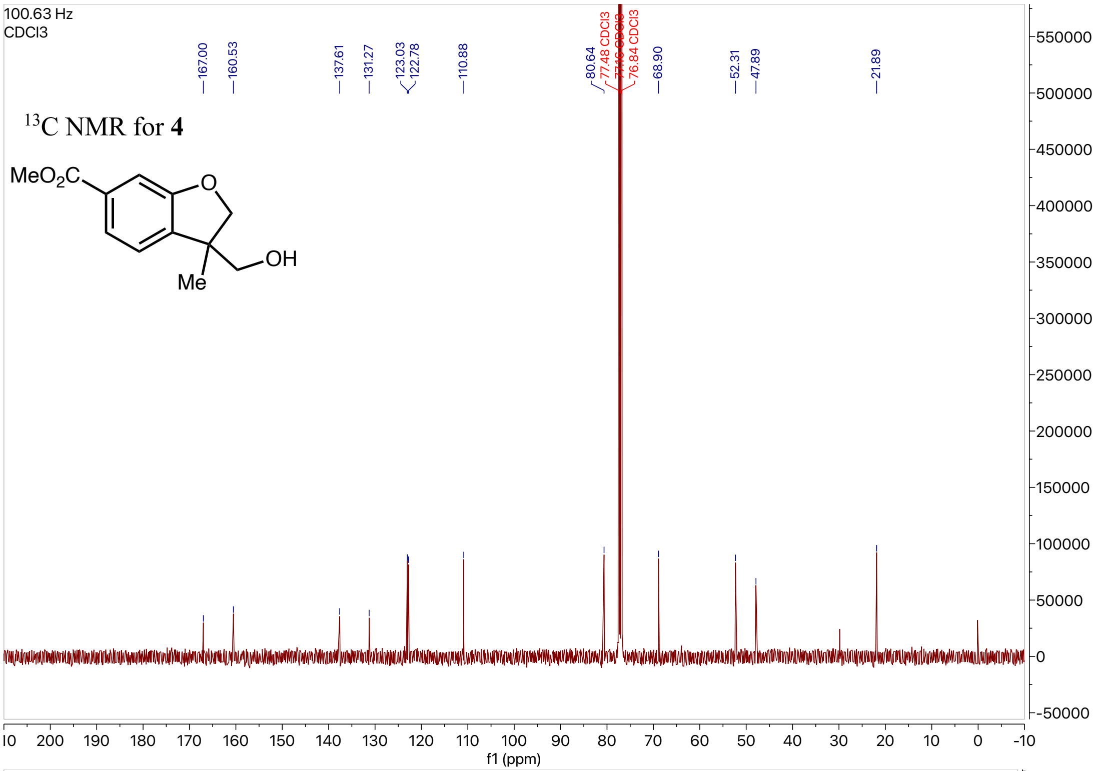
100.63 Hz
CDCl3
167.00
160.53
137.61
131.27
123.03
122.78
110.88
80.64
77.48 CDCl3
76.84 CDCl3
68.90
52.31
47.89
21.89
^{13}C NMR for **4**
MeO2C
O
OH
Me
f1 (ppm)

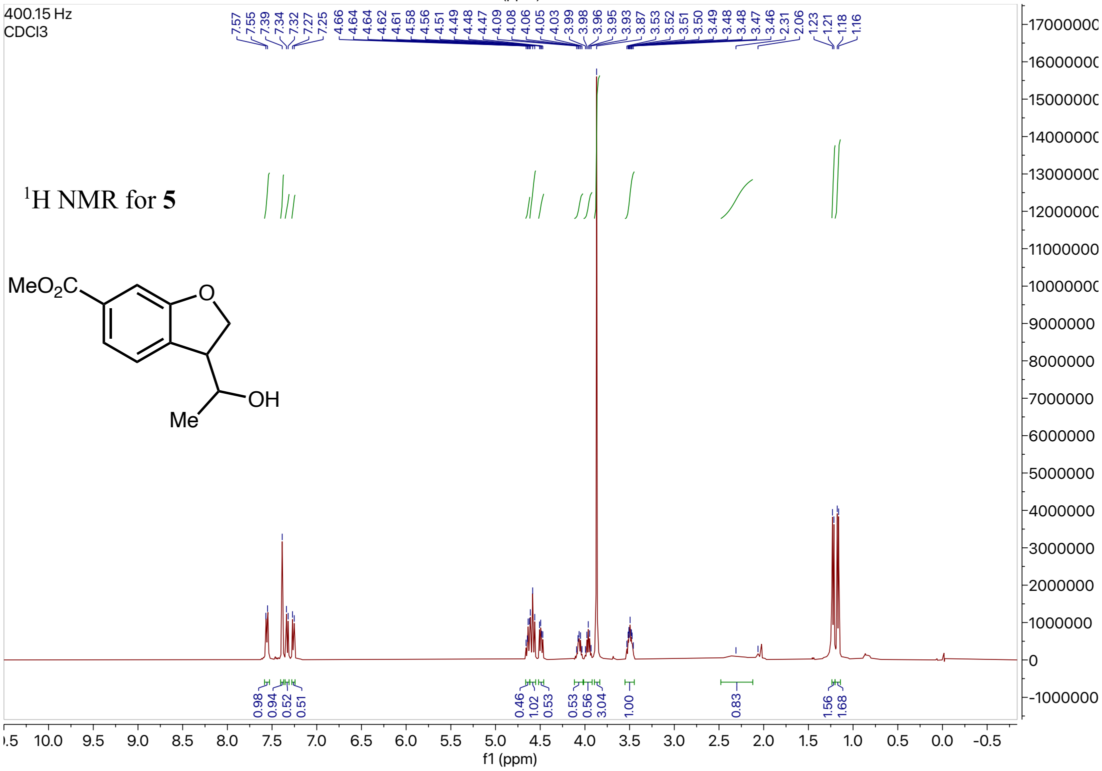
400.15 Hz
CDCl3
7.57
7.55
7.39
7.34
7.32
7.27
7.25
4.66
4.64
4.62
4.61
4.58
4.56
4.51
4.49
4.48
4.47
4.09
4.08
4.06
4.05
4.03
3.99
3.98
3.96
3.95
3.93
3.87
3.53
3.52
3.51
3.50
3.49
3.48
3.47
3.46
2.31
2.06
1.23
1.21
1.18
1.16
^{1}H NMR for **5**
MeO2C
O
OH
Me
0.98
0.94
0.52
0.51
0.46
1.02
0.53
0.53
0.56
3.04
1.00
0.83
1.56
1.68
f1 (ppm)

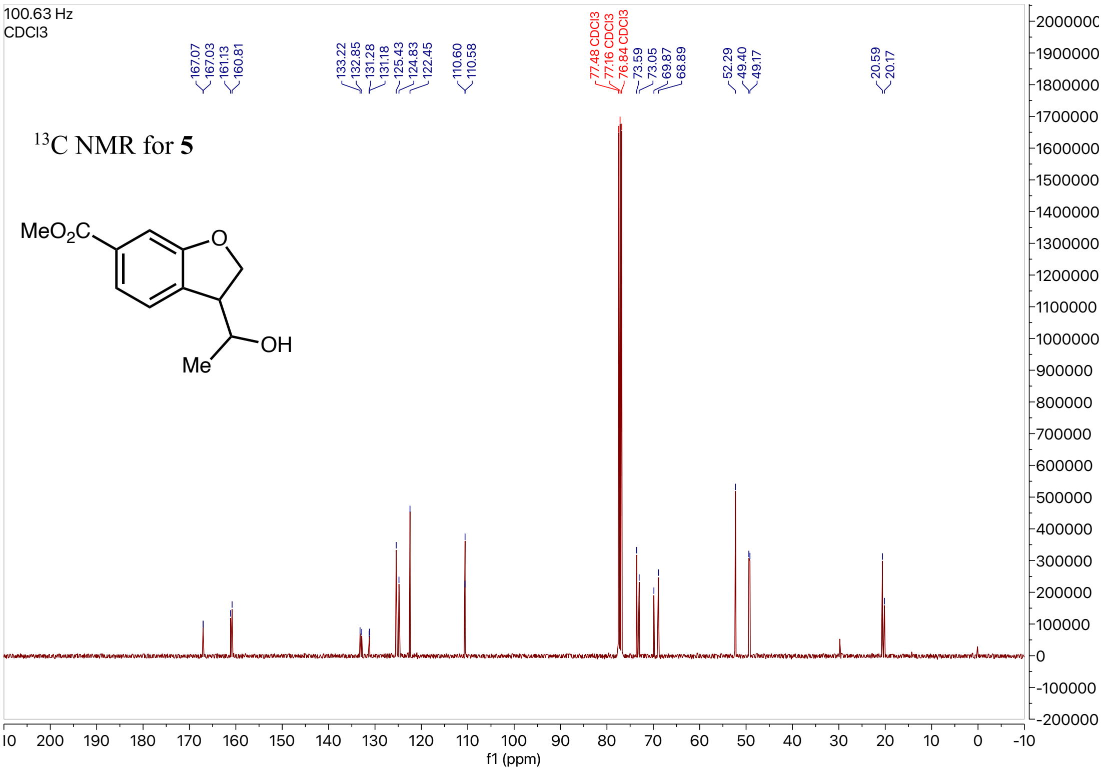
100.63 Hz
CDCl3
167.07
167.03
161.13
160.81
133.22
132.85
131.28
131.18
125.43
124.83
122.45
110.60
110.58
77.48 CDCl3
77.16 CDCl3
76.84 CDCl3
73.59
73.05
69.87
68.89
52.29
49.40
49.17
20.59
20.17
^{13}C NMR for 5
MeO_2C
O
OH
Me
f1 (ppm)

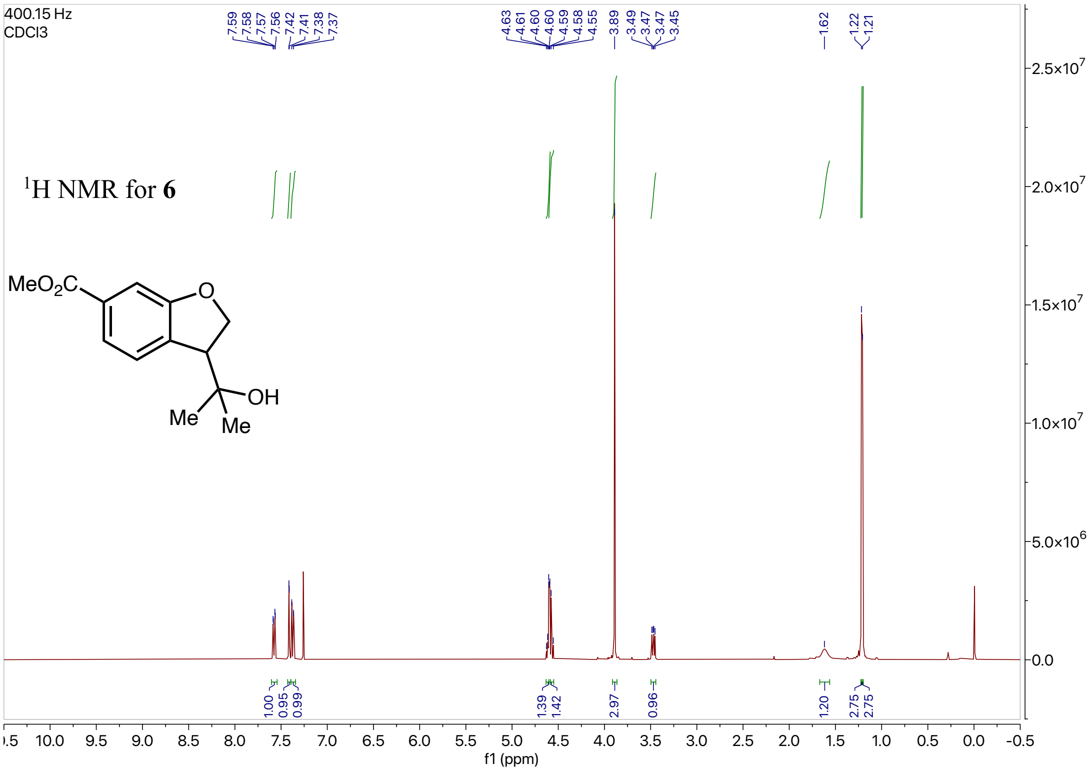
400.15 Hz
CDCl3
7.59
7.58
7.57
7.56
7.42
7.41
7.38
7.37
4.63
4.61
4.60
4.60
4.59
4.58
4.55
3.89
3.49
3.47
3.47
3.45
1.62
1.22
1.21
^{1}H NMR for 6
MeO_2C
O
OH
Me
Me
1.00
0.95
0.99
1.39
1.42
2.97
0.96
1.20
2.75
2.75
f1 (ppm)

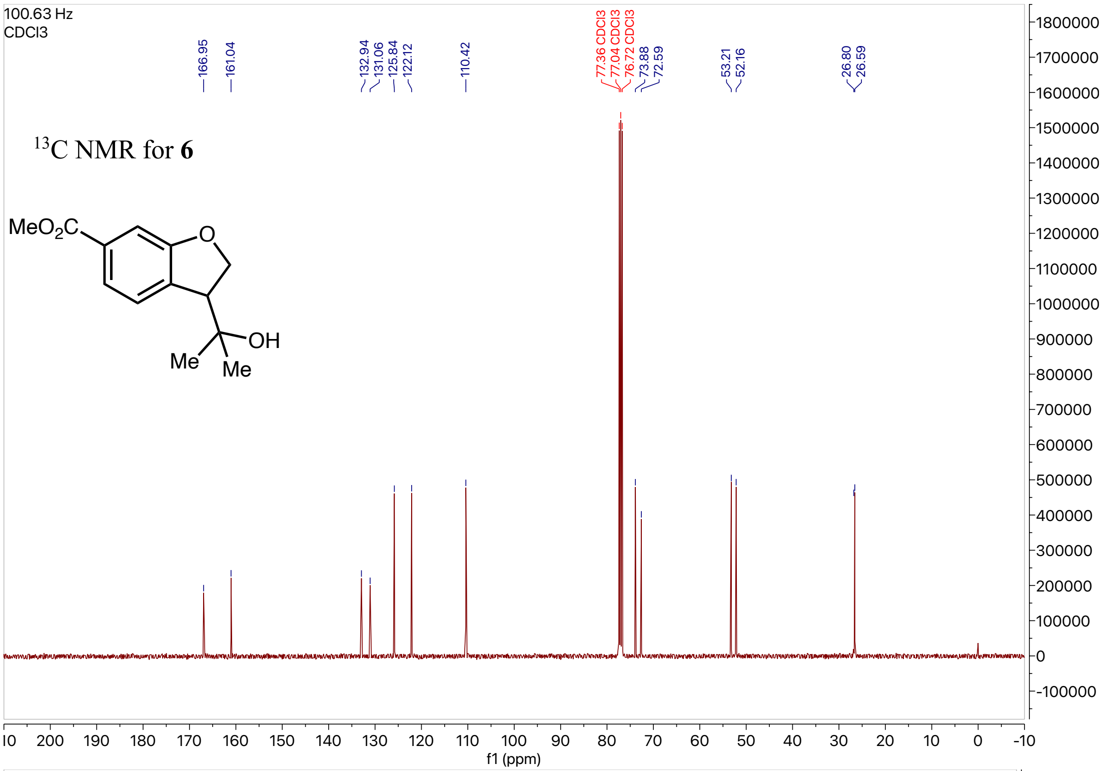
100.63 Hz
CDCl3
166.95
161.04
132.94
131.06
125.84
122.12
110.42
77.36 CDCl3
77.04 CDCl3
76.72 CDCl3
73.88
72.59
53.21
52.16
26.80
26.59
^{13}C NMR for **6**
MeO$_2$C
O
OH
Me
Me
f1 (ppm)

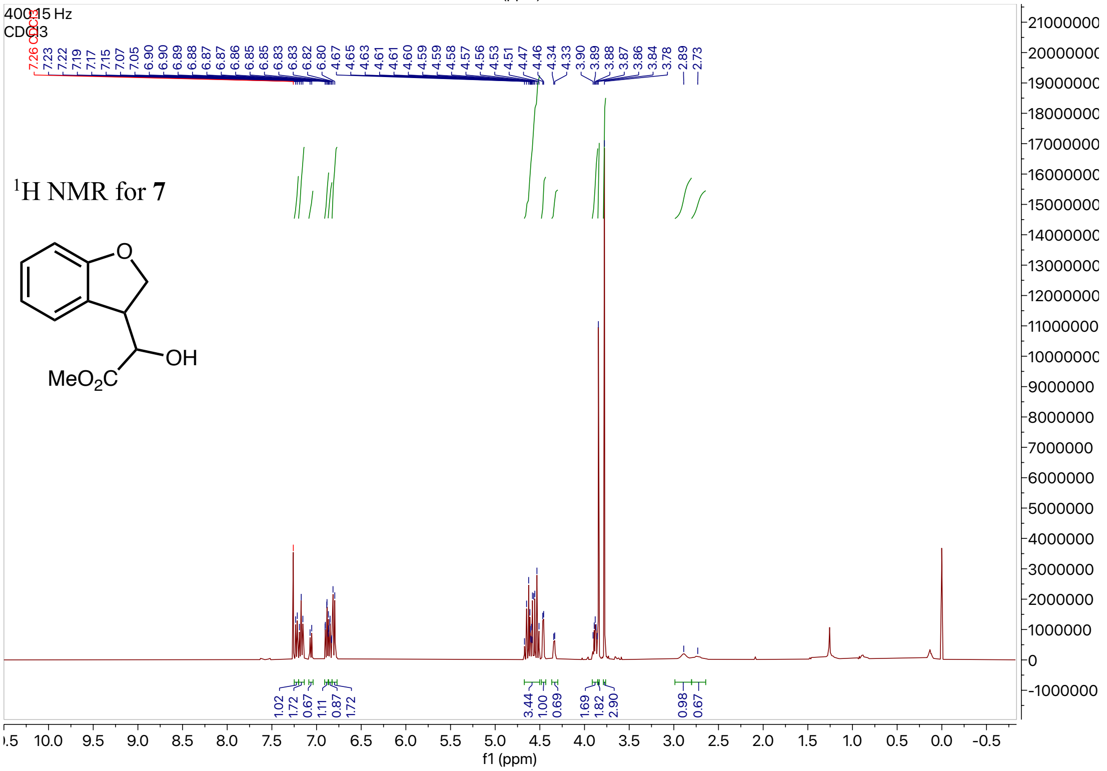
400.15 Hz
CDCl3
7.26 CDCl3
7.23
7.22
7.19
7.17
7.15
7.07
7.05
6.90
6.90
6.89
6.88
6.87
6.87
6.86
6.85
6.85
6.83
6.83
6.82
6.80
4.67
4.65
4.63
4.61
4.61
4.60
4.59
4.59
4.58
4.57
4.56
4.53
4.51
4.47
4.46
4.34
4.33
3.90
3.89
3.88
3.87
3.86
3.84
3.78
2.89
2.73
^{1}H NMR for **7**
O
OH
MeO$_2$C
1.02
1.72
0.67
1.11
0.87
1.72
3.44
1.00
0.69
1.69
1.82
2.90
0.98
0.67
f1 (ppm)

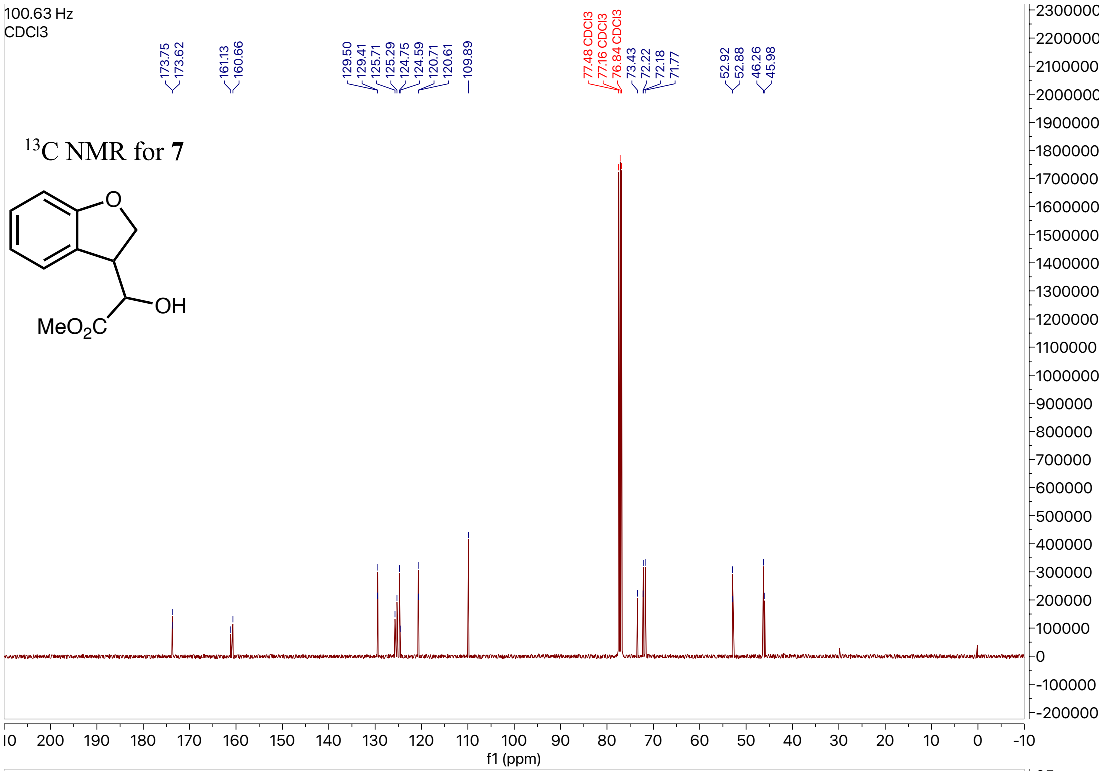
100.63 Hz
CDCl3
13C NMR for 7
MeO2C
OH
O
173.75
173.62
161.13
160.66
129.50
129.41
125.71
125.29
124.75
124.59
120.71
120.61
109.89
77.48 CDCl3
77.16 CDCl3
76.84 CDCl3
73.43
72.22
72.18
71.77
52.92
52.88
46.26
45.98
f1 (ppm)

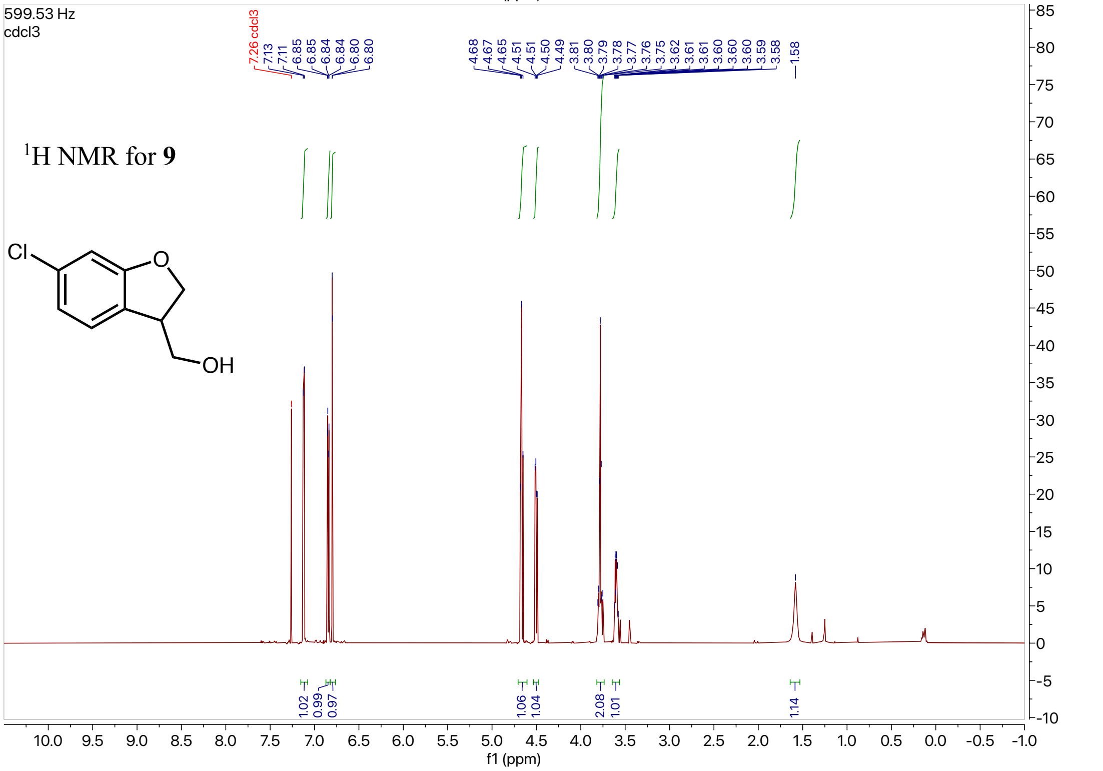
599.53 Hz
cdcl3
1H NMR for 9
Cl
O
OH
7.26 cdcl3
7.13
7.11
6.85
6.85
6.84
6.84
6.80
6.80
4.68
4.67
4.65
4.51
4.51
4.50
4.49
3.81
3.80
3.79
3.78
3.77
3.76
3.75
3.62
3.61
3.60
3.60
3.60
3.59
3.58
1.58
1.02
0.99
0.97
1.06
1.04
2.08
1.01
1.14
f1 (ppm)

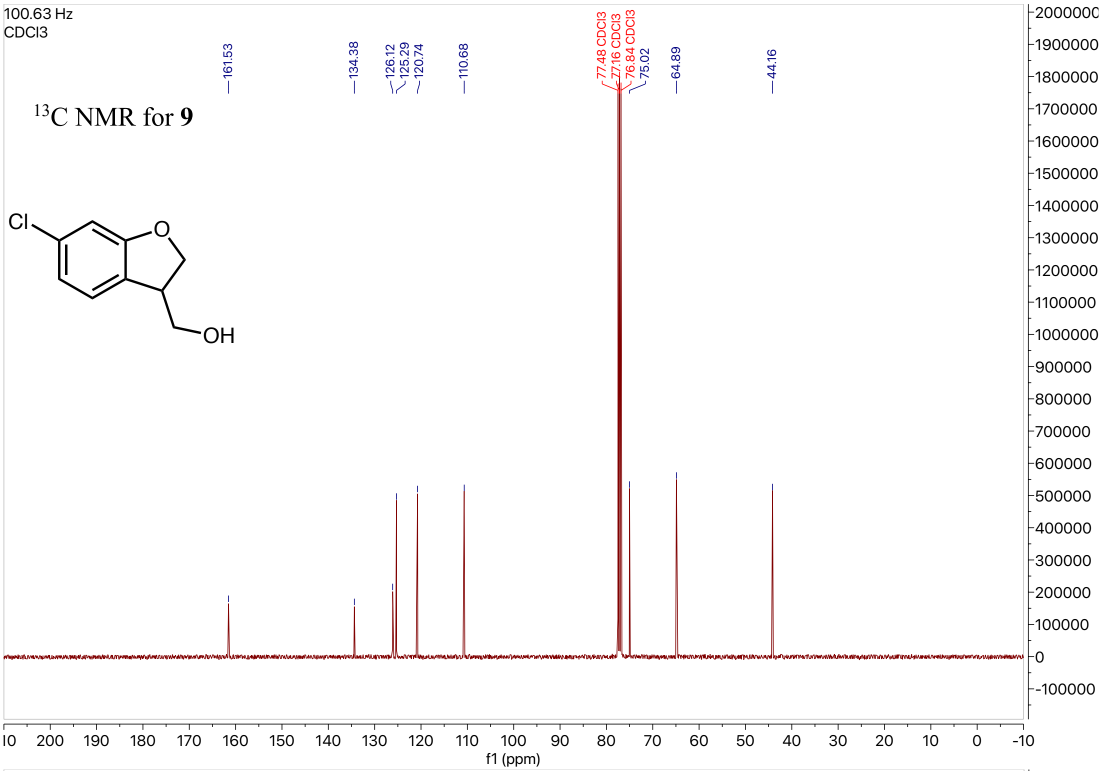
100.63 Hz
CDCl3
^{13}C NMR for **9**
161.53
134.38
126.12
125.29
120.74
110.68
77.48 CDCl3
77.16 CDCl3
76.84 CDCl3
75.02
64.89
44.16
Cl
O
OH
f1 (ppm)

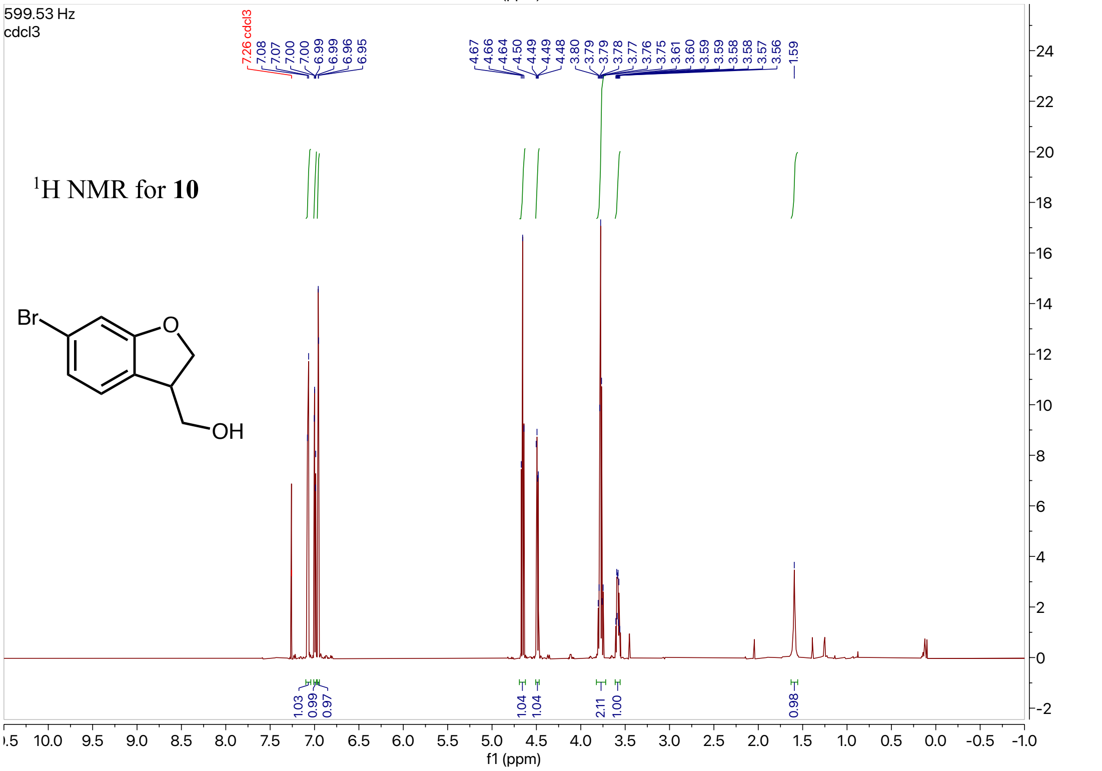
599.53 Hz
cdcl3
^{1}H NMR for **10**
7.26 cdcl3
7.08
7.07
7.00
7.00
6.99
6.99
6.96
6.95
4.67
4.66
4.64
4.50
4.49
4.49
4.48
3.80
3.79
3.79
3.78
3.77
3.76
3.75
3.61
3.60
3.59
3.59
3.58
3.58
3.57
3.56
1.59
1.03
0.99
0.97
1.04
1.04
2.11
1.00
0.98
Br
O
OH
f1 (ppm)

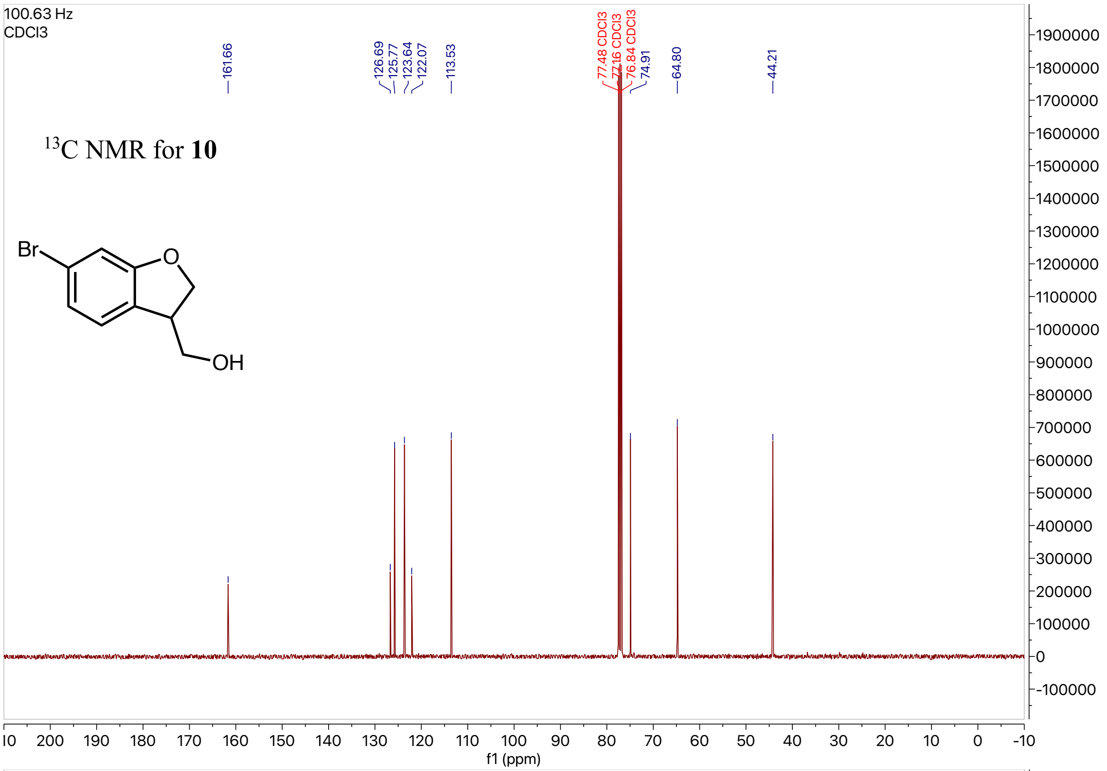
100.63 Hz
CDCl3
161.66
126.69
125.77
123.64
122.07
113.53
77.48 CDCl3
77.16 CDCl3
76.84 CDCl3
74.91
64.80
44.21
13C NMR for 10
Br
O
OH
f1 (ppm)

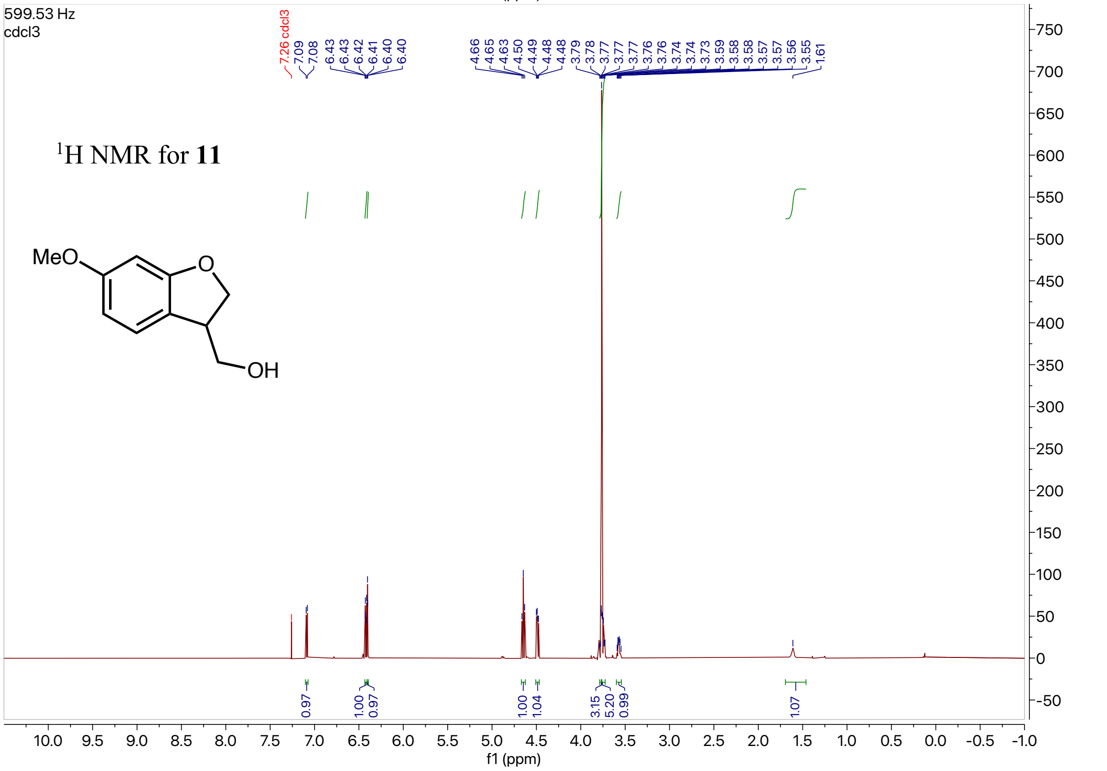
599.53 Hz
cdcl3
7.26 cdcl3
7.09
7.08
6.43
6.43
6.42
6.41
6.40
6.40
4.66
4.65
4.63
4.50
4.49
4.48
4.48
3.79
3.78
3.77
3.77
3.77
3.76
3.76
3.74
3.74
3.73
3.73
3.59
3.58
3.58
3.57
3.57
3.56
3.55
1.61
1H NMR for 11
MeO
O
OH
0.97
1.00
0.97
1.00
1.04
3.15
5.20
0.99
1.07
f1 (ppm)

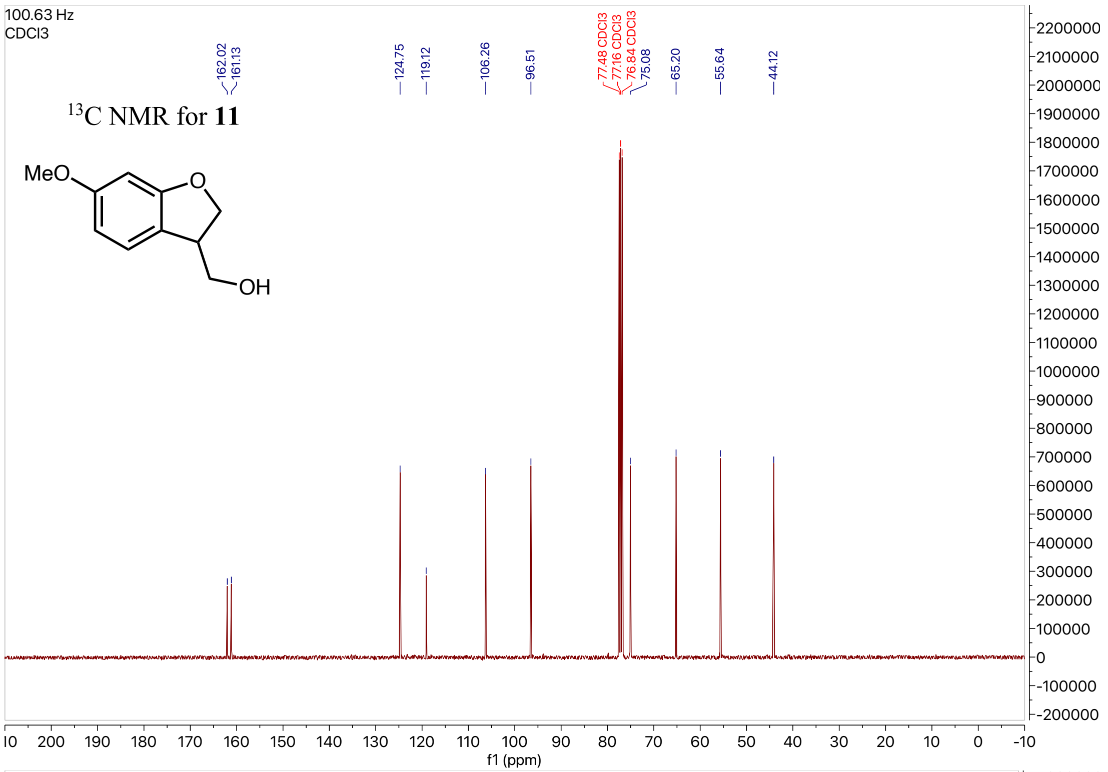
100.63 Hz
CDCl3
13C NMR for 11
162.02
161.13
124.75
119.12
106.26
96.51
77.48 CDCl3
77.16 CDCl3
76.84 CDCl3
75.08
65.20
55.64
44.12
MeO
O
OH
f1 (ppm)

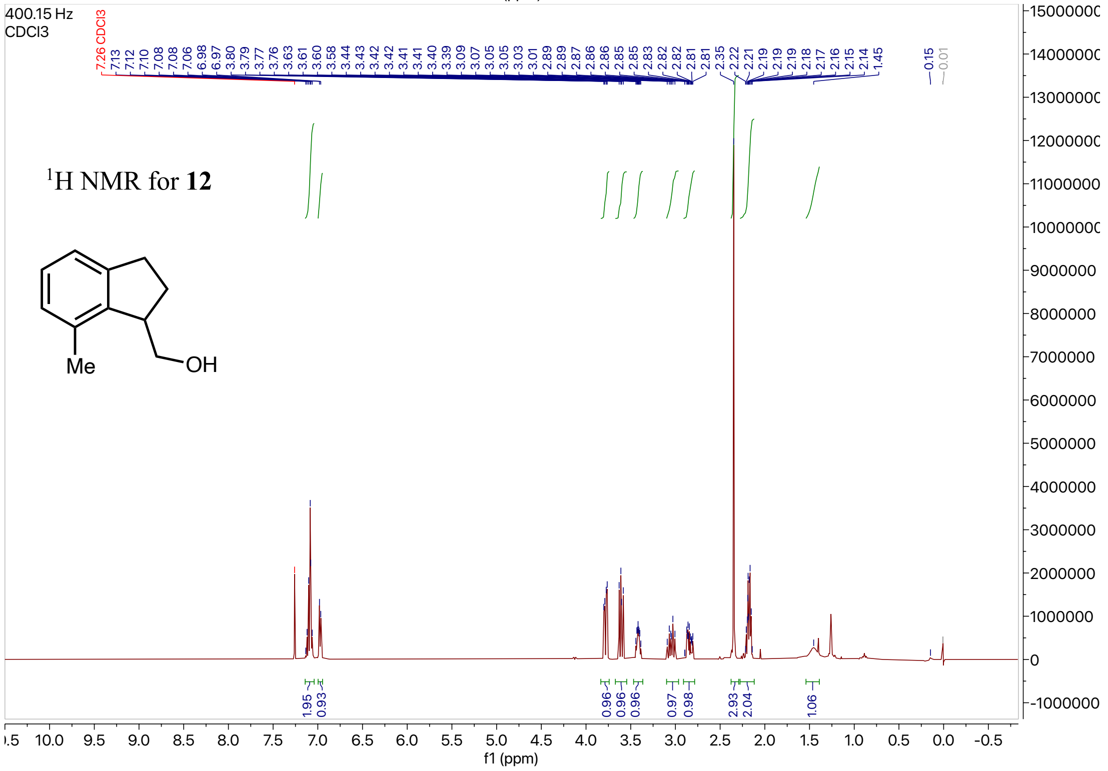
400.15 Hz
CDCl3
1H NMR for 12
7.26 CDCl3
7.13
7.12
7.10
7.08
7.08
7.06
6.98
6.97
3.80
3.79
3.77
3.76
3.63
3.61
3.60
3.58
3.44
3.43
3.42
3.42
3.41
3.41
3.40
3.39
3.09
3.07
3.05
3.05
3.03
3.01
2.89
2.89
2.87
2.86
2.86
2.85
2.85
2.83
2.82
2.82
2.81
2.81
2.35
2.22
2.21
2.19
2.19
2.19
2.18
2.17
2.16
2.15
2.14
1.45
0.15
-0.01
1.95
0.93
0.96
0.96
0.96
0.97
0.98
2.93
2.04
1.06
Me
OH
f1 (ppm)

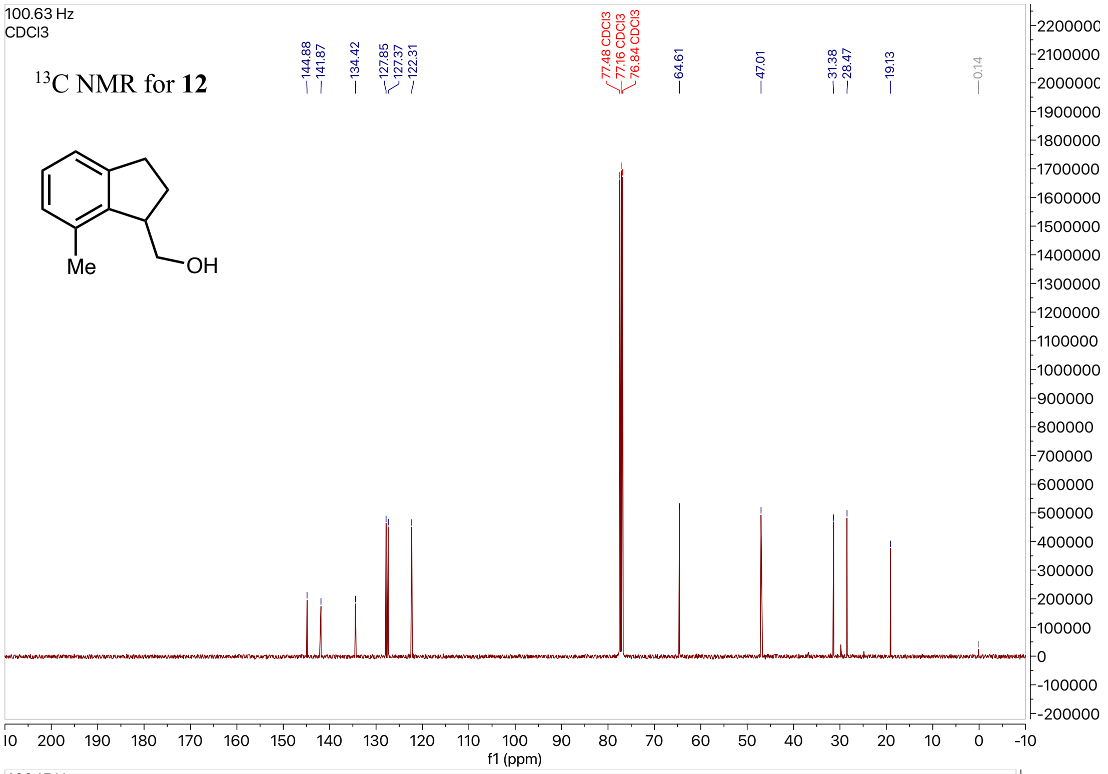
100.63 Hz
CDCl3
13C NMR for 12
Me
OH
f1 (ppm)

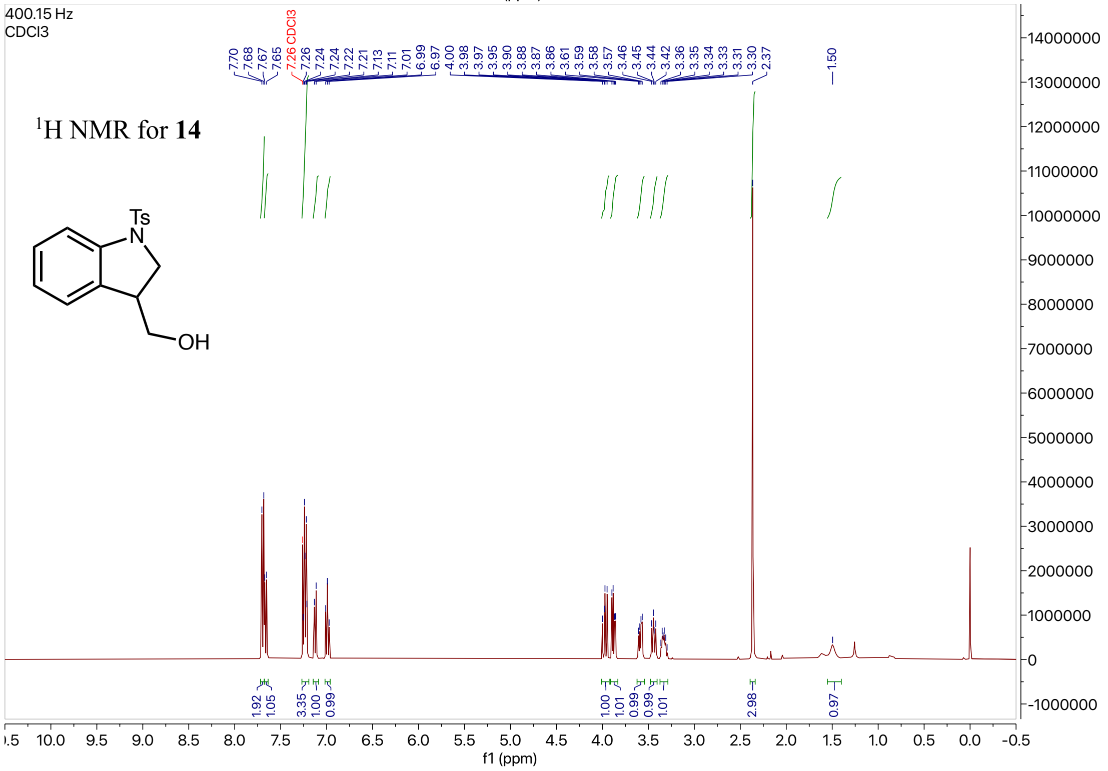
400.15 Hz
CDCl3
1H NMR for 14
Ts
N
OH
f1 (ppm)

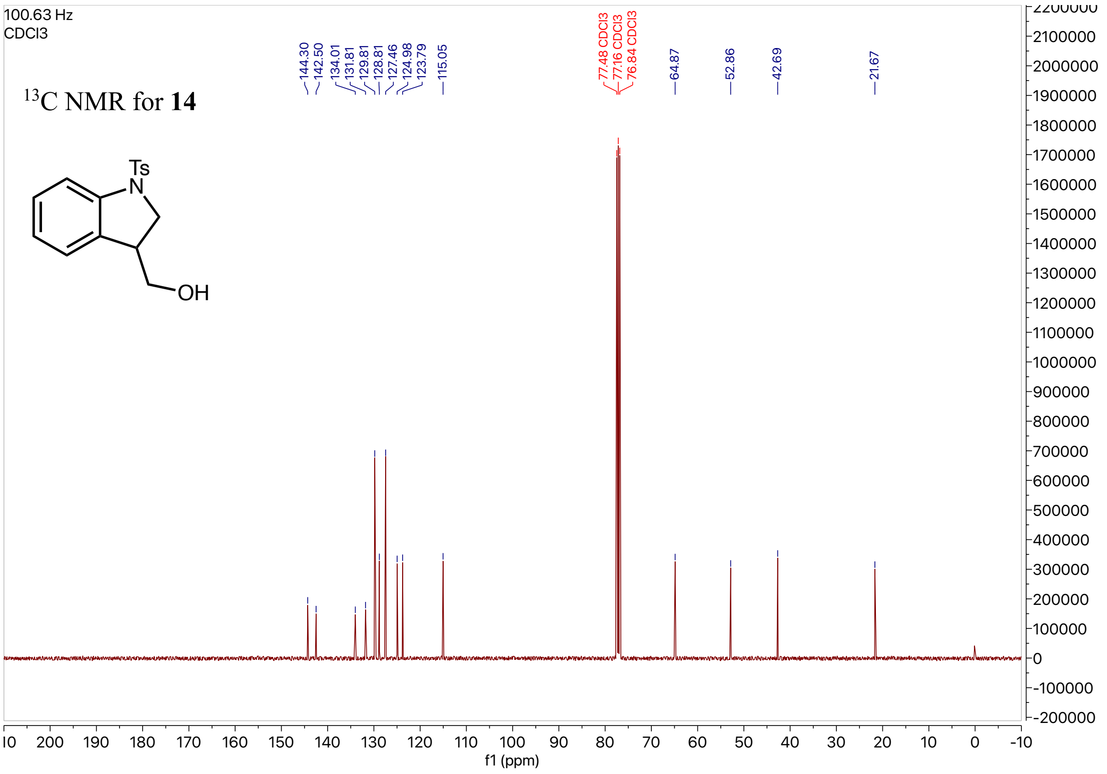
100.63 Hz
CDCl3
13C NMR for 14
144.30
142.50
134.01
131.81
129.81
128.81
127.46
124.98
123.79
115.05
77.48 CDCl3
77.16 CDCl3
76.84 CDCl3
64.87
52.86
42.69
21.67
Ts
N
OH
f1 (ppm)

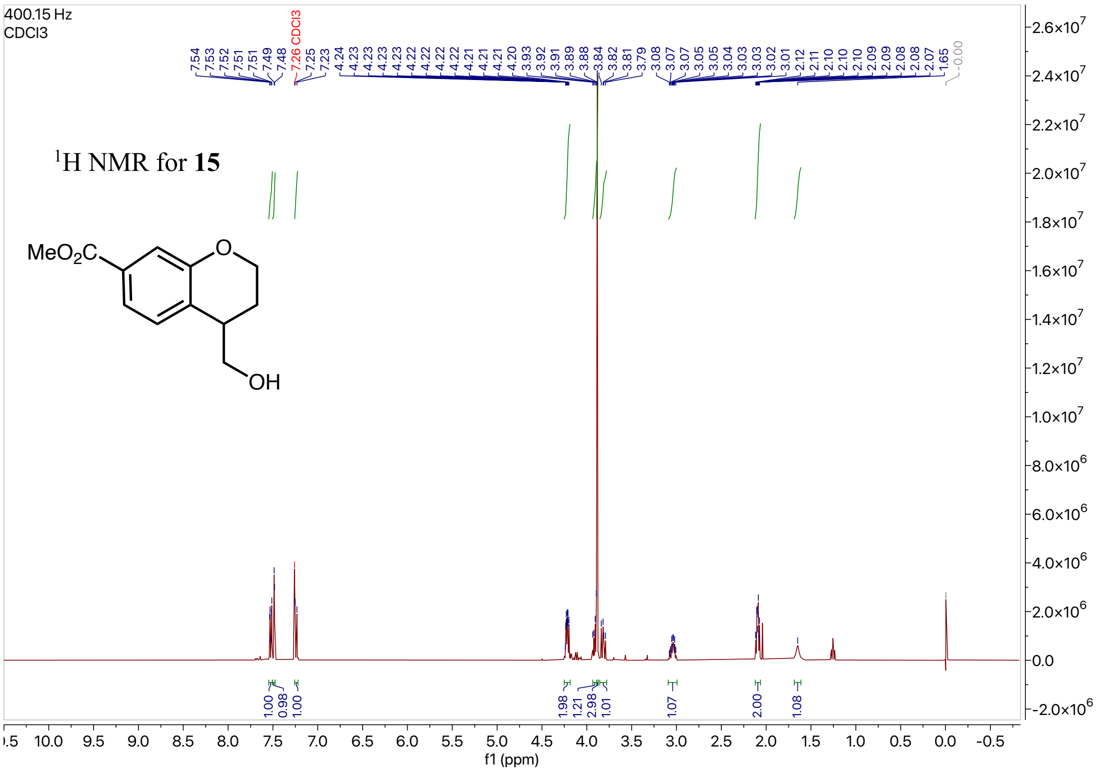
400.15 Hz
CDCl3
1H NMR for 15
MeO2C
O
OH
7.26 CDCl3
1.00
0.98
1.00
1.98
1.21
2.98
1.01
1.07
2.00
1.08
f1 (ppm)

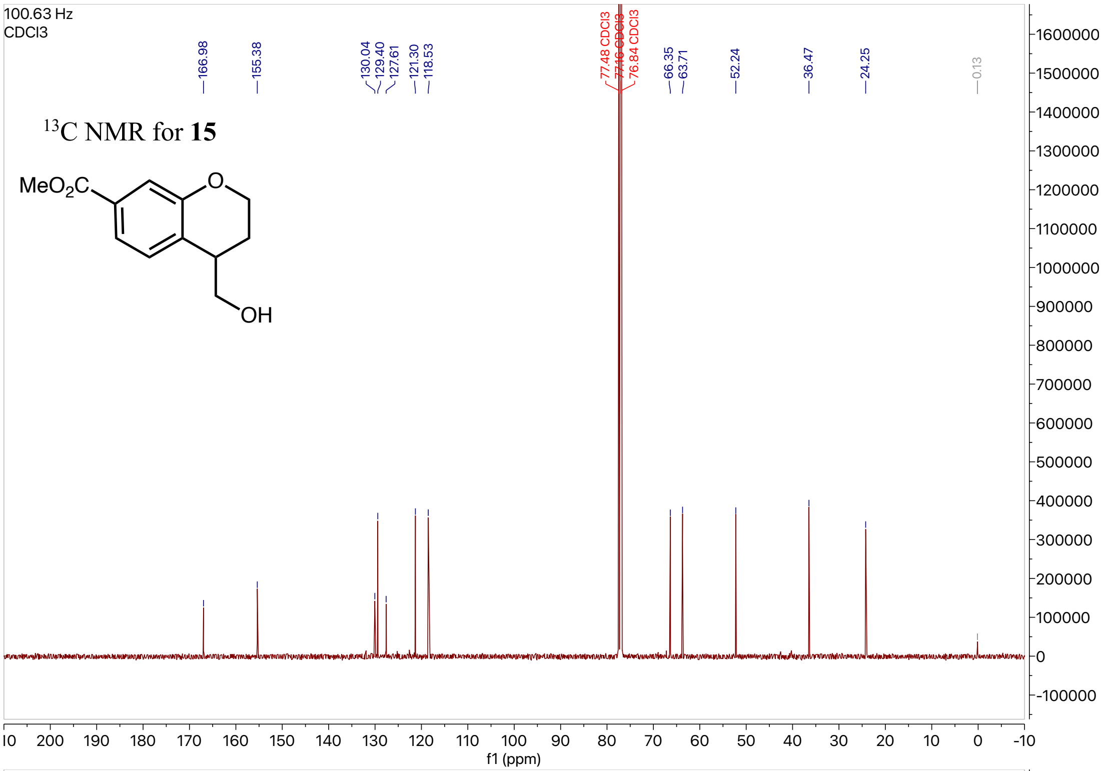
100.63 Hz
CDCl3
166.98
155.38
130.04
129.40
127.61
121.30
118.53
77.48 CDCl3
77.16 CDCl3
76.84 CDCl3
66.35
63.71
52.24
36.47
24.25
0.13
13C NMR for 15
MeO2C
O
OH
f1 (ppm)

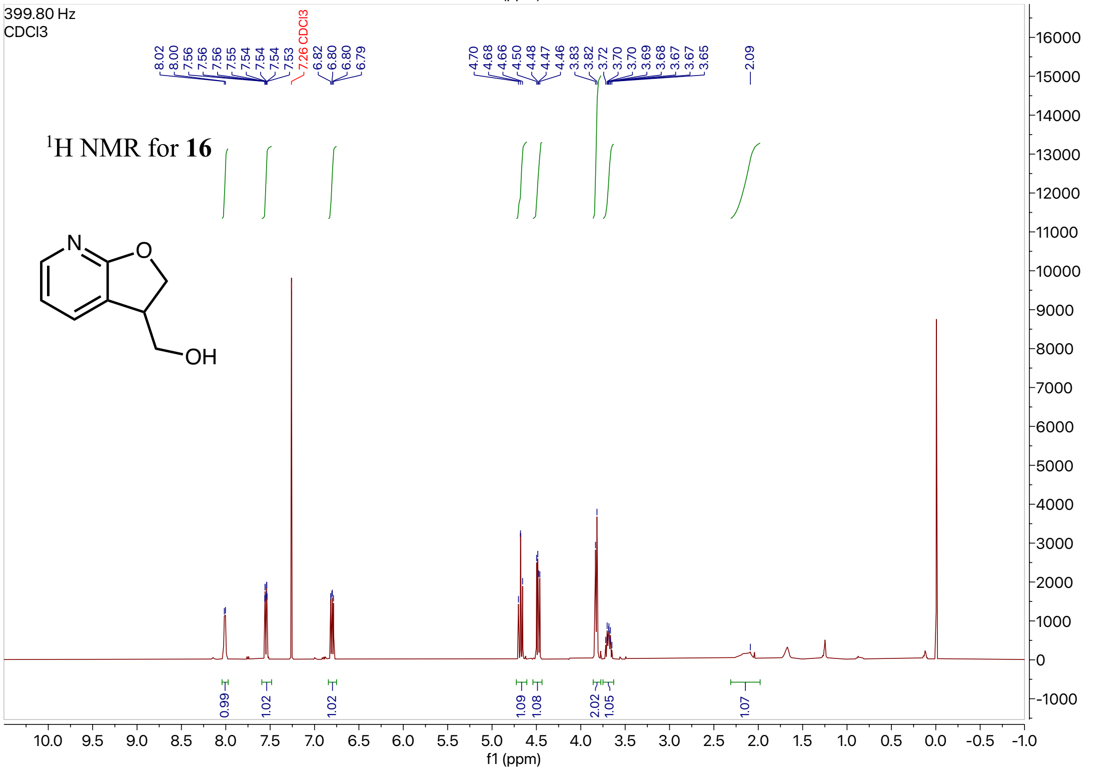
399.80 Hz
CDCl3
8.02
8.00
7.56
7.56
7.55
7.54
7.54
7.53
7.26 CDCl3
6.82
6.80
6.80
6.79
4.70
4.68
4.66
4.50
4.48
4.47
4.46
3.83
3.82
3.72
3.70
3.69
3.68
3.67
3.65
2.09
1H NMR for 16
N
O
OH
0.99
1.02
1.02
1.09
1.08
2.02
1.05
1.07
f1 (ppm)

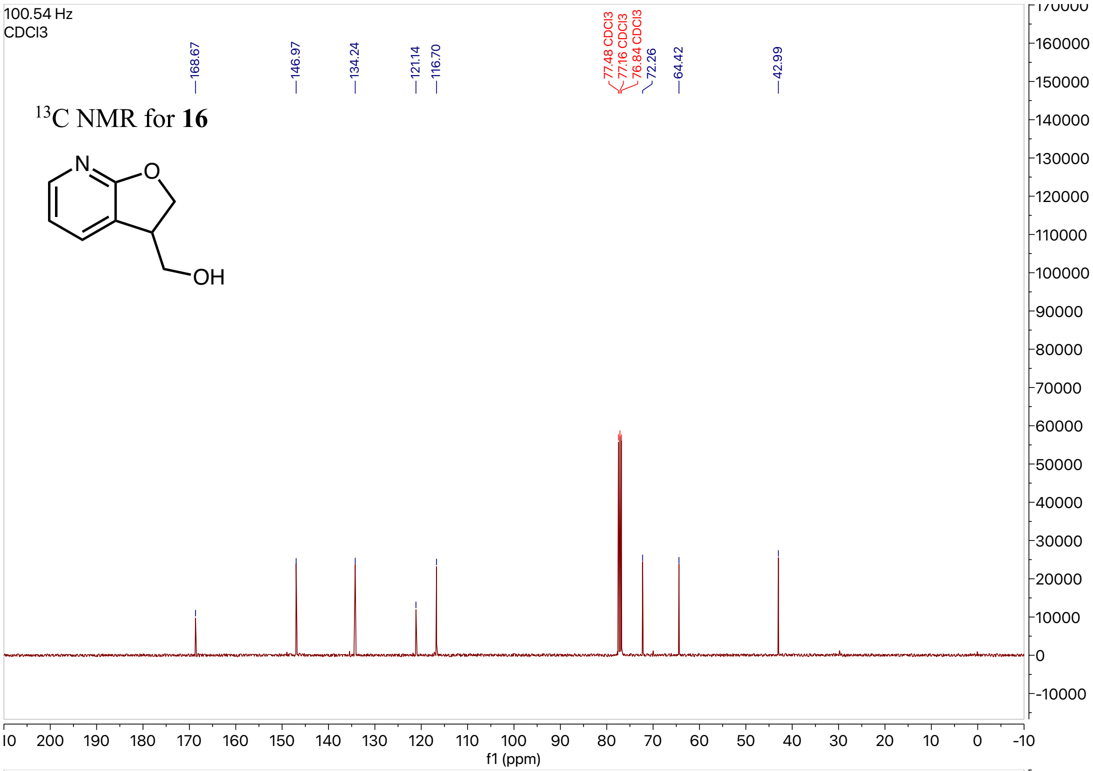
100.54 Hz
CDCl3
168.67
146.97
134.24
121.14
116.70
77.48 CDCl3
77.16 CDCl3
76.84 CDCl3
72.26
64.42
42.99
13C NMR for 16
N
O
OH
f1 (ppm)

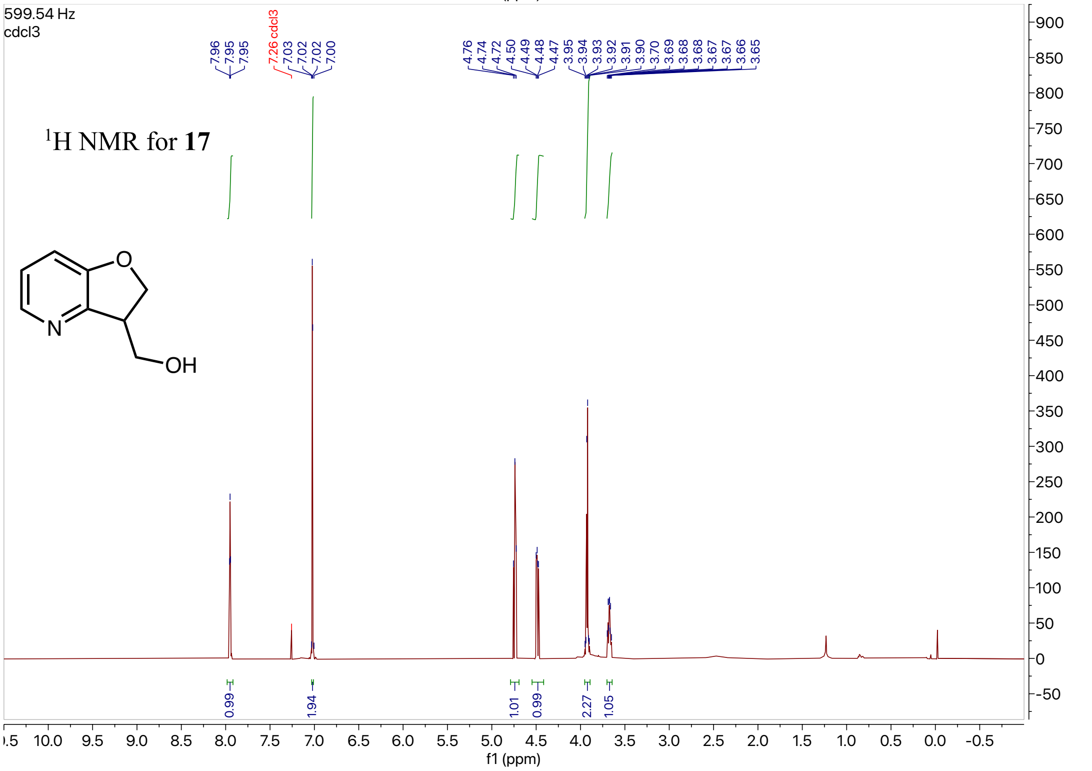
599.54 Hz
cdcl3
7.96
7.95
7.95
7.26 cdcl3
7.03
7.02
7.02
7.00
4.76
4.74
4.72
4.50
4.49
4.48
4.47
3.95
3.94
3.93
3.92
3.91
3.90
3.70
3.69
3.68
3.68
3.67
3.67
3.66
3.65
1H NMR for 17
O
N
OH
0.99
1.94
1.01
0.99
2.27
1.05
f1 (ppm)

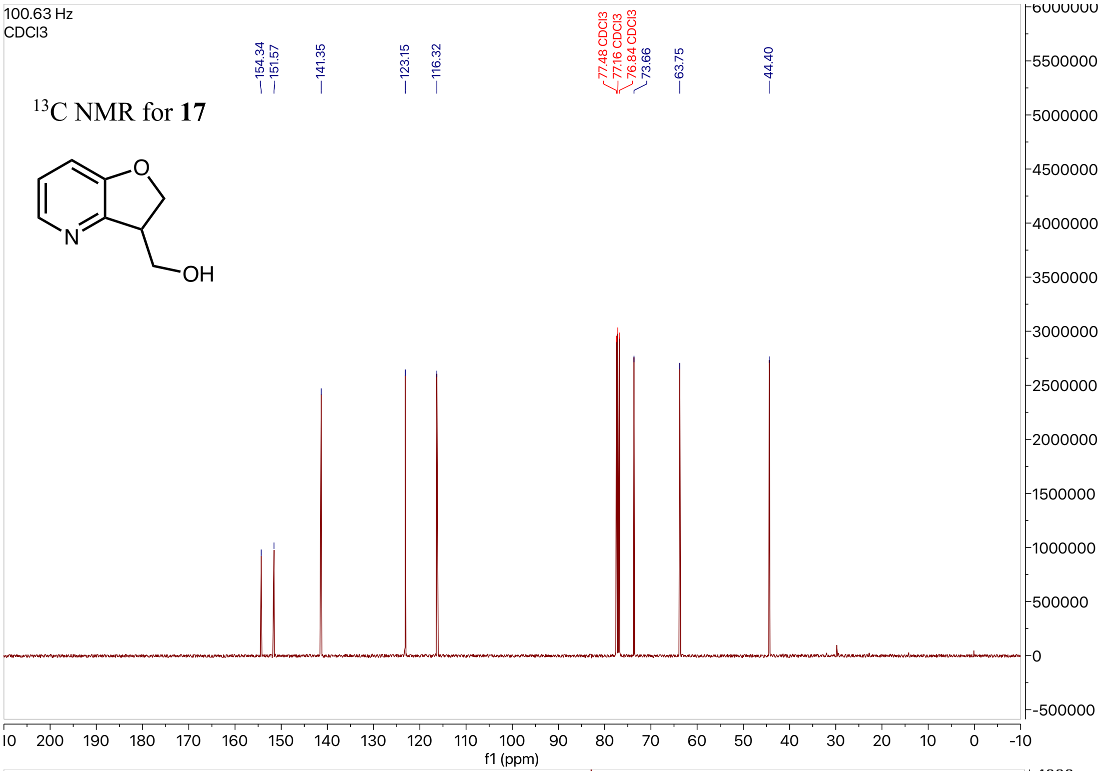
100.63 Hz
CDCl3
13C NMR for 17
154.34
151.57
141.35
123.15
116.32
77.48 CDCl3
77.16 CDCl3
76.84 CDCl3
73.66
63.75
44.40
N
O
OH
f1 (ppm)

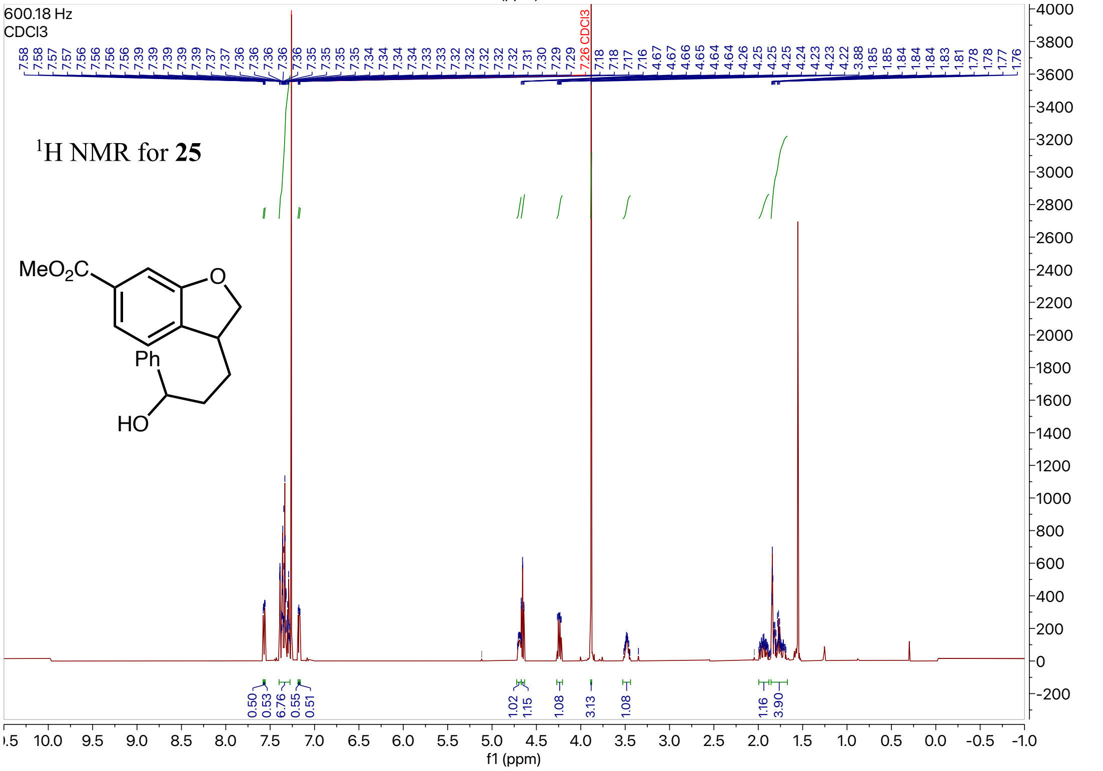
600.18 Hz
CDCl3
1H NMR for 25
7.26 CDCl3
MeO2C
O
Ph
HO
0.50
0.53
6.76
0.55
0.51
1.02
1.15
1.08
3.13
1.08
1.16
3.90
f1 (ppm)

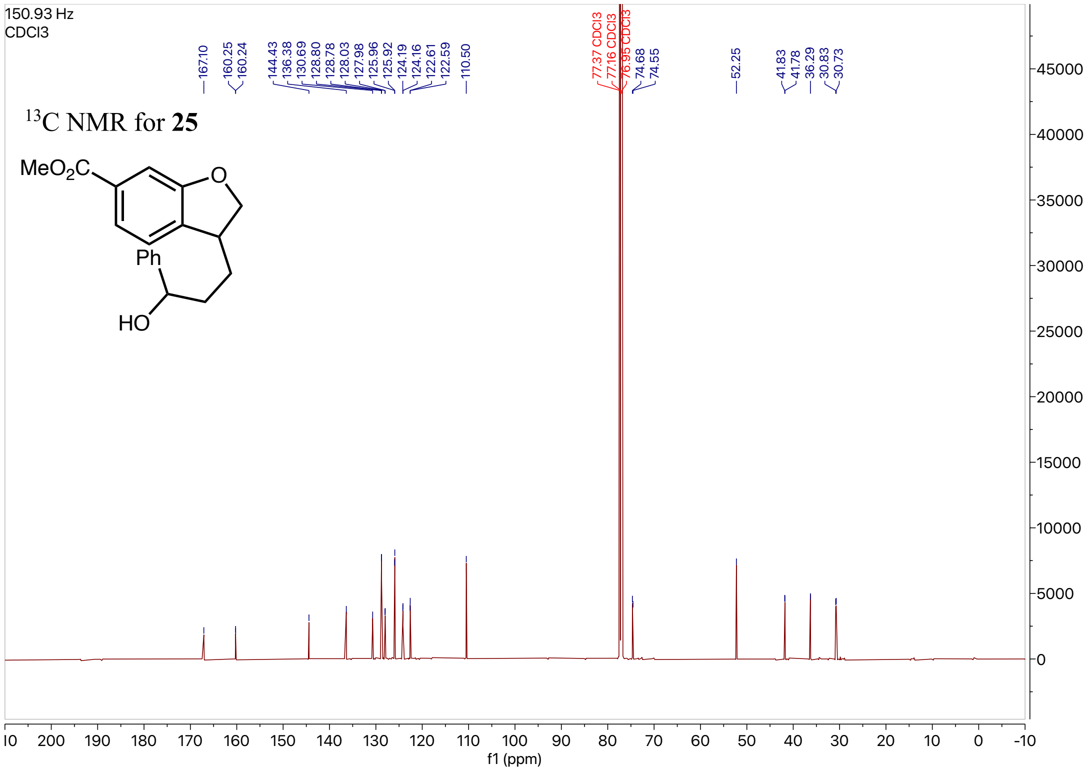
150.93 Hz
CDCl3
167.10
160.25
160.24
144.43
136.38
130.69
128.80
128.78
128.03
127.98
125.96
125.92
124.19
124.16
122.61
122.59
110.50
77.37 CDCl3
77.16 CDCl3
76.95 CDCl3
74.68
74.55
52.25
41.83
41.78
36.29
30.83
30.73
^{13}C NMR for **25**
MeO2C
O
Ph
HO
f1 (ppm)
